# Green inhibitor of carbon steel corrosion in 1 M hydrochloric acid: *Eruca sativa* seed extract (experimental and theoretical studies)†

**DOI:** 10.1039/d2ra01296k

**Published:** 2022-03-23

**Authors:** H. S. Gadow, M. Fakeeh

**Affiliations:** Higher Institute for Engineering and Technology New Damietta Egypt hsgado73@gmail.com

## Abstract

The adsorption activity and inhibition effect of *Eruca sativa* seed extract as a green inhibitor for the dissolution of carbon steel in 1 M hydrochloric acid solution were investigated. In this study, we used a chemical technique (gravimetric method), electrochemical techniques, electrical frequency modulation (EFM), electrochemical impedance spectroscopy (EIS) and potentiodynamic polarization techniques, and theoretical studies. In addition to these techniques, we examined the surface morphology of the carbon steel utilizing different methods. The measurements of the polarization technique indicate that this extract acts as a mixed-type inhibitor. Thermodynamic parameters were calculated and discussed. The adsorption of *Eruca sativa* seed extracts on the alloy obeys the Langmuir and Henry adsorption isotherms. The extract gives an excellent inhibition efficiency 94.8% by a gravimetric method at 0.3 g L^−1^ from the extract. The relationship between the calculated % IE from experiments and the theoretical studies was established.

## Abstract 2

The adsorption activity and inhibition effect of *Eruca sativa* seed extract as a green inhibitor for the dissolution of carbon steel in 1 M hydrochloric acid solution were investigated.

## Graphical abstract

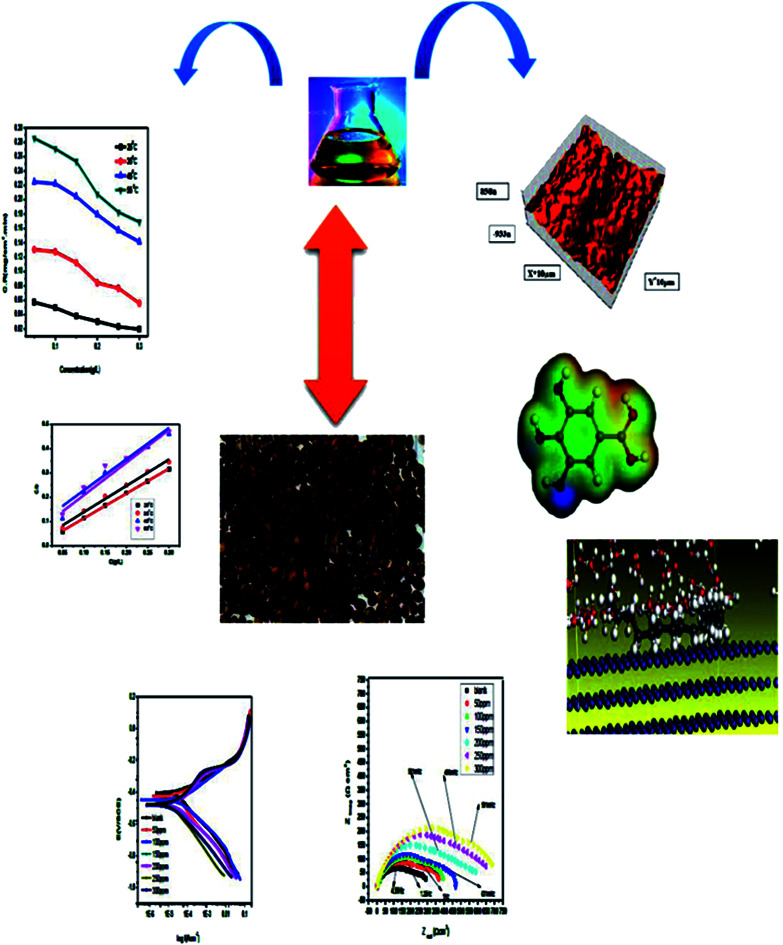

## 1. Introduction

Acidification, acid pickling, water treatment, and chemical cleaning are just a few of the applications for hydrochloric acid, a powerful mineral acid.^1–8^ Carbon steel is widely used as an engineering material around the world, and due to its corrosion resistance, naval applications, oil production and refining, mineral processing, and construction equipment all utilize large volumes of it.^9–11^ Corrosion-prone industrial equipment is cleaned with cleaning materials such as hydrochloric acid and sulfuric acid, which are employed in a variety of cleaning and rust removal operations on the metal surface at the end of activities.^12,13^ Because of its superior mechanical and chemical qualities, carbon steel is widely employed in a wide range of technical and manufacturing applications. However, one of the biggest drawbacks of employing carbon steel is its corrosive nature. Carbon steel corrosion is an unavoidable but manageable phenomenon. Inhibitors are one of the most important additives for preventing carbon steel corrosion. The acid pickling method is a common industrial cleaning process used to remove mineral oxides and mineral scale depositions in petrochemical production and oil-well activities. Nonetheless, because of the very destructive corrosion impact of the mineral acid utilized, this process must be controlled.^14–18^ Organic inhibitors, such as those containing nitrogen, oxygen, sulfur, or carbon, are one of the most widely used strategies for combating metal corrosion, which causes significant economic losses and serious accidents in the industry.^19–26^ Hetero-atoms, conjugate π bonds, and aromatic nuclei, set in organic compounds have been shown to be particularly effective at preventing corrosion in previous studies.^27–36^ They lower the corrosion rate by adsorbing on the metal surface and inhibiting the active sites by displacing water molecules. Organic inhibitors, on the other hand, are toxic, expensive, and harmful to the environment.^37–44^ Plant extracts are thought to be an exceptionally rich supply of natural chemical compounds that may be extracted using simple, low-cost techniques, hence research into employing plant extracts as corrosion inhibitors is a critical scientific endeavor.^45^ As a result, due to both economic and environmental benefits, the study of employing plant extracts as corrosion inhibitors is an important scientific research subject. Several studies have been conducted on the use of plant extracts as corrosion inhibitors. Several leaf extracts, as well as other plant parts, have been proven to be effective corrosion inhibitors for a variety of metals in acid medium.^46–55^ The use of *Eruca sativa* seeds extract as a corrosion inhibitor for carbon steel in acid media was chosen because of its qualities, which include the presence of various organic chemicals that are excellent in the process of preserving metals, as well as its affordability and availability.^56^ The current study will investigate the corrosion prevention of carbon steels by extracting *Eruca sativa* seeds in a 1 M HCl solution using four different techniques: weight loss, electrochemical technology, theoretical research, and surface research.

## 2. Materials and procedures

### 2.1 Reagents

A 37% wt hydrochloric acid (rankem) was utilized to make 6 molar hydrochloric acid, which was tested using 0.1 M sodium carbonate (rankem), and the requisite quantities were attained by dilution. Various solutions containing 1 M HCl were produced using the prepared HCl, either without or with different amounts of *Eruca sativa* seeds extract ranging from 0.05 to 0.3 g L^−1^. The volume of the test solution utilized in our experiments was 50 ml. In order to make the solutions, non-ionized water was employed. A water thermostat was used to set the temperature to 0.50 °C.

### 2.2 The chemical make-up of *Eruca sativa* seed extract

The chemical composition of the plant has been well researched and is universally accepted. The most important chemical compounds discovered are mentioned in Table 1.^57–60^

**Table 1:** *Eruca sativa* seeds extract (names of components, structural formulae, and molecular weights)

Constituents	Chemical structure (the important compounds)	Name	Formula, MW
Flavonoid compounds	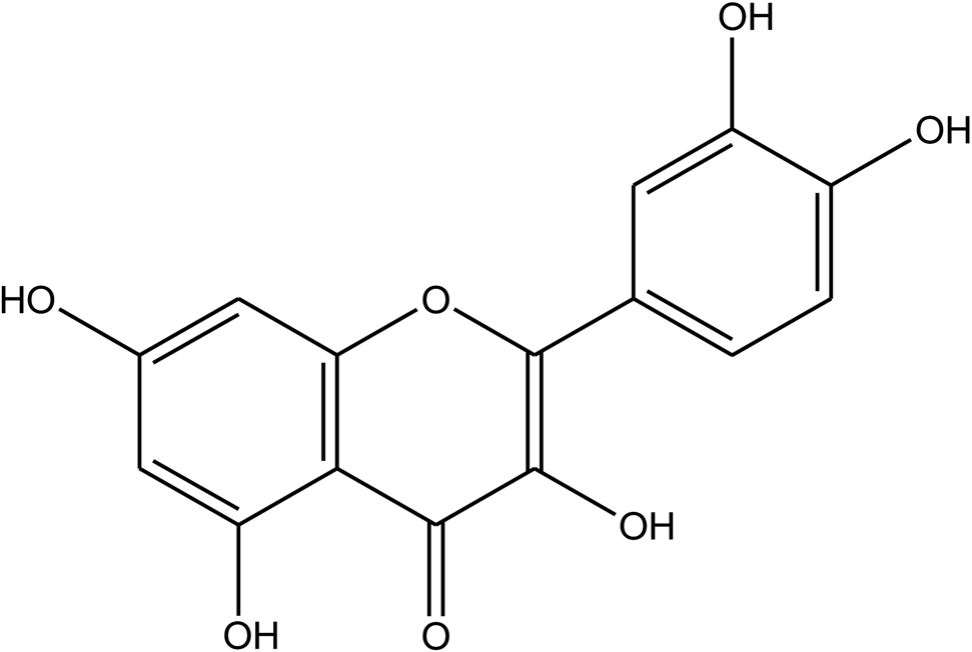	Quercetin	C_15_H_10_O_7_, 302.24
	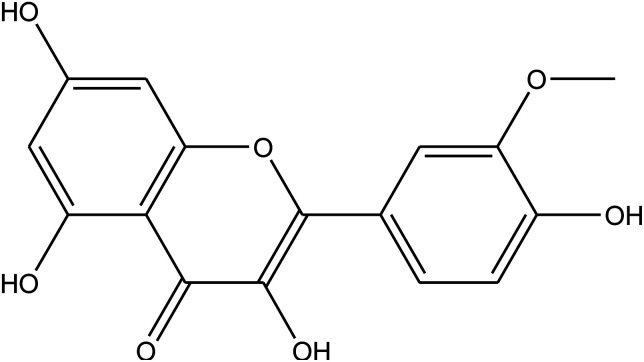	Isorhamnetin	C_16_H_12_O_7_, 316.26
Fatty acids	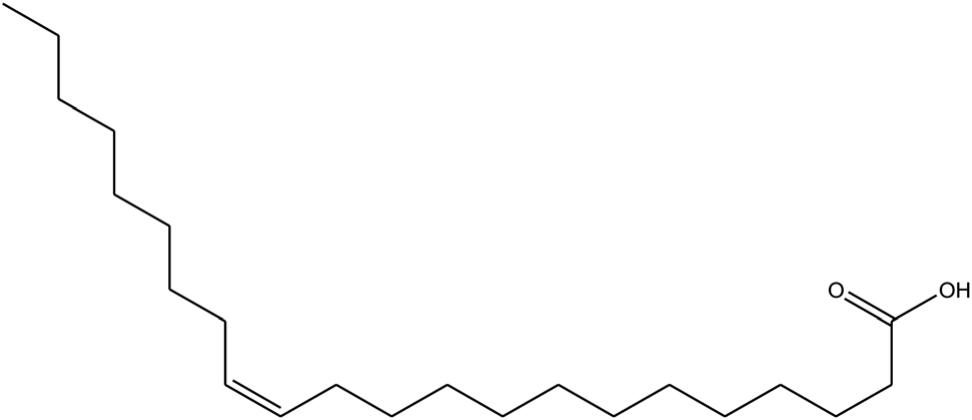	Erucic acid	C_22_H_42_O_2_, 338.32
	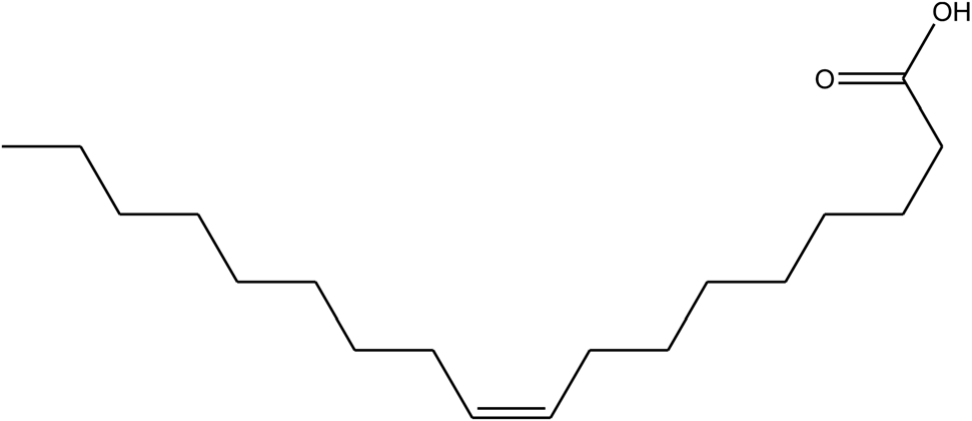	Oleic acid	C_18_H_34_O_2_, 282.46
Phenolic compounds	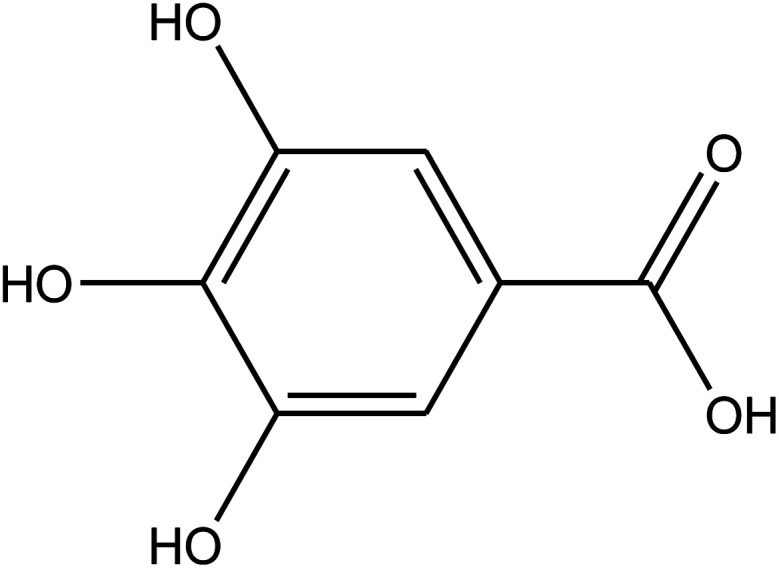	Gallic acid	C_7_H_6_O_5_, 170.12
	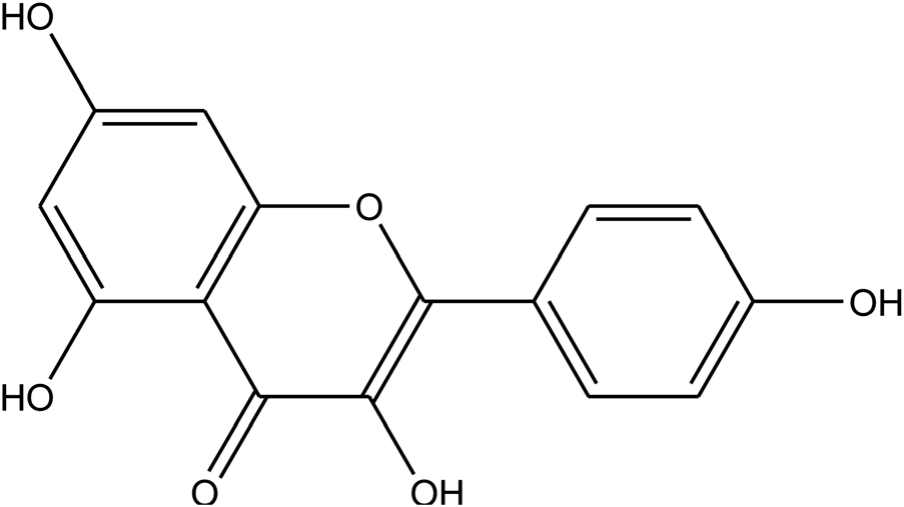	Kaempferol	C_15_H_10_O_6_, 286.24
Ascorbic acid	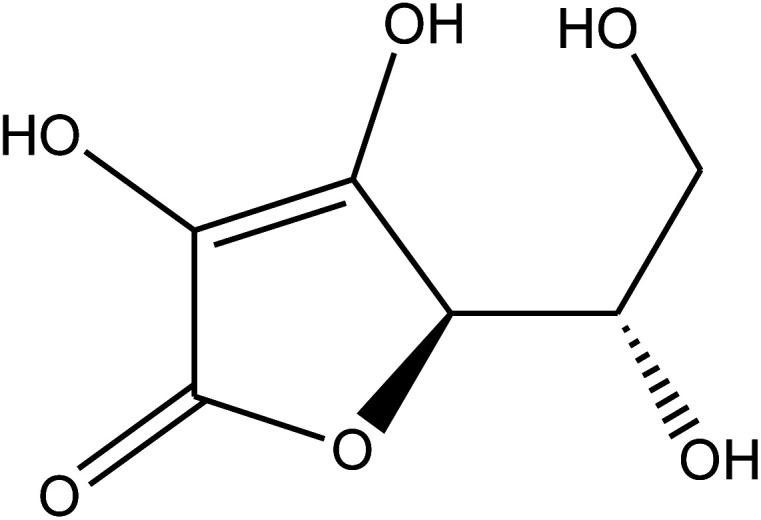	Ascorbic acid	C_6_H_8_O_6_, 176.12

### 2.3 *Eruca sativa* seed extract preparation

Two distinct solvents (85 percent methanol and water with increasing polarity) were used to make organic extracts of seeds.^61^ Dried seeds powder was carefully weighed and utilized to make an extract using a Soxhlet equipment at the appropriate temperature. In a rotary evaporator, the extracted material was filtered and concentrated.^62,63^ To achieve a concentration of 1 g L^−1^, 1000 mL of water was used to dilute 1 gram of *Eruca sativa* seeds extract, and 1 M of hydrochloric acid was prepared as a corrosive medium. Varied volumes of *Eruca sativa* seeds extract (1 g L^−1^) were collected from the stock solution to make different quantities of extract with corrosive media.

### 2.4 Samples preparation

Mn (0.910 percent), C (0.200 percent), P (0.007 percent), Si (0.002 percent), and the remainder iron are present in carbon steel samples. For weight loss (WL) measurements, carbon steel sheets measuring 1 × 1 × 0.2 cm were employed. Finally, by using sandpaper at different degrees (600, 800, 1000, and 1200) the carbon steel sheet was polished. By using distilled water the sheet cleaned, removed with acetone, and dried in accordance with standard requirements.^64^

### 2.5 Mass loss test

After drying it, it was immersed in a one-molar solution of hydrochloric acid with and without varying doses of the *Eruca sativa* seeds extract (0.05, 0.1, 0.15, 0.2, 0.25, and 0.3 g L^−1^) at temperatures variety from 25 to 55 °C, and the weight was measured every half hour for three hours after immersion (this experiment repeated three times). Then, using the eqn (1) and (2), determine the percentage of inhibition (% IE) as well as the surface coverage (*θ*):

1 % IE = *θ* × 100 = [1 − (Δ*m*_inh_/Δ*m*_free_)] × 100

2 *θ* = Δ*m*_free_ − Δ*m*_inh_/Δ*m*_free_ = 1 − Δ*m*_inh_/Δ*m*_free_

where Δ*m*_inh_ is the average weight loss of carbon steel pieces in the presence of *Eruca sativa* seeds extract, Δ*m*_free_ is the average weight loss of carbon steel pieces in the absence of *Eruca sativa* seeds extract. The experiment was repeated three times in order to acquire credible findings. The corrosion rate was calculated using the average weight loss data (C.R). The following equation was used to correctly calculate the C.R:^65^

3 C.R = Δ*m*/*At*

The average weight loss of carbon steel is represented by the symbol (Δ*m* by g), the surface area of carbon steel is represented by the symbol (*A* by cm^2^), and the period of the carbon steel plate in the acidic solution is represented by the symbol (*t*) (immersion time by min).

### 2.6 Electrochemical analysis

#### 2.6.1 Method of potentiodynamic polarization (PDP)

The curves of potentiodynamic were registered at a rate of 0.1 mV s^−1^, mostly between −0.5 V and +0.5 V using saturated calomel electrode as a reference. The inhibitory efficiency and *θ* for *Eruca sativa* seeds extract are determined using the equation below.^66^

4 I.E.% = [(*i*^0^_corr_ – *i*_corr_/*i*^0^_corr_] × 100

5 *θ* = [(*i*^0^_corr_ – *i*_corr_/*i*^0^_corr_]

The dissolution current density measurements (μA cm^−2^) are represented by the symbols *i*^0^_corr_ and *i*_corr_ in the absence and presence of *Eruca sativa* seeds extract, respectively, as obtained by extrapolating both Tafel lines to the corrosion potential.

#### 2.6.2 Measurements using electrochemical impedance spectroscopy (EIS)

At OCP, alternating current indicators with a peak amplitude of 10 mV were utilised with a frequency range of 100 kHz to 0.1 Hz. All EIS findings were evaluated with Gamry Echem software, and the following equation was used to calculate resistance as a measure of inhibition.^67^

6 % I.E. = (1 − *R*_ct(inh.)_/*R*_ct(free)_^0^) × 100

The charge transfer resistances for the *Eruca sativa* seeds extract-inhibited and inhibitor-free systems, respectively, are *R*_ct(inh.)_ and *R*_ct(free)_^0^.

#### 2.6.3 Electrochemical frequency modulation tests (EFM)

We employed the EFM experiment in our research and applied a 10 mV signal between two sinus waves with frequencies ranging from 2 to 5 Hz.^68^ The current density (*i*_corr_), the letters CF-2 and CF-3 stand for causal factors, which are employed as an internal validity check, and the higher peaks were used to determine the slopes of Tafel (*β*_a_ and *β*_c_).^69^

Gamry Potentiostat/Galvanostat/ZRA was utilized in all electrochemical research (PCI4-G750). For PDP, EFM, and EIS calculations, Gamry provides the DC105, EFM140, and EIS300 programs, respectively. To draw, compute, and synthesize value, Echem Analyst sort 5.5 was used. All experiments were carried out in a three-electrode cell, for electrochemical studies, a carbon steel piece served as the working electrode (area of 1 cm^2^) linked to a copper bar and covered with glass, it is treated similarly to the prior weight-loss method, a saturated calomel electrode (SCE) as a reference electrode, and a platinum plate as a counter electrode (CE).

### 2.7. Surface analysis

In these ways, carbon steel coupons were treated similarly to past treating of coupons for weight loss: carbon steel sheets with dimensions of 1 × 1 × 0.2 cm were used. Finally, the carbon steel sheets were polished with sandpaper at various degrees (600, 800, 1000, and 1200). The sheets were cleaned with distilled water, then removed with acetone and dried according to conventional procedures.^64^

#### 2.7.1 Analysis by atomic force microscopy (AFM)

The morphological features of the carbon steel surface were investigated using the atomic force spectroscopy (AFM) technique. Three tests were performed: the first when the carbon steel sheet was polished, the second when the carbon steel sheet was immersed in 1 molar hydrochloric acid, and the third when the carbon steel sheet was immersed in a solution of 1 molar hydrochloric acid and 0.3 g L^−1^ of *Eruca sativa* seeds extract, and we compared the results of the three tests. AFM was carried out with a silicon nitride probe (MLCT model; Bruker) in a contact mode. The scanning parameters were monitored using Proscan software 1.8, and the picture analysis was done using IP 2.1 software.

#### 2.7.2 FTIR spectroscopy

FTIR analysis was used to determine the functional groups exist in the 300 ppm *Eruca sativa* seeds extract +1 M HCl solution, as well as the functional groups exist in the 300 ppm *Eruca sativa* seeds extract +1 M HCl solution after the alloy specimen was submerged for 24 hours. The FTIR examination was performed using an FT/IR-4100, kind A sequence number B117761016.

#### 2.7.3 The surface was examined using XPS

X-ray electronic spectroscopy was used to study the morphology of carbon steel specimens before inundation it in 1 M HCl and then in the existence and in the non-existence of a 0.3 g L^−1^*Eruca sativa* seeds extract for 24 hours, where the study was applied by ESCALAB 250Xi, Thermo-Scientific, United States American.

### 2.8 Quantum chemistry computations

Material Studio's DMol3 module was used to investigate molecules (version 7.0). The popular gradient method (GGA) was added to a core group of double-digit polarisation (DNP) and Becke One substitutable relationship functions (BOP) in the DMol3 unit, and solvent effects were regulated using COSMO control. The two approaches were used to determine certain chemical properties for instance electronegativity (−*χ*), chemical potentials (Pi), smoothness (*σ*), and global hardness (*η*), as stated in the equations below:^70,71^

7 Pi = −*χ*

8 Pi = (*E*_LUMO_ + *E*_HOMO_)/2

9 *η* = Δ*E*/2 = (*E*_LUMO_ − *E*_HOMO_)/2

Softness is determined by universal hardness:

10 *σ* = 1/*η*

We calculated the portion of transported electrons (Δ*N*) using global stiffness and electronegativity, as shown in the following equation:

11 Δ*N* = (*χ*_Fe_ − *χ*_inh_)/2(*η*_inh_ + *η*_Fe_)

The symbols *χ*_in_ and *χ*_Fe_, in that order, represent the absolute electronegativity values for alloy and *Eruca sativa* seeds extract as an inhibitor. The electronegativity readings for iron is 4.28 V mol^−1^, and its hardness is 0 V mol^−1^, respectively, according to several literatures.^72–74^ Furthermore, Fukui function accounts were used to complete the local reactivity of a molecule. The favored locations for electrophilic and nucleophilic assaults of inhibitory compounds are Fukui^+^ and Fukui^−^, respectively.

### 2.9 Simulation of molecular dynamics

The adsorption locator module in Material Studio 7.0 was employed in our work to apply molecular dynamic modelling.^75^ The adsorption locator mimics the process of *Eruca sativa* seed extract particles adsorbing onto a surface of carbon steel. The imitation box (32.27 Å, 32.27 Å, 50.18 Å) was applied to continue the iron (110) across locations with periodic bounds, and each effect traversed a random barrier to mimic an interface model piece. The energy of the investigated *Eruca sativa* seed extract particles was improved using a traditional simulation engine power. The area of Fe (1 1 0) was extended and its rotation was modified by the creation of a supercell after a 20 Å thick vacuum sheet was created on the surface of alloy (1 1 0).^76^ After minimizing Fe surface (1 1 0) and examining the extract particles, a layer function object [native code] built the corrosion system. For atomic simulation studies (COMPASS), the optimal molecular stage was employed as the molecular potential to duplicate the behaviour of *Eruca sativa* extract molecules adsorption on the surface of Fe (1 1 0). Using Monte Carlo simulations, the possibility of adsorption forms of the extract's molecules on the surface of iron (110) was explored, and the adsorption structure as well as its effects on the inhibitory efficacy of the tested extract were determined.^77^

## 3. Discussions and findings

### 3.1 Weight-loss tests (WL)

**Fig. 1:**
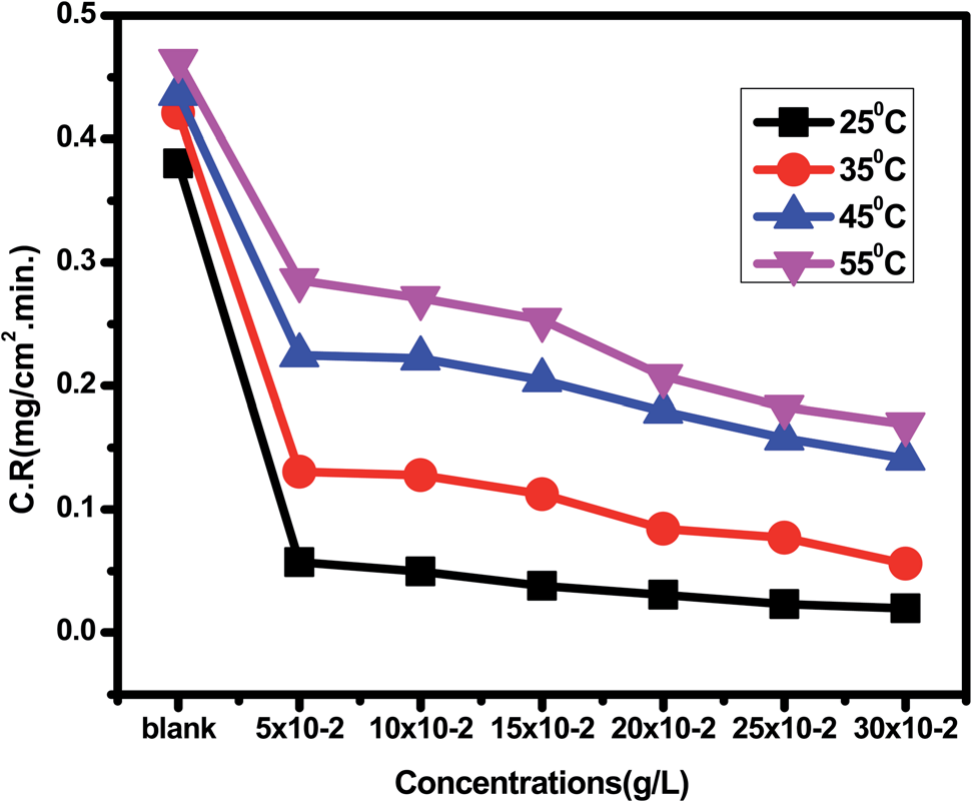
Corrosion rate results of carbon steel electrode dipped in 1 M HCl solution without and with different concentrations (0.05–0.30 g L^−1^) of *Eruca sativa* seeds, after a 90 minute immersion time at various temperatures (25–55 °C).

**Fig. 2:**
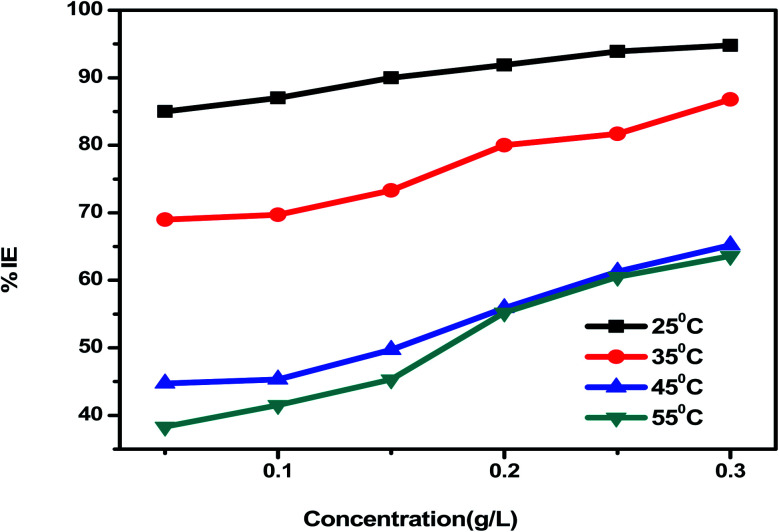
Inhibition efficiency results of carbon steel electrode dipped in 1 M HCl solution containing.

*Eruca sativa* seeds extract was subjected to WL tests, and carbon steel corrosion rates were determined. Carbon steel coupons were soaked in 50 mL of acid solution (1 M HCl) in a hanging posture for 180 minutes in the nonexistence and existence of different concentrations of *Eruca sativa* seeds extract. The carbon steel electrodes were taken from the test solutions every half hour, cleaned with distilled water, dried, and weighed precisely using the usual method. In the absence and presence of *Eruca sativa* seeds extract at different concentrations, Fig. 1 indicates the influence of temperature differential (25–55 °C) on the corrosion rate of carbon steel electrode in acidic conditions (1 M hydrochloric acid). The corrosion rate is lowered when the inhibitor molecule is injected, and the inhibition is increased to 94.8% in presence 300 ppm from *Eruca sativa* seeds extract. It was also revealed that as the temperature climbed, the inhibitory efficiency (I.E.) dropped (see Fig. 2). The increased (percent I.E.) with increasing concentration of *Eruca sativa* seeds extract can be attributed to the adsorbed layer of the extract on the carbon steel surface. The oxygen atoms in the extract have free electron pairs, and the atomic rings' π electrons are responsible for forming this layer.^78^ Because of many differences in the surface of the carbon steel, the effect of temperature on the corrosion process is complicated^79^ (molecules desorption, rapid etching, *etc.*). In the ESI,† the findings are addressed in depth (ESI†) different concentrations (0.05–0.3 g) of *Eruca sativa* seeds after a 90 minute immersion time at various temperatures (25–55 °C).

### 3.2 Parameters of activation through thermodynamics

Found a widespread agreement that corrosion is linked to the Arrhenius equation, in which the activation energy 
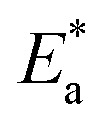
 is calculated by the corrosion rate using the Arrhenius equation (CR).12
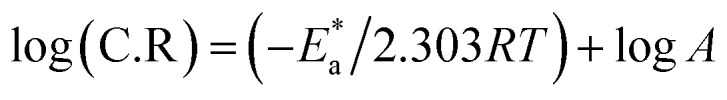


**Fig. 3:**
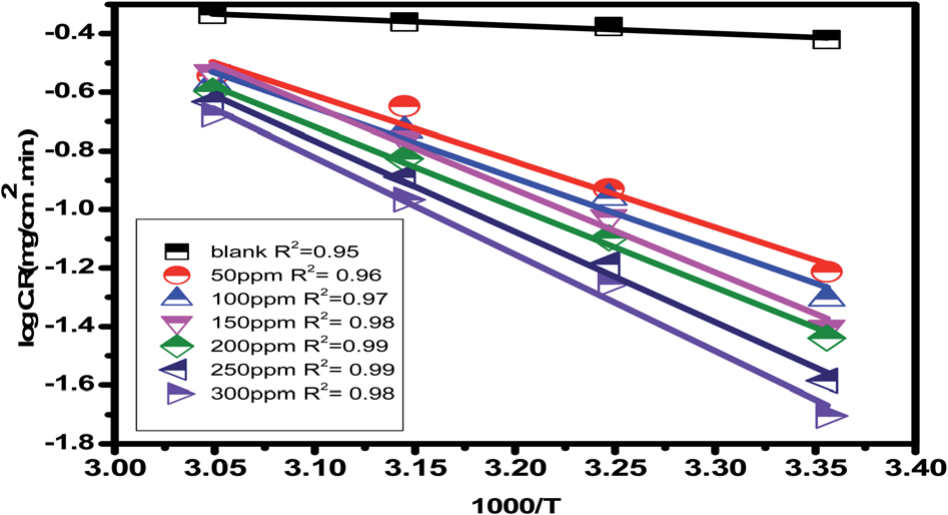
Arrhenius plots for carbon steel at 1 M hydrochloric acid in the presence and absence of different concentrations of *Eruca sativa* seeds extract (log C.R *versus* 1000/*T*).

The universal gas constant has the symbol *R*, the absolute temperature has the symbol *T*, the Arrhenius pre-exponential factor has the symbol *A*, and the corrosion rate has the symbol C.R. Straight lines were created when plotting (log C.R) *vs.* (1/*T*) for the examined *Eruca sativa* seeds extract with the intercept of *A* and slope 
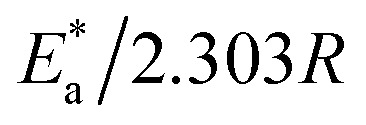
 as shown in Fig. 3; from this, 
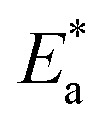
 values could be obtained.

Table 2 reveals that the 
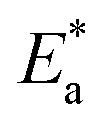
 values rose as the inhibitor concentration was raised, indicating that the *Eruca sativa* seeds extract adsorption on the surface of carbon steel is due to physisorption.^80–82^ The higher value of *E*_a_ in the presence of extract was due to a larger energy barrier. This occurrence also suggests that the inhibitor and carbon steel form a complex combination.^83^ This evidence reveals that the *Eruca sativa* seeds extract can restrict corrosion due to the increased energy barrier for metal dissolving. The activation energy is increased by creating a thin layer on the carbon steel surface that acts as an energy and mass transfer barrier. As a result, the discovery suggests that physical adsorption is responsible for extract adsorption on carbon steel. As shown in the table, the activation energy rose as the extract concentration increased. However, the activation energy was remained higher in the controlled solution than in the uncontrolled solution. These findings are in line with previous research.^84–88^ In both acids, the variation in pre-exponential factor (*A*) was found to be comparable to the variation in apparent activation energy (see Fig. 5). Similar findings had been observed by other investigations.^89–91^ In the current study, higher pre-exponential variables were shown to be associated with higher apparent activation energies, whereas lower pre-exponential variables were found to be associated with lower activation energies. As a result, the tested inhibitor demonstrated good inhibitive effects even at the lowest corrosion activation energies. In both acids, all 
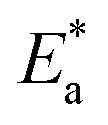
 values are bigger than equivalent ΔH* values, indicating that the corrosion process must involve a gaseous reaction, such as hydrogen evolution. Furthermore, the average difference between activation energy and enthalpy is nearly identical to the average value of *RT*. Furthermore, the average value of the difference between activation energy and enthalpy is nearly identical to the average value of *RT*; where *T* is in the experimental temperature range, showing that the corrosion process is a unimolecular reaction as specified by eqn (13):^92^.

13 *E*_a_ − ΔH* = *RT*

**Table 2:** Parameters of thermodynamic activation of corrosion of carbon steel in 1 M hydrochloric acid in the presence and absence of *Eruca sativa* seeds extract at different concentrations

Inhibitor conc. (g L^−1^)	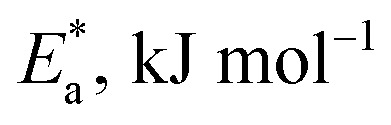	ΔH*, kJ mol^−1^	−ΔS*, J mol^−1^ K^−1^	log *A*
Blank	5.12	18.60	194.74	0.483
0.05	42.89	40.40	131.73	6.35
0.10	45.88	43.85	121.78	6.77
0.15	52.46	48.54	107.66	7.78
0.20	54.15	53.61	93.82	8.11
0.25	59.16	54.50	90.43	8.8
0.30	63.18	58.59	79.65	9.4

The transition state equation yields the activation entropy ΔS* and the standard enthalpy ΔH*.^93,94^

14 log C.R = ΔS*/2.303*R* + ΔH*/2.303*RT* + log(*R*/*Nh*)

**Fig. 4:**
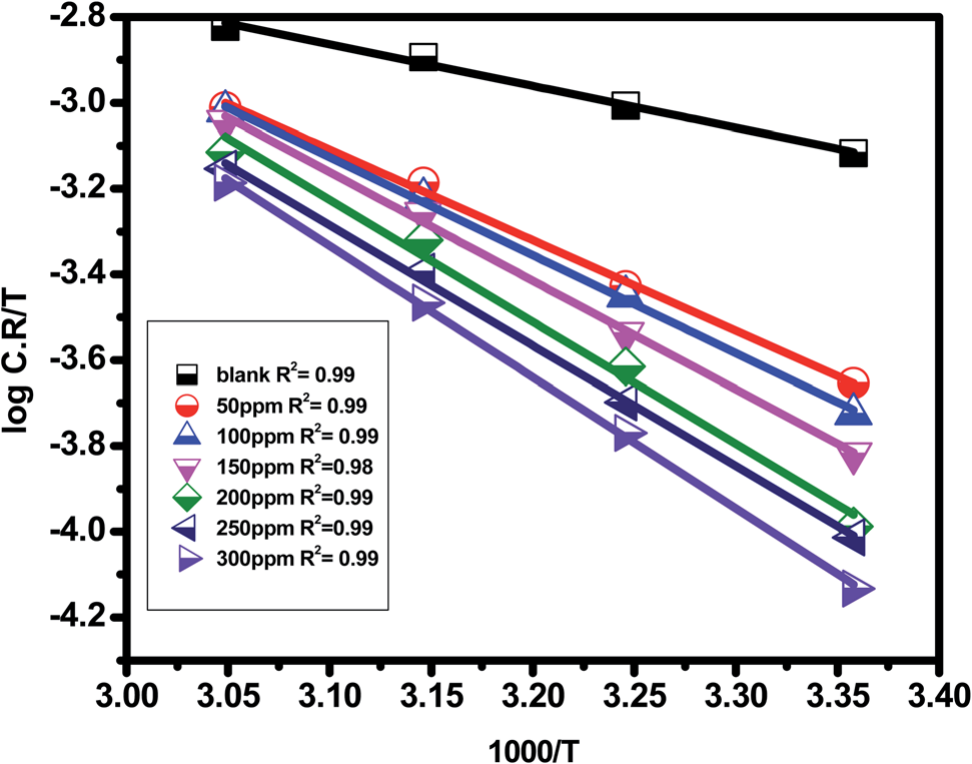
Plots of carbon steel electrode transition states in a 1 M HCl solution containing various amounts of *Eruca sativa* seeds extract.

Avogadro's number is *N*, and Planck's constant is *h*. Plotting (log C.R/*T*) *vs.* (1/*T*) for *Eruca sativa* seeds extract provided straight lines with intercepts of (ln(*R*/*Nh*) + ΔS*/*R*) that were utilized to derive the results of ΔS*, and slopes of (ΔH*/*R*) to attain the values of ΔH*, as shown in Table 2. Fig. 4 shows the extract additions transition step. The positive ΔH* values suggest that carbon steel dissolving is an endothermic and slow process.^95,96^ The sign of ΔS* grows increasingly positive as the quantity of *Eruca sativa* seeds extract increases. Because inhibitor molecules are present in the corrosion reaction media, the activated complexes generated are less ordered, indicating that they are less ordered.^97,98^ As a little amount of inhibitor molecules (50 ppm) was added to the corrosive medium, the 
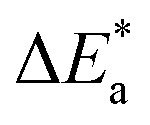
 and ΔH* values skyrocketed from 5.12 to 42.89 kJ mol^−1^ and 18.6 to 40.4 kJ mol^−1^, respectively, when compared to the uninhibited solution. These findings imply that adding extract molecules to the corrosion medium causes a protective layer of inhibitor to form on the carbon steel surface, resulting in a larger energy barrier of the corrosion reaction compared to the uninhibited solution, slowing the corrosion rate.

**Fig. 5:**
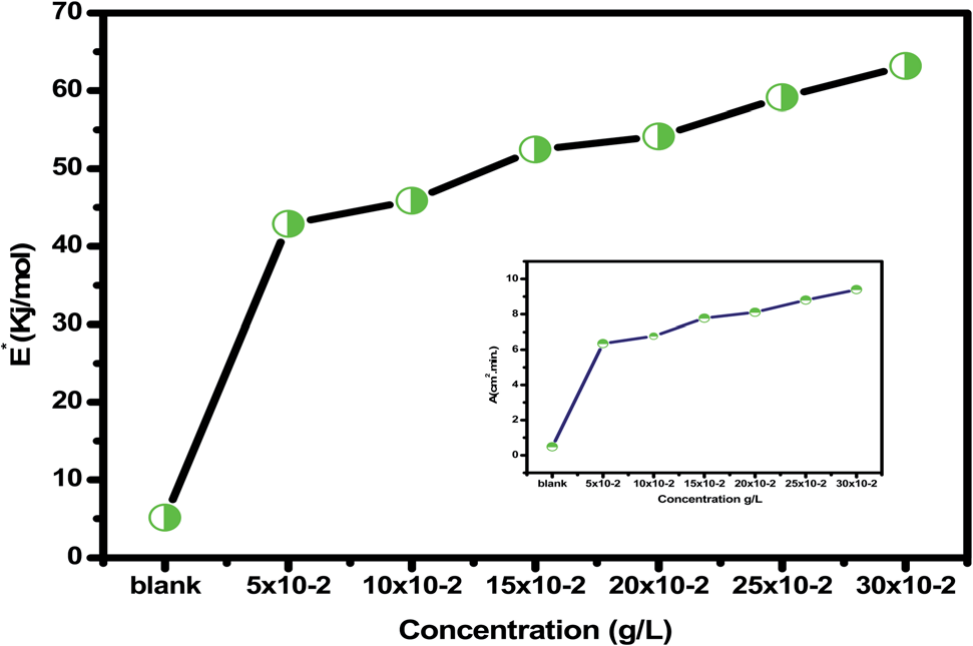
The relation of 
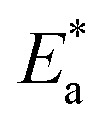
 and *A* with different concentrations of *Eruca sativa* seeds extract at 1 M HCl.

#### 3.2.1 Adsorption research

**Fig. 6:**
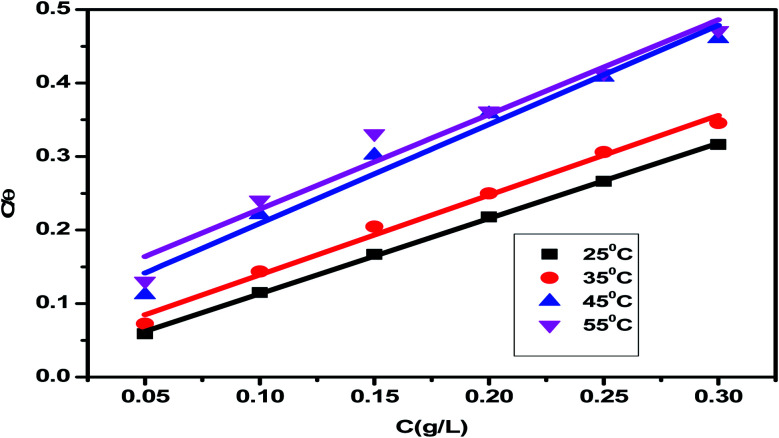
For corrosion of carbon steel in 1 M HCl solution at 25 °C, the Langmuir adsorption isotherm of *Eruca sativa* seeds extract was displayed as *C*/*θ vs.* log *C*.

**Fig. 7:**
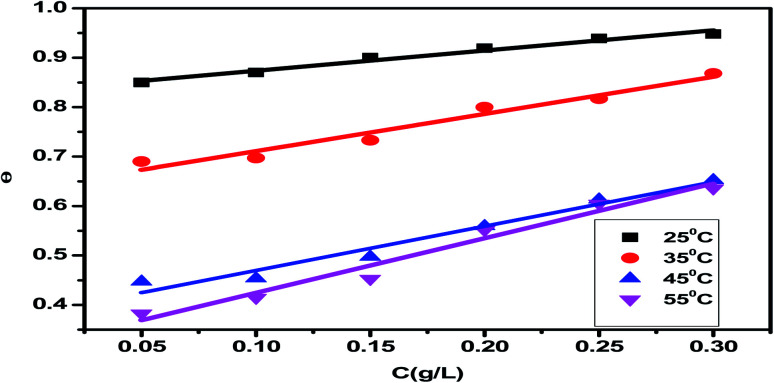
Henry adsorption isotherm of *Eruca sativa* seeds extract represented as *C*/*θ vs.* log *C* for corrosion of carbon steel in 1 M HCl solution at 25 °C.

The adsorption isotherm is a useful tool for simulating the adsorption activity of the studied *Eruca sativa* seeds extract on carbon steel surface. The findings were used to a number of alternative isotherm models for adsorption derived from weight loss tests at various temperatures.^99^ The Langmuir and Henry adsorption isotherms were found to be the most effective in determining the adsorption of inhibitor (Fig. 6 and 7) and Table 3. The two adsorption isotherms are given by eqn (15) and (16) respectively. When picking the optimal adsorption isotherms, the slope values for the investigated isotherms, as well as the computation of the regression coefficient (*R*^2^), are crucial aspects to consider. Because it was closer to the unit, the *R*^2^ value for the Langmiur isotherm was more precise than the Henry isotherm. The ESI† also included further adsorption isotherms curves for *Eruca sativa* seeds extract (Fig. S5–S9†).

15 *C*/*θ* = (1/*K*_ads_) + *C*

16 *θ* = *K*_ads_*C*

where *K*_ads_ is the adsorption equilibrium constant and the concentration of *Eruca sativa* seeds extract is expressed by *C*. In fact, the *K*_ads_ value reflects the strength of the adsorption force between the inhibitor particles and the surface of the carbon steel, that is, the binding force of the alloy surface of the inhibitor particles. A greater *K*_ads_ value, for example, shows that the inhibitor molecules bind to the metal surface more strongly. This one was confirmed in this investigation, which revealed that the equilibrium constant was significantly higher than in earlier studies.^100,101^ Langmuir isotherms were found to provide a better explanation for the adsorption method of *Eruca sativa* seed extract on the carbon steel surface than Henry due to the precision of *R*^2^. The Gibbs free energy adsorption values are intended using eqn (17).

17 *K*_ads_ = 1/55.5 exp(Δ*G*^0^_ads_/*RT*)

**Table 3:** Langmuir and Henry isotherms (*K*_ads_ and Δ*G*^0^_ads_) for different concentrations of *Eruca sativa* seeds extract in 1 M HCl solution

Temp., °C	Langmuir isotherm					Henry isotherm								
	*K*_ads_	*R* ^2^	−Δ*G*^0^_ads_, kJ	−Δ*H*^0^_ads_, kJ	−Δ*S*^0^_ads_, J K^−1^	*K*_ads_	*R* ^2^	−Δ*G*^0^_ads_, kJ	−Δ*H*^0^_ads_, kJ	−Δ*S*^0^_ads_, J K^−1^				
25	17.9	0.999	19.45	41.7	37.22	1.10514	0.97496	11.23	35.16	37.8				
35	16.39	0.990	17.45			0.89543	0.95174	10.33						
45	6.74	0.966	16.26			0.75257	0.95991	9.56						
55	5.02	0.950	14.96			0.40914	0.96384	7.74						

**Fig. 8:**
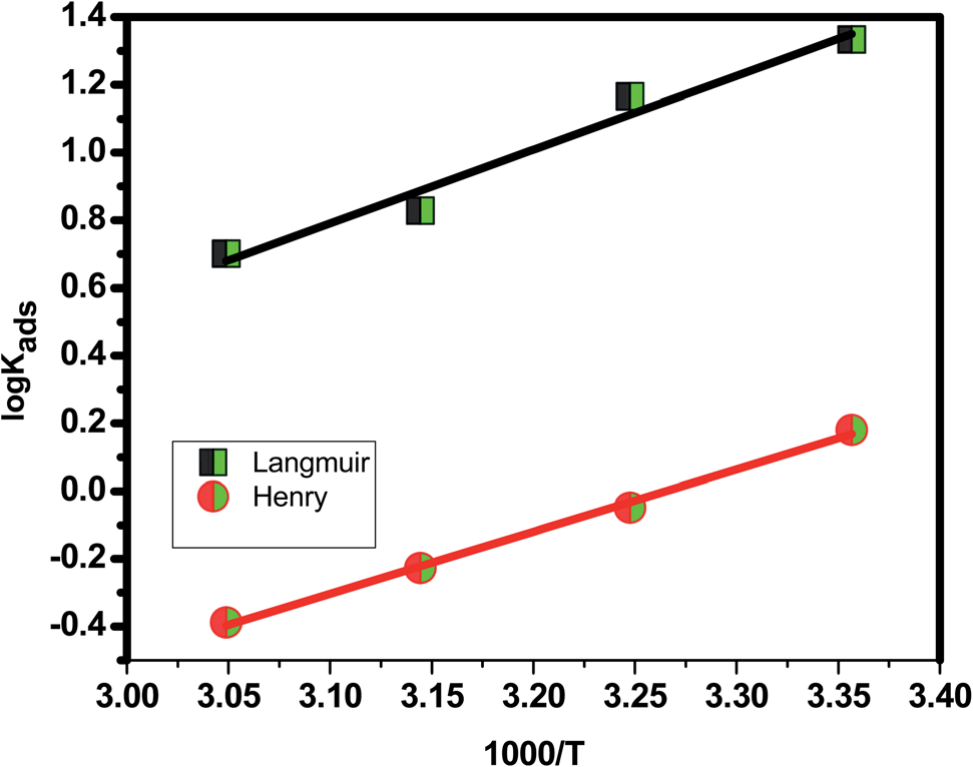
Van't Hoff equation (relation between absolute temperature and adsorption equilibrium constant).

Table 3 shows the negative values of the predicted Δ*G*^0^_ads_, indicating that carbon steel spontaneity is a temperature-dependent exothermic process in which an increase in the reaction temperature causes the inhibitor to adsorb off the steel's surface.^102^ The two adsorption modes typically mentioned in terms of corrosion inhibition are chemisorption and physisorption.^103^ The electrostatic interaction between charged molecules (anti-ions) and the negatively charged metal surface is known as physical adsorption. Δ*G*^0^_ads_ values up to −20 kJ mol^−1^ are consistent with physical adsorption; however, between −20 kJ mol^−1^ and −40 kJ mol^−1^, chemical adsorption tends to be stronger than physical adsorption when the reading heads are set to −40, but beyond −40 kJ mol^−1^, only chemical adsorption occurs.^102^ The integrated version of the van't Hoff equation can be used to compute thermodynamic adsorption parameters such as adsorption enthalpy Δ*H*^0^_ads_ and entropy of adsorption Δ*S*^0^_ads_ (Fig. 8).^104^

18 ln *K*_ads_ = (−Δ*H*^0^/*RT*) + (Δ*S*^0^_ads_/*R*) + ln(1/55.5)

The exothermic nature of *Eruca sativa* seeds extract adsorption on carbon steel surfaces is indicated by the negative sign of Δ*H*^0^_ads_. An exothermic adsorption process is associated with negative Δ*S*^0^_ads_ values. This matches the expected result, which stipulates that while adsorption is an exothermic process, it must be followed by a decrease in entropy change, and *vice versa*.^83^

### 3.3 Potential measurements in an open circuit (OCP)

**Fig. 9:**
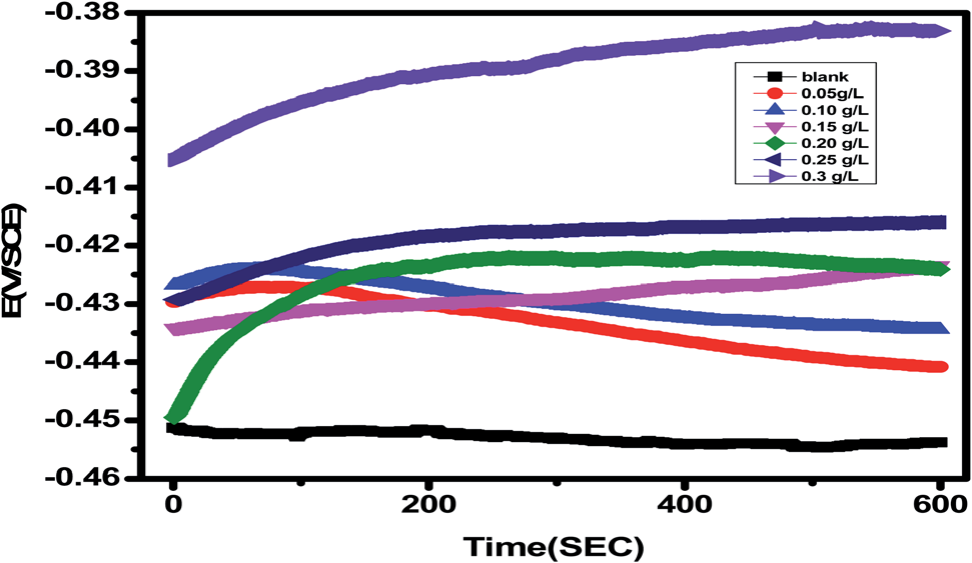
OCP potential–time curves for carbon steel in 1 M HCl at non-existence and presence of different concentration of *Eruca sativa* seeds extract.

In the absence and presence of different amounts of *Eruca sativa* seed extract, as illustrated in Fig. 9, the fluctuation of the open circuit potential of carbon steel electrodes as a function of time was compared to a saturated calomel electrode (SCE). In 1.0 M HCl solution, the carbon steel electrode's corrosion potential (*E*_corr_) first moves toward greater negative values, resulting in a transient step, as illustrated in the figure. In the presence of *Eruca sativa* seeds extract solutions, during the immersion, the open circuit potential values shifted towards larger positive potentials. The adsorption of *Eruca sativa* seeds extract on the carbon steel surface explains this. The results then tend towards stability, showing that molecule adsorption and desorption in the *Eruca sativa* seeds extract had reached a dynamic equilibrium.^105,106^ This indicates that in the existence of *Eruca sativa* seeds extract, the kinetics of the anodic reaction of carbon steel in 1 M hydrochloric acid were significantly influenced.^105^

### 3.4 PDP measurements (potentiodynamic polarisation)

**Fig. 10:**
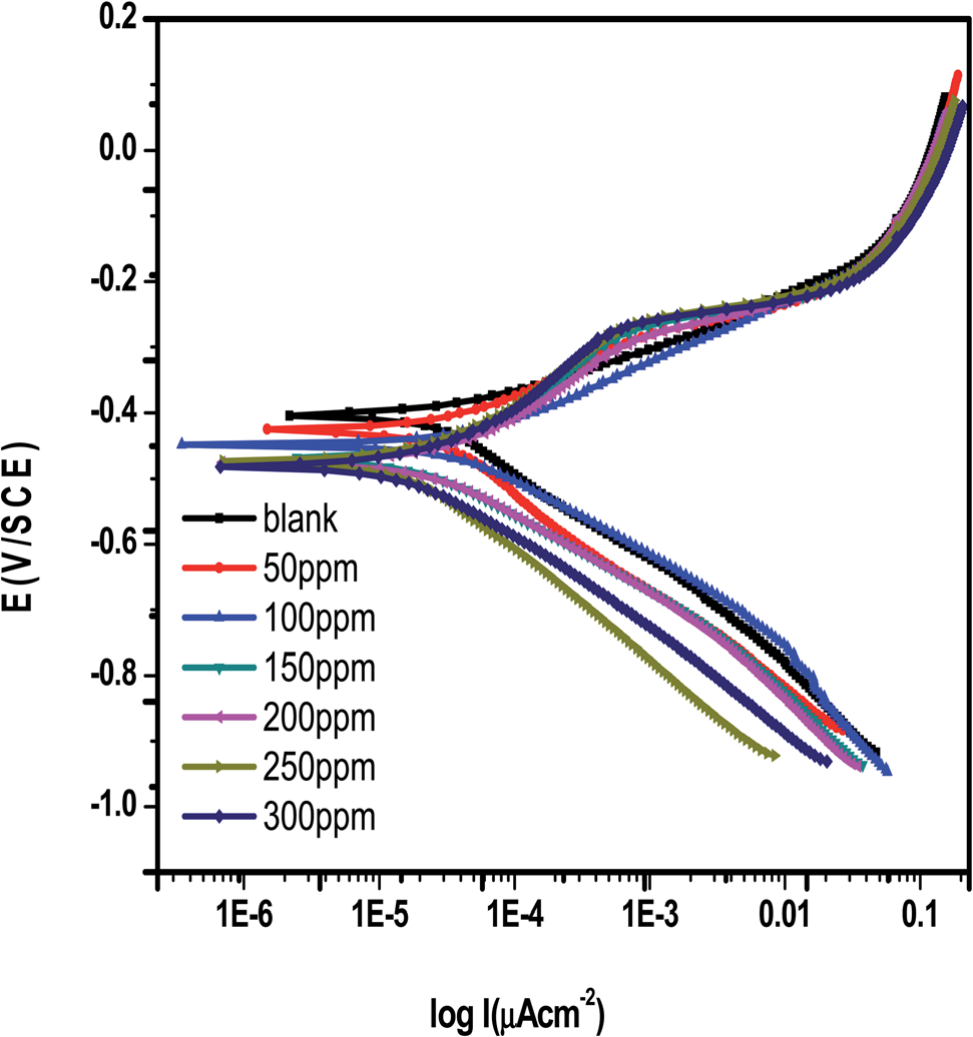
Potentiodynamic polarisation technique curves for corrosion of carbon steel in 1 M HCl in the absence and presence of different concentrations from *Eruca sativa* seeds extract at 25 °C.

Fig. 10 shows the PDP curves for carbon steel in 1 M hydrochloric acid with and without various doses of *Eruca sativa* seeds extract (0.05–0.3 g L^−1^) at 25 °C. The cathodic and anodic curves shifted to lower current density values in the presence of the extract, resulting in a reduction in carbon steel corrosion rate. The presence of extract considerably lowered the density of corrosion currents. The Tafel plot showed a movement towards the cathodic zone. This suggested that the extract had a repressive effect on the cathodic process. The presence of *Eruca sativa* seeds extract causes variations in the profiles of anodic curves, indicating that this extract has an effect on the anodic process. As a result, extract has a dual inhibitory effect. The formation of a barrier layer on the carbon steel surface could explain the profile disparity. Table 4 shows the polarization results from the extract, including current density of corrosion (*i*_corr_), potential of corrosion (*E*_corr_) for blank and inhibited samples at numerous concentrations, cathodic and anodic Tafel slopes (*β*_c_ & *β*_a_), and inhibition efficiency (percent I.E.). The cathode and anode curve forms are quite comparable in these parameters, showing that the techniques of dissolving carbon steel and hydrogen reduction have remained unchanged in the presence of *Eruca sativa* seeds extract. The results also demonstrated that, alter the Tafel anode and cathode slopes (*β*_a_ and *β*_c_) slightly as the concentration of the tested extract was raised. In the presence and absence of *Eruca sativa* seeds extract, the mechanism of inhibition did not alter.^107,108^ The following relation was used to compute inhibition efficiency.^109^

19 % I.E. = (*i*^0^_corr_ − *i*_inh._/*i*_corr_) × 100

**Table 4:** Shows the effect of the concentration of *Eruca sativa* seeds extract on the parameters of potentiodynamic method tests for carbon steel corrosion in 1 M HCl at 25 °C

Conc.,g L^−1^	−*E*_corr_, mV *vs.* SCE	*R*_p_, ohm	*I*_corr_, μA cm^−2^	*β*_a_, mV dec^−1^	−*β*_c_ mV dec^−1^	*θ*	% I.E.
Blank	480.3 ± 5	17.75 ± 0.5	358.2 ± 3	135.5 ± 2	186.2 ± 5	—	—
0.05	466.0 ± 3	20.40 ± 0.4	42.8 ± 0.6	132.5 ± 1	182.6 ± 3	0.881	88.1
0.1	478.0 ± 6	23.14 ± 0.3	39.6 ± 0.3	129.5 ± 4	184.7 ± 4	0.889	88.9
0.15	470.0 ± 4	24.51 ± 0.6	32.3 ± 0.5	135.0 ± 3	176.2 ± 2	0.909	90.9
0.2	476.5 ± 2	24.77 ± 0.2	28.5 ± 0.2	137.2 ± 5	180.3 ± 4	0.920	92.0
0.25	475.5 ± 4	26.07 ± 0.3	24.3 ± 0.3	138.9 ± 2	184.3 ± 3	0.932	93.2
0.3	481.5 ± 5	36.80 ± 0.4	22.5 ± 0.2	137.2 ± 4	178.5 ± 2	0.937	93.7

**Fig. 11:**
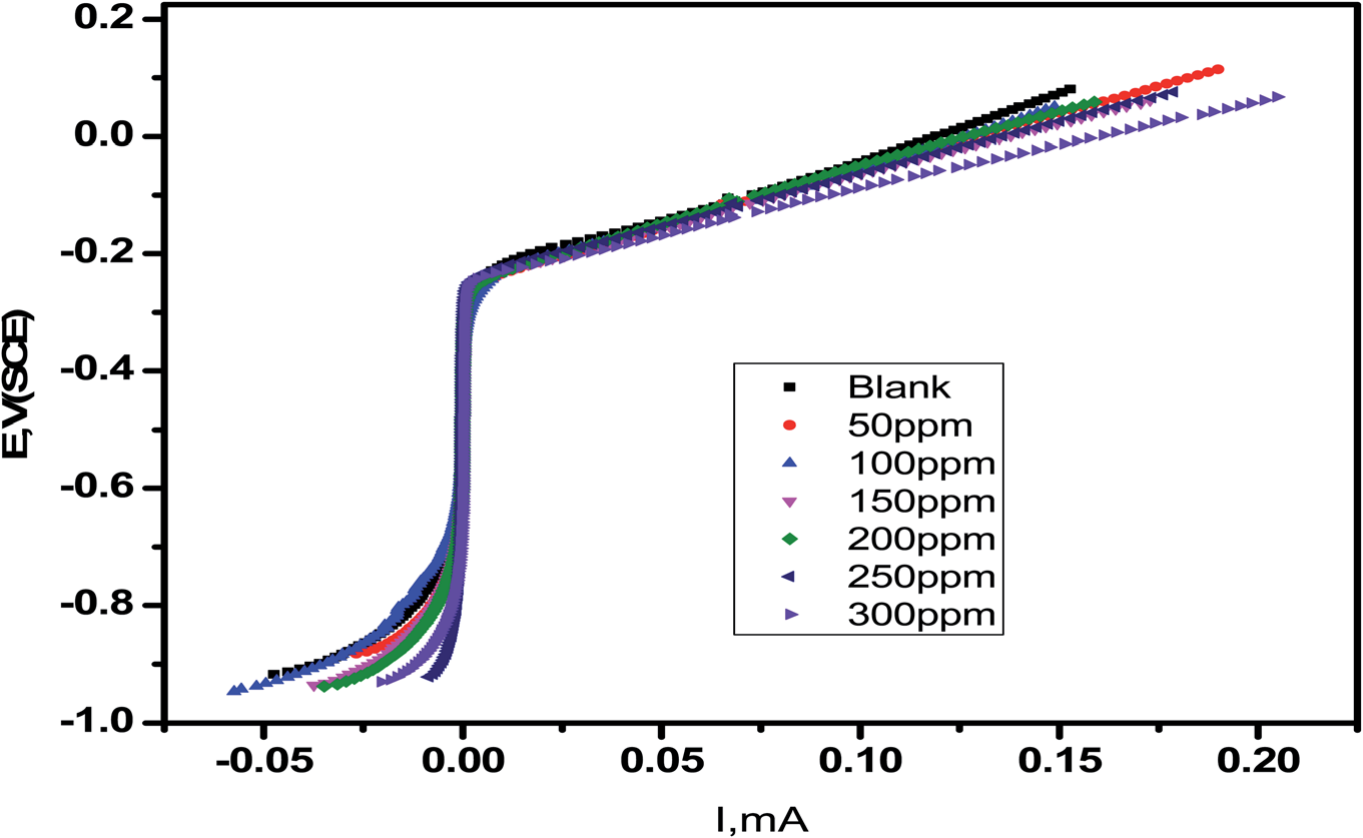
Carbon in 1 M HCl plotted by linear sweep voltammetry with a different dose of *Eruca sativa* seeds extract.

The corrosion current densities of carbon steel with and without different concentrations from *Eruca sativa* seeds extract are denoted by *i*^0^_corr_ and *i*_inh._, respectively. As demonstrated in Table 4, the higher the inhibitor concentration, the higher the percentage inhibition values with decreasing (*i*_inh_).^109^ If *E*_corr_ is greater than −85 mV per SCE when compared to the uninhibited blank's corrosion potential, the inhibitor can be either anodic or cathodic; however, if *E*_corr_ is less than −85 mV per SCE, the inhibitor can be considered mixed.^110^ In this experimental, the change was fewer than −15 mV for each SCE, indicating that the extracts under investigation are mixed type inhibitors.^111^ As shown in Fig. 11, increasing the concentration of *Eruca sativa* seeds extract increased the polarization resistance due to the extract's adsorption on the carbon steel surface.^112^

### 3.5 Measurements using electrochemical impedance spectroscopy (EIS)

**Fig. 12:**
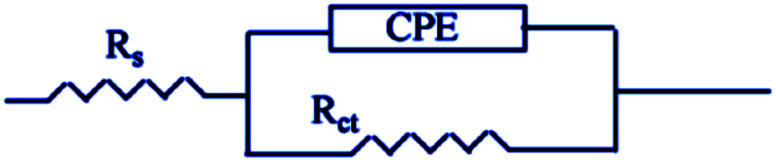
A model of an equivalent circuit for measuring EIS data.

EIS studies provide information on the kinetics of electrode operations as well as the surface properties of the systems being studied. The geometry of the impedance diagram can be used to deduce information.^113^ In a 1 M HCl solution at 25 °C and 30 minutes of immersion in the existence and absence of varied extract concentrations, the EIS technique was utilised to investigate the carbon steel corrosion efficiency. Fig. 12 depicts a comparable circuit that examines all of the processes involved in the system's electrical response by using a parallel combination of charge-transfer resistance (*R*_ct_) and constant phase element (CPE), both in series with the solution resistance (*R*_S_). Surface heterogeneity is caused by surface roughness impurities, dislocations, grain boundaries, inhibitor adsorption, and porous layer development. The CPE element is used to explain the depression of the capacitance semi-circle, which correlates to surface heterogeneity.^114^ The next equation gives the CPE impedance.:

20 *Ζ*_CPE_ = 1/*Υ*^0^(*jω*)^*n*^

**Fig. 13:**
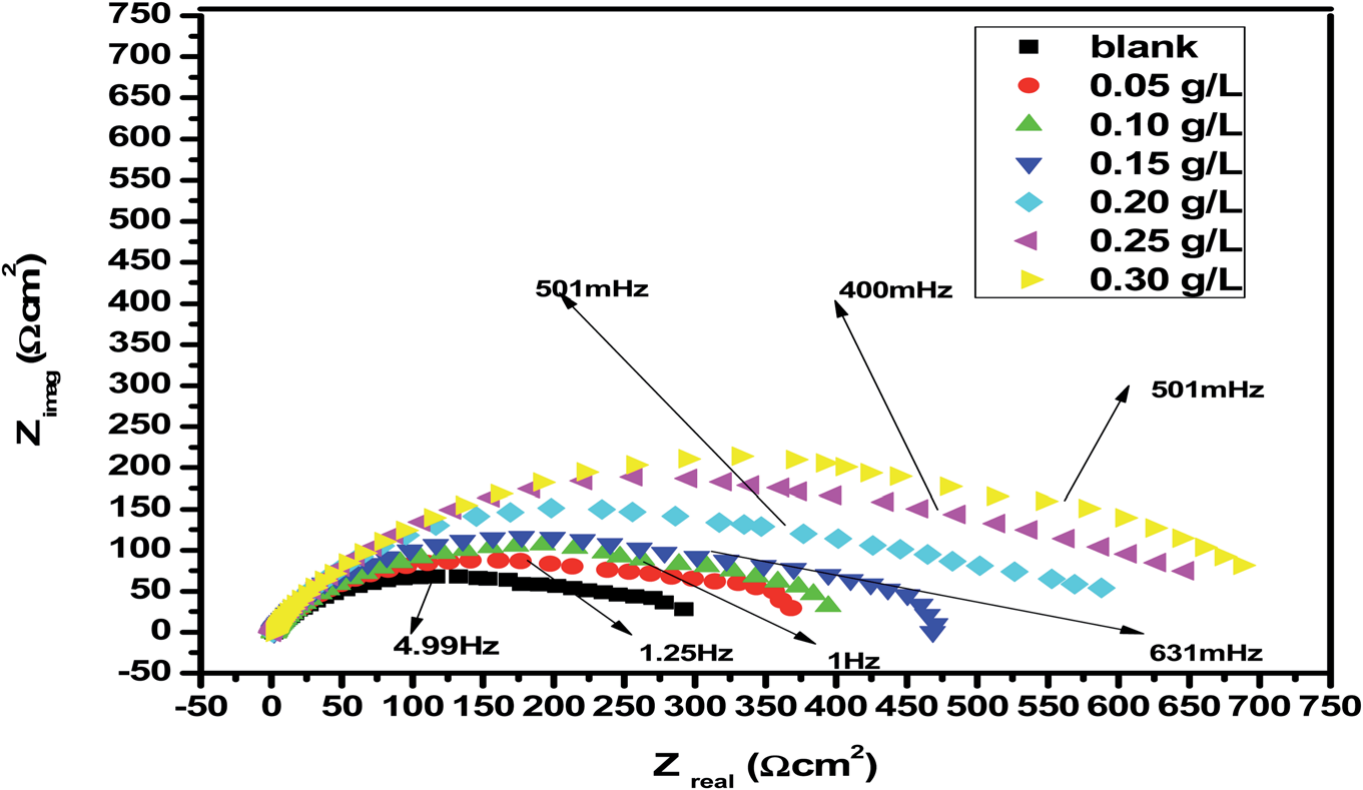
Nyquist plots for corrosion of carbon steel at 1 M HCl in the absence and presence of different concentrations of *Eruca sativa* seeds extract at 25 °C.

**Fig. 14:**
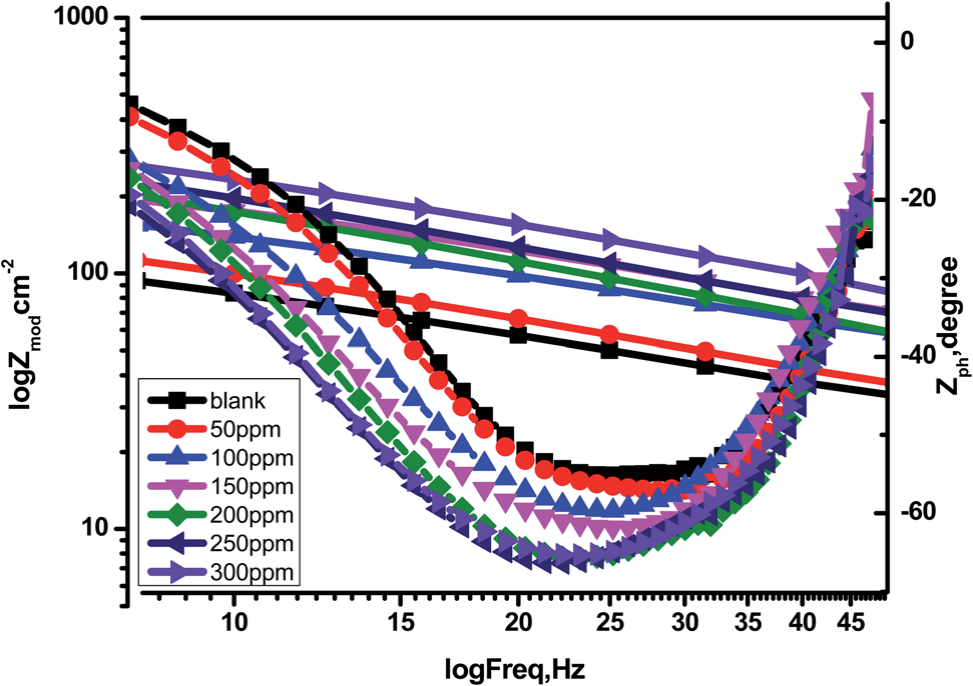
Bode charts for the corrosion of carbon steel at 1 M hydrochloric acid in the nonappearance and presence of different concentrations of *Eruca sativa* seeds extract at 25 °C.

CPE is expressed by symbol *Υ*^0^, the symbol *j* gives the imaginary root, the angular frequency expressed by *ω*, and the deviation index expressed by symbol *n* (−1 < *n* < 1). The “*n*” values appear to be linked to a non-uniform current distribution because of roughness and possible oxide surface defects. CPE is a perfect capacitor when *n* = 1, however real capacitive behavior is unusual. The divergence from the ideal capacitor is represented by “*n*” values near to 1 (Table 5). A constant phase element (CPE) was employed instead of an ideal capacitor for data fitting; because the “*n*” values obtained were in the range of 0.7–0.8, the value obtained through data fitting was accepted as the capacitance. The chi square value was utilised to assess the same circuit fitting's quality.^115^Table 5 shows that the chi square values obtained (0.000245 to 0.000404) indicate a decent match to the proposed circuit. The Nyquist and Bode plots of the studied systems are shown in Fig. 13 and 14, respectively. For the examined *Eruca sativa* seeds extract analysed, carbon steel demonstrated normal impedance behaviour in 1 M HCl solution, with a significant rise in the diameter for each concentration studied, as shown in Fig. 14. The Nyquist graph of carbon teel in 1 M HCl solution deviated from the desired circular shape due to frequency dispersion.^116^ It's worth noting that altering the concentration of the investigated extract had no effect on the impedance behavior, implying that the corrosion inhibition of carbon steel by inhibitor is comparable. In the absence of *Eruca sativa* seeds extract, the Nyquist plot for carbon steel shows a slightly depressed semicircular pattern, showing that in the 1 M HCl solution, carbon steel corrosion is predominantly regulated by the charge transport mechanism.^117^ The first time constant is related to the capacitive loop of the oxide layer on the carbon steel surface and appears in the mid-frequency range (see Fig. 12). The second time constant appeared in the low frequency range and was attributed to an inductive loop formed by the relaxing of the adsorbed *Eruca sativa* seeds extract on the carbon steel surface or the surface re-dissolution of the carbon steel oxide layer.^118^ By increasing the polarisation resistance, *R*_p_, and decreasing the CPE values, the diameter of the semi-circle grew and the corrosion of carbon steel was retarded when the investigated extract was added to the solution. As a consequence, bigger *R*_p_ values and lower CPE values were linked with the efficacy of the studied extract. This rise got more dramatic as the extract concentration increased, showing extract adsorption on the surface of carbon steel.^119^ The similar tendency was depicted in Bode diagrams. A characteristic with one time constant was noticed in the Bode plots, which corresponded to the capacitance loop. These findings show that in the presence of *Eruca sativa* seeds extract, carbon steel has a higher corrosion resistance. Table 5 lists a variety of impedance metrics, including resistance of charge transfer (*R*_ct_), double layer capacitance (*C*_dl_), *Y* (CPE), *n*, fit goodness (*χ*^2^), and inhibitory efficiency (percent I.E_EIS_). The CPE and its *n* values characterize double-layer capacitors with some holes.^120^ The drop in *Y*^0^ (CPE) values with growing *Eruca sativa* seeds extract concentration was due to a decrease in the local dielectric constant and/or an increase in the thickness of the double layer, implying that these extract molecules hindered carbon steel corrosion through adsorption at the carbon steel/HCl interface. The increase in *R*_ct_ values with increasing *Eruca sativa* seeds extract concentration was always greater than when the extract was not present, implying that it is adsorbed on the carbon steel surface and forms a protective layer. As a mass and charge transfer barrier, this layer is used.^121^ The *R*_ct_ values for *Eruca sativa* seeds extract attained their maximum value of 300 ppm, indicating that the corrosion rate has decreased. On the other hand, as the concentration of extract was increased, the *C*_dl_ values reduced as the inhibitor concentration increased.^122^ The *C*_dl_ value was calculated using the following formula:^123^

21 *C*_dl_ = 1/2π*f*_max_*R*_ct_

**Table 5:** The results of the EIS approach for carbon steel corrosion in 1 M hydrochloric acid at 25 °C with various quantities of *Eruca sativa* seeds extract

Conc., (g L^−1^)	*R*_ct_, Ω cm^−2^	*R*_S_, Ω cm^−2^	*Y* ^0^ × 10^6^, μΩ^−1^ S^*n*^ cm^−2^	*n* × 10^3^	*C*_dl_ × 10^4^, F cm^−2^	*θ*	% I.E_EIS_	*χ* ^2^
Blank	31.2 ± 1.9	1.66 ± 0.015	440.6 ± 7.9	732 ± 0.003	1.860	—	—	0.000301
0.05	273.7 ± 2.44	1.62 ± 0.014	369.6 ± 6.3	743.8 ± 2.47	1.670	0.886	88.6	0.000404
0.10	308.8 ± 2.80	1.80 ± 0.017	214.4 ± 3.9	757.0 ± 2.50	0.897	0.898	89.8	0.000277
0.15	319.2 ± 2.34	1.80 ± 0.018	145.0 ± 2.5	799.0 ± 2.49	0.742	0.902	90.2	0.000245
0.20	362.7 ± 2.69	1.70 ± 0.011	132.2 ± 2.4	814.9 ± 2.27	0.694	0.913	91.3	0.000349
0.25	455.1 ± 3.40	1.20 ± 0.012	138.0 ± 2.2	801.2 ± 2.14	0.599	0.931	93.1	0.000400
0.3 0	578.2 ± 4.60	1.59 ± 0.015	116.5 ± 1.9	796 ± 2.30	0.584	0.946	94.6	0.000272

Using the following equation, the corrosion % I.E_EIS_ was calculated:22
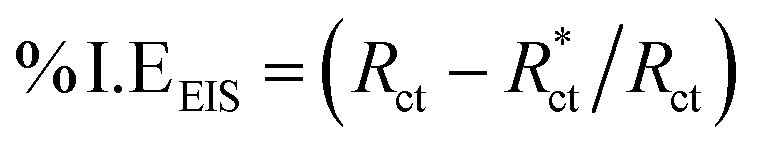


The symbol 
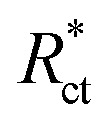
 denotes charge transfer resistance in the absence of the inhibitor, whereas the symbol *R*_ct_ denotes charge transfer resistance in the presence of the extract. The creation of an adsorbed layer from *Eruca sativa* seeds extract on the surface of carbon steel rises the % I.E_EIS_ growths, and the thickness of this layer grows as the concentration of the *Eruca sativa* seeds extract increases.^124^ In addition, the EIS investigation yielded nearly the same inhibitory efficiency as the WL and PDP tests.

### 3.6 Frequency modulation by electrochemistry (EFM)

**Fig. 15:**
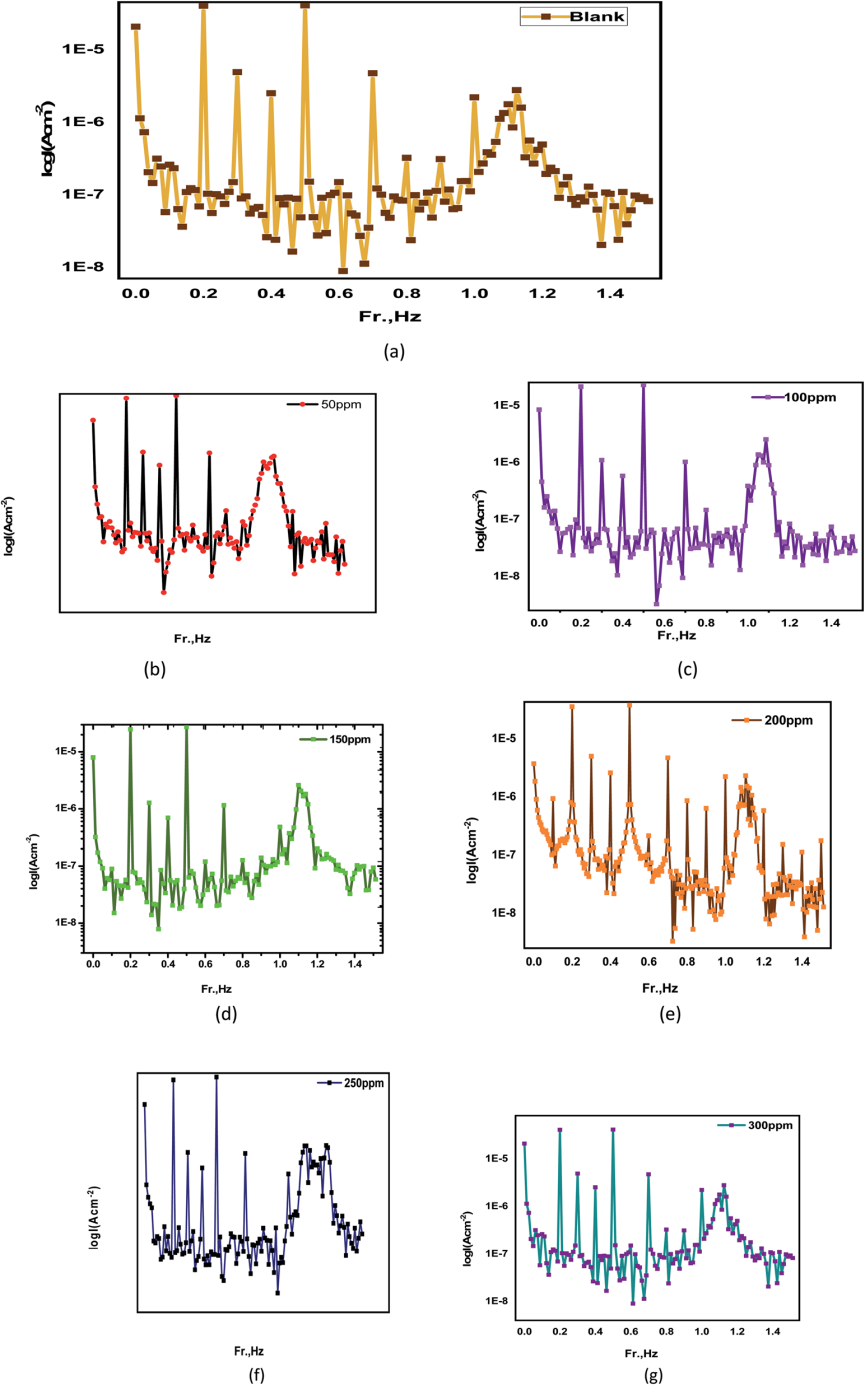
The intermodulation spectrum of carbon steel at 1 M hydrochloric acid in the absence and presence of different concentrations from *Eruca sativa* extract seeds at 25 °C, (a) blank, (b) blank + 0.05 g L^−1^, (c) blank + 0.1 g L^−1^, (d) blank + 0.15 g L^−1^, (e) blank + 0.2 g L^−1^, (f) blank + 0.25, (g) blank + 0.3 g L^−1^.

Because of its advantages, such as quick testing, direct corrosion current values without the need for past knowledge of Tafel constants, and the fact that it is non-destructive, EFM is used in this study.^125^ This method, like EIS, uses a tiny AC signal, but it differs in a few aspects, such as the fact that it can simultaneously apply two sine waves at different frequencies to the cell. Because the current is a non-linear function of potential excitation, the current response in this approach includes frequency components and input frequencies, which are the sum, multiples, and variance of the two input frequencies. The frequencies must be extremely low, and the integer must be multiplied by the base frequency to establish the experiment's length. EFM corrosion properties for a carbon steel electrode immersed in 1MHCl solutions with various concentrations of *Eruca sativa* seeds extract at 25 °C are appeared in Table 6. The inhibitory effectiveness percentage (percent IE) was studied using eqn (23). The initials CF-2 and CF-3 stand for causal factors, which are used to check the rationality of the EFM technique internally.^126^ The systematic values for CF-2 and CF-3 are 2 and 3, respectively. Because the causal variables differ greatly from standard values, noise impairs measurements in this case. Divergence of causality factors from ideal values can also be a factor in the inhibitor's poor performance.^126,127^ This could be because the disruption amplitude is too little or the frequency spectrum resolution is too low. Table 6 shows data on causal factors, with excellent quality measured values. The results of the EFM experiment are represented as an intermodulation spectrum, which is a frequency-dependent spectrum of the current response. Fig. 15 depicts the intermodulation spectrum of a carbon steel electrode in a 1 M hydrochloric acid in the absence and presence of various amounts of *Eruca sativa* seeds extract.^126^Eqn (23) and (24) were used to obtain the percent inhibition and the surface coveraage.

23 IE% = [(*i*_EFM_ − *i*_EFM(Inh._)/*i*_EFM_] × 100

24 *θ* = [(*i*_EFM_ − *i*_EFM(Inh._)/*i*_EFM_]

**Table 6:** At 25 °C, electrochemical parameters for carbon steel in 1 M HCl in the non-existence and presence of various amounts of *Eruca sativa* extract seeds were determined using the EFM technique

Conc.,g L^−1^	*I*_corr_, μA cm^−2^	*β*_a_, mV dec^−1^	−*β*_c_, mV dec^−1^	CF-2	CF-3	*θ*	% IE	mpy
Blank	575.5 ± 5.0	112.2 ± 2.9	166 ± 3	2.03 ± 0.03	2.89 ± 0.02	—	—	60.03
0.05	89.4 ± 2.0	107.0 ± 3.0	170.0 ± 2	2.40 ± 0.05	2.88 ± 0.04	0.844	84.4	50.69
0.1	73.85 ± 2.0	105.60 ± 2.0	178.0 ± 3	2.19 ± 0.06	3.39 ± 0.06	0.872	87.2	35.52
0.15	68.03 ± 0.5	137.00 ± 3.0	156.0 ± 2	2.06 ± 0.07	3.30 ± 0.03	0.881	88.1	32.72
0.20	54.43 ± 0.6	99.85 ± 0.6	160.7 ± 3	2.01 ± 0.04	3.40 ± 0.05	0.905	90.5	24.87
0.25	37.97 ± 0.2	100.5 ± 4.0	154.0 ± 3	1.97 ± 0.02	3.12 ± 0.03	0.934	93.4	18.28
0.30	32.35 ± 0.3	94.23 ± 0.6	180.3 ± 5	2.00 ± 0.02	2.70 ± 0.02	0.943	94.3	15.56

### 3.7 Examining the surface

#### 3.7.1 AFM evaluation

**Fig. 16:**
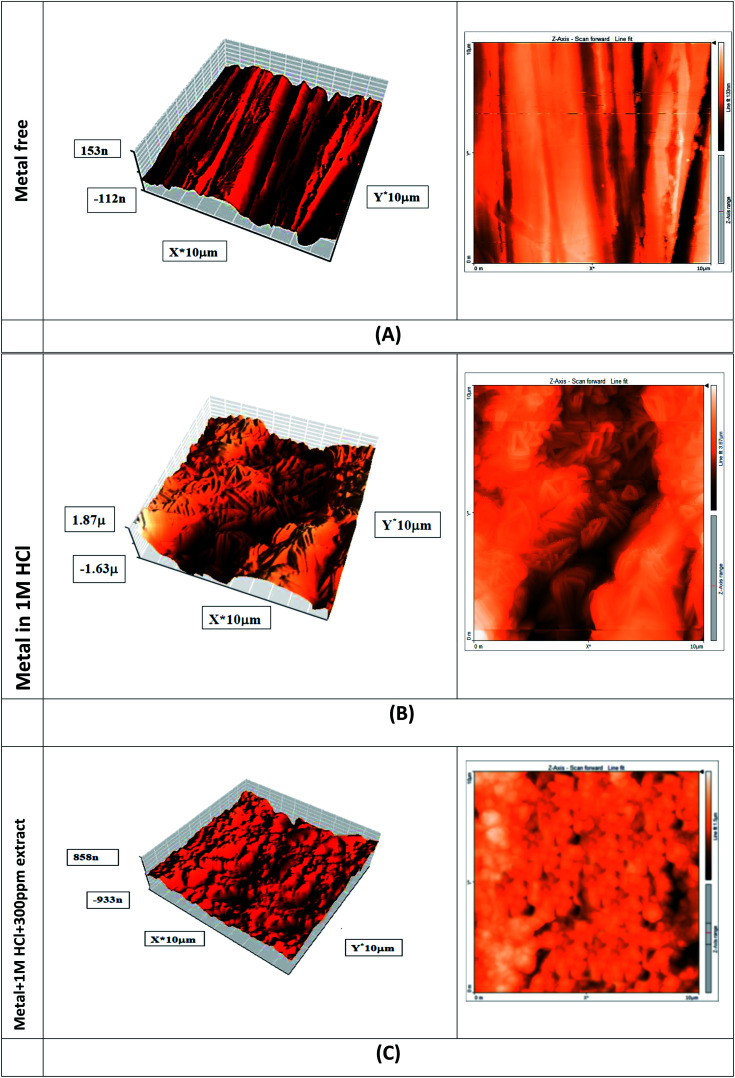
(A) A free atomic force microscope view of the surface of a carbon steel electrode (B) after 24 hours in a 1 M HCl solution, the carbon steel electrode's surface was examined using atomic force microscopy (C) after 24 hours in a 1 M HCl solution containing 300 ppm *Eruca sativa* seeds extract, the surface of the carbon steel electrode was examined using atomic force microscopy.

Atomic force microscopy is one of the extremely accurate techniques that was employed in this study to confirm the previously obtained results (AFM). However, this scan is accompanied by a constant resolution of 1000 times the optical diffraction limit for organizing nanoscale fractions.^128^Table 7 gives the outcomes of this survey, and the root-mean square roughness is expressed by symbol *S*_q_, and this symbol demonstrate the average of the measured height deviancies taken within the assessment length and measured from the mean line, also the average roughness is expressed by symbol *S*_a_, and this illustrate the average deviation of all points' roughness prole from a mean line over the evaluation length, and the greatest peak-to-valley height values is expressed by symbol *P*–*V* (biggest single peak-to-valley height in five adjoining sampling heights). Fig. 16 shows three carbon steel samples. The first sample contains just carbon steel, the second sample contains carbon steel after three hours in a 1 M HCl solution, and the third sample contains carbon steel after three hours in a 1 M HCl solution in the presence of the highest concentration of *Eruca sativa* seeds extract. The small imperfection on the refined carbon steel surface was created by atmospheric corrosion. The corroded alloy surface is illustrated in Fig. 16 in the lack of the *Eruca sativa* seeds extract immersed in 1 M HCl. *S*_q_, *S*_a_, and *P*–*V* carbon steels have surface heights of 590.07, 497.92, and 3932 nm, respectively. The surface of carbon steel dipped in 1.0 M HCl has a rougher surface than the refined alloy surface, according to these data. When carbon steel was immersed in 1 M HCl, the average surface roughness decreased from 497.22 to 145.22 nm with 300 ppm from *Eruca sativa* seeds extract present. Because the extracted *Eruca sativa* seed particles adsorbed onto the carbon steel surface, reducing the interaction between carbon steel and hydrochloric acid, the presence of the highest concentration of *Eruca sativa* seeds extract smoothed the surface and prevented uniform corrosion of the carbon steel surface (Fig. 16).

**Table 7:** In the light of an atomic force microscope, the morphology information of the surface of carbon steel after obsession for day in 1 M HCl solution without and with 300 ppm of *Eruca sativa* seeds extract were investigated

Samples	*S*_q_ (nm)	*S*_a_ (nm)	Maximum peak-to-valley height (nm)
Polished carbon steel	24.792	20.628	137.07
Carbon steel in 1 M HCl	590.07	497.92	3932
Carbon steel + 1 M HCl + 300 ppm extract	175.19	145.22	4145.3

#### 3.7.2 FT-IR analysis

**Fig. 17:**
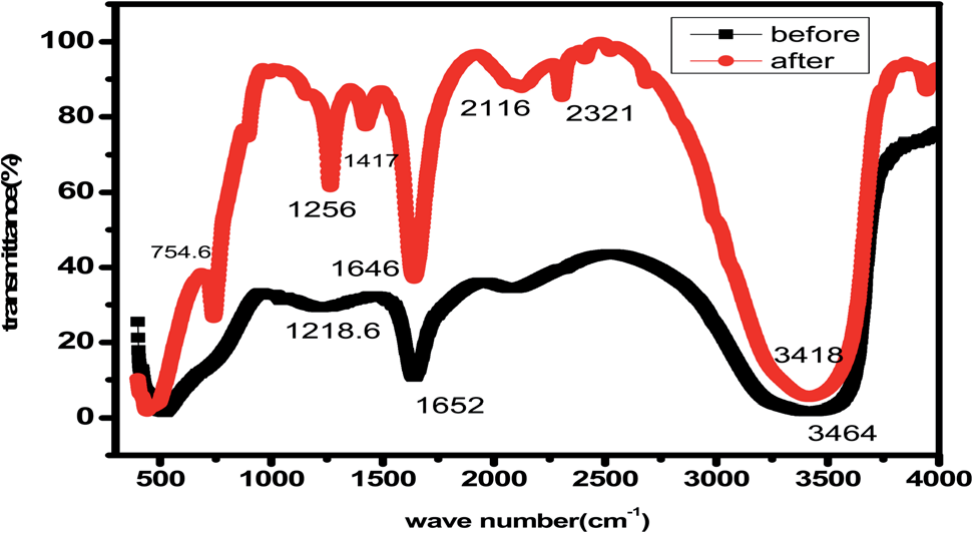
FT-IR spectra of *Eruca sativa* seeds extracted in 1 M HCl before and after adsorption on a carbon steel surface.

Another technique used in our research was FT-IR, which shows the interaction between the surface of carbon steel and the chemical components contained in *Eruca sativa* seeds extract.^129^Fig. 17 illustrates the spectrum of *Eruca sativa* seeds extract before and after soaking a carbon steel coupon for one day. These graphs show that large peaks appeared at 3464, 1652, 1218, and 754.6 cm^−1^ before carbon steel was immersed in 1 M HCl + 300 ppm from *Eruca sativa* seeds extract. Which, respectively, resemble the O–H, aliphatic C–H, C=O, C–O, and Fe stretching vibrations. The wavelength was altered after carbon steel immersion; these changes and shifts demonstrate the interaction between the carbon steel surface and the molecules of *Eruca sativa* seeds extract.^130^ As a result, the functional groups of *Eruca sativa* seeds extract have coordinated with the surface of carbon steel to produce a Fe-complex of extract that promotes inhibition.

#### 3.7.3 X-ray photoelectron spectroscopy (XPS)

**Fig. 18:**
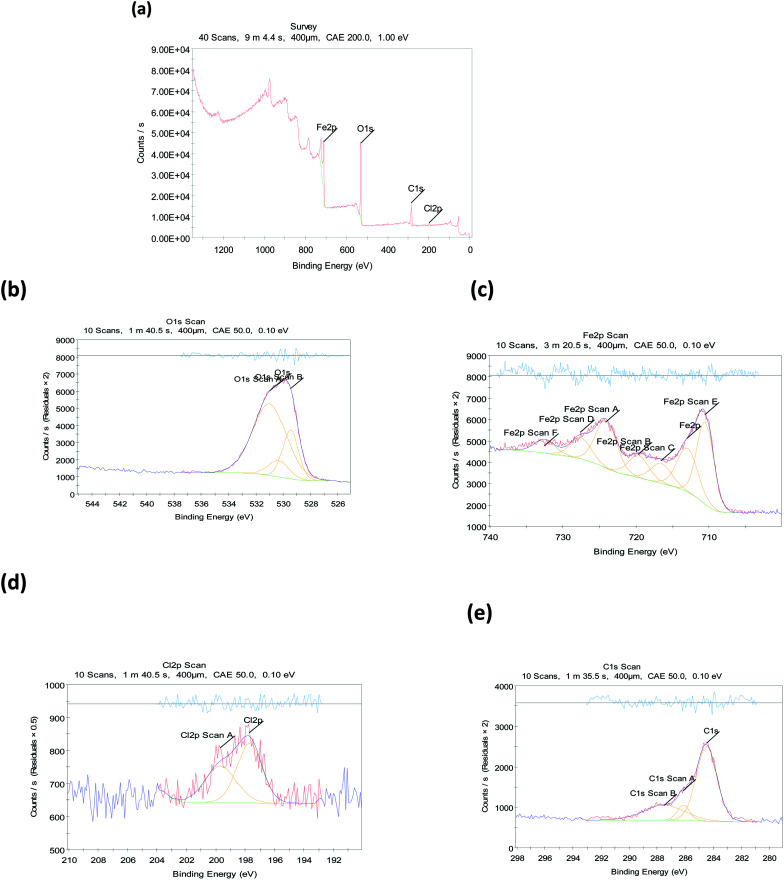
Photoelectric X-rays for carbon steel at 1 M HCl with 300 ppm from *Eruca sativa* seeds extract (a) scanning elements, (b) O 1s, (c) Fe 2p, (d) Cl 2p, and (e) C 1s.

The XPS process was used to analyze the creation of an *Eruca sativa* seeds extract adsorbed layer on a carbon steel surface in a hydrochloric acid media, and the nature of *Eruca sativa* seeds extract adsorption was demonstrated. Fig. 18 shows the XPS decomposition spectra of *Eruca sativa* seeds extract embedded in surface films formed in a solution containing the inhibitor composition. All of these spectra are interpreted using the elemental binding energies provided in the literature and published studies on the interpretation of XPS spectra for superficial films. In the presence of 300 ppm *Eruca sativa* seeds extract, the recorded XPS spectra for carbon steel at 1 M hydrochloric acid comprises Cl 2p, Fe 2p, O 1s, and C 1s components, as shown in Fig. 17(a)–(e). Table 8 gives the binding energies (BE, eV) and identical assignment for each peak component.^131–135^ In 1 M hydrochloric acid with 300 ppm *Eruca sativa* seeds extract, carbon steel C 1s spectra were detected, with three distinct peaks at binding energies of around 284.48, 286.14, and 287.52 eV. Furthermore, O 1s spectra with biding energies of 530.34, 531.01, and 529.47 eV were detected in three different peaks. According to the XPS results, a complex layer containing iron oxide/hydroxide and *Eruca sativa* seeds extract molecules forms on the metal surface. In HCl solutions, these components produce a protective covering that effectively separates the corrosive medium and prevents carbon steel corrosion.

**Table 8:** The binding energies (eV) for the huge core lines found on the carbon steel surface after being treated by *Eruca sativa* seeds extract

Core element	1 M HCl	
	BE, eV	Assignments
C 1s	284.48	C–C, C–H, C–^+^O, C=O
	286.14	
	287.52	
Fe 2p	712.94	Fe_2_O_3_, Fe2p_1/2_ of Fe^3+^, FeOOH, Fe_2_O_3_, FeCl_2_, ferric compounds satellites
	724.1	
	719.64	
	716.55	
	727.57	
	710.51	
	732.48	
Cl 2p	197.71	FeCl_2_
	199.68	
O 1s	530.34	Metal oxide, hydroxide, FeO and Fe_2_O_3_
	531.01	
	529.47	

### 3.8 Chemical parameters at the quantum level

**Fig. 19:**
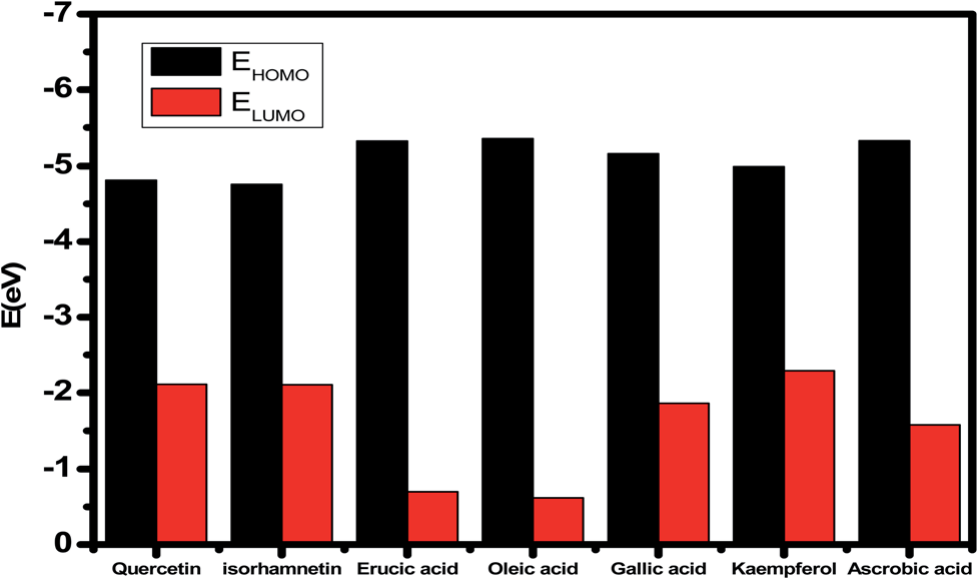
*E*_HOMO_ and *E*_LUMO_ values for several chemicals in *Eruca sativa* seeds extract.

**Fig. 20:**
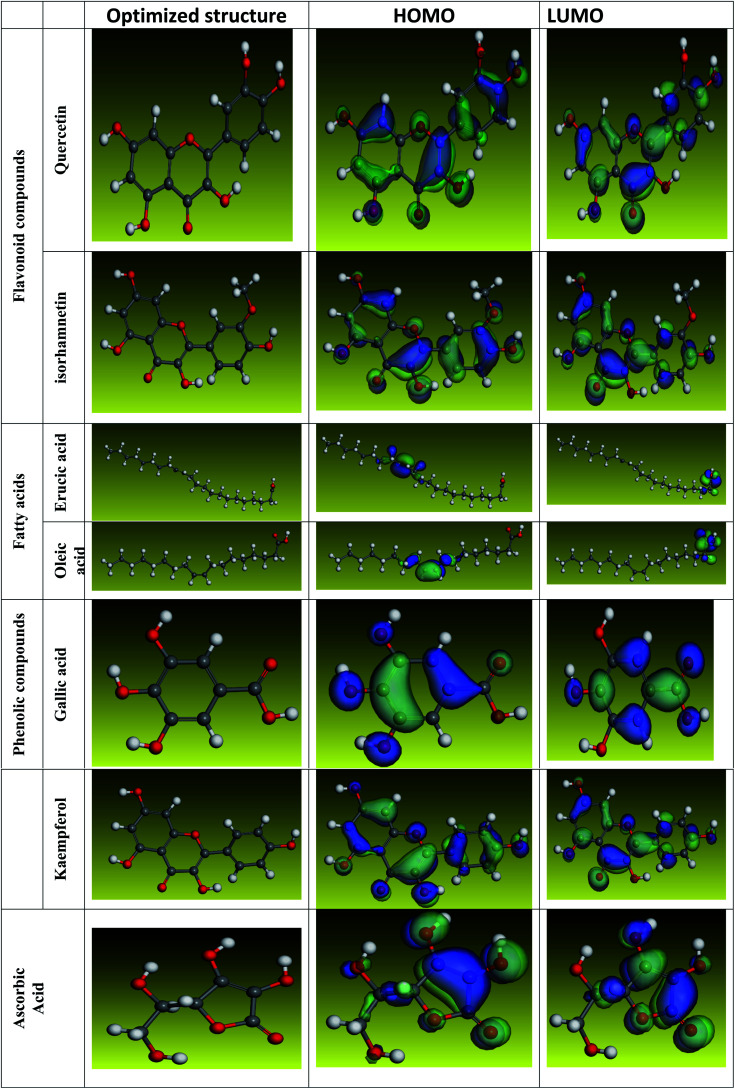
Using the Dmol3/GGA/BOP methodology, the shape of the HOMO and LUMO orbitals, as well as molecular structure optimization, were determined for key components in *Eruca sativa* seeds extract (Neutral).

**Fig. 21:**
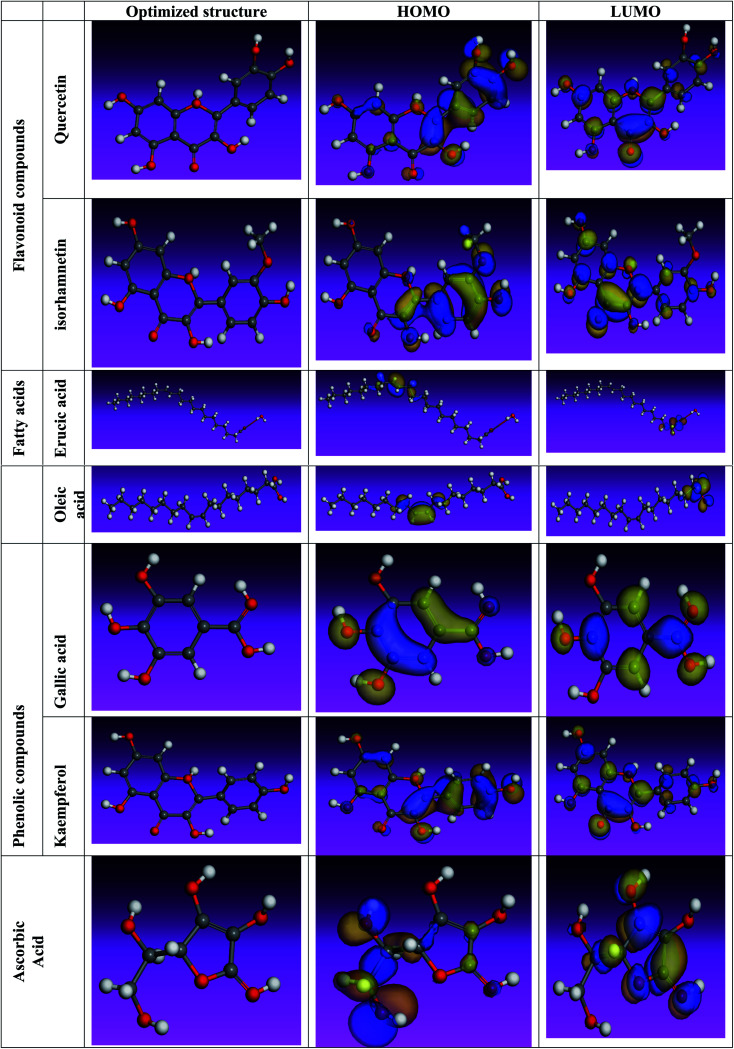
Using the Dmol3/GGA/BOP methodology, the shape of the HOMO and LUMO orbitals, as well as molecular structure optimization, were determined for key components in *Eruca sativa* seeds extract (protonated).

The inhibitory effect of *Eruca sativa* seeds extract, like that of other natural product extracts, can be attributed to phytochemical components adsorption on the carbon steel surface. The varied chemical compositions of its biomass extract make precise experimental assessment of the contributions of distinct elements to the overall inhibitory effect difficult. The utilization of quantum chemical parameters and molecular dynamics simulations in the current study was used to emphasize the unique contributions of various isolated chemicals of *Eruca sativa* seeds extract. The optimum geometric structure of *Eruca sativa* seeds extract orbitals (LUMO and HOMO orbitals) in two cases are shown in Fig. 20 and 21 (neutral and protonated). Higher *E*_HOMO_ values designate that the compound is more capable of delivering electrons to the unoccupied d-orbital on the carbon steel surface, but lesser *E*_LUMO_ values indicate that these molecules can accept electrons.^136^ Corrosion is inhibited by the compound with the most facilities. It's noteworthy to note that the *E*_HOMO_ and *E*_LUMO_ values calculated in Table 9 show that the *Eruca sativa* seeds extract compounds studied can either donate or receive electrons. Table 9 show that erucic acid and oleic acid have the greatest *E*_HOMO_ values, implying that it has the best corrosion inhibition (see also Fig. 19). Another significant metric is the energy gap (Δ*E*), which determines whether or not the molecules have adsorption reactivity on the metal surface (Tables 9 and 10). The adsorption reactivity and the value of Δ*E* have an inverse relationship. Low energy gap values suggest that the eruca seeds extract is an excellent corrosion inhibitor due to the little ionization energy essential to eliminate an electron from the outer shell orbital. The energy gap (Δ*E*) of the compounds of *Eruca sativa* seeds extract in the proton form is lower than that of the neutral form, indicating that the extract has a greater response towards the steel surface in the proton form.^137^ Molecules with small energy gaps are more pliable than those with big gaps. Because they can transmit electrons quickly to their acceptor, soft molecules are more reactive than harder ones. Molecules' nature, stability, and reactivity can all be determined using absolute hardness (*η*) and softness (*σ*) values. Adsorption may only occur in the part of the inhibitor molecule with the highest value for the easiest electron transfer.^138^ The molecules of *Eruca sativa* seeds extract become Lewis base in the corrosion system, whereas carbon steel becomes Lewis acid. For bulk metals whose acid corrosion is induced by soft acids, soft-base inhibitors are the most effective. The dipole moment is the third crucial characteristic, which is employed for structural circulation and rationalization.^139,140^ The dipole moment (*μ*) is another indicator that is frequently used to predict the direction of corrosion inhibition. It is a measure of the polarity of the bond and corresponds to the distribution of electrons in the molecule.^138^ When the inhibitor has a large dipolemoment, it causes a strong interaction (dipole–dipole contact) on the iron surface, resulting in long-lasting adsorption on the carbon steel surface and better inhibition.^139^ Due to the electrostatic attraction between an organic dipole and a charged alloy surface, physical adsorption of the organic retarder onto the metal surface is increased, and hence the bigger dipole moment of the adsorption molecules is favored ability. The shape of the dipole torque changed substantially in protons, implying that *Eruca sativa* extract is physically adsorbed on the steel surface in the form of a proton can molecule, according to a study of dipole moments.^140^ Despite this, there is no apparent association between and inhibition efficacy in the literature.^141^ According to literature, if Δ*N* is smaller than 3.6, the inhibition efficiency improves with the capability of the electronic inhibitor on the metal surface.^142^ An electron's ability to bind inhibitor substances is known as electronegativity (*χ*). The stronger the attraction to accept an electron from a carbon steel surface, the higher the value of *χ*_inh_ (protonated form has a higher value from electronegativity). As a result, higher electronegativity inhibitory particles interact more strongly with the carbon steel surface, increasing inhibition. Compared to previous studies.^143–147^Tables 9 and 10 indicate that the extract is an effective inhibitor. The efficacy of inhibition is increased by widening the area of the molecules of extract, because the interaction area between the molecules of extract and the surface of carbon steel is increased.

**Table 9:** The quantum data for studied *Eruca sativa* seeds extract in the neutral form

Factors	Flavonoid compounds		Fatty acids		Phenolic compounds		Ascorbic acid
	Quercetin	Isorhamnetin	Erucic acid	Oleic acid	Gallic acid	Kaempferol	
−*E*_HOMO_ (eV)	−4.841	−4.758	−5.322	−5.355	−5.161	−4.991	−5.327
−*E*_LUMO_ (eV)	−2.112	−2.107	−0.698	−0.620	−1.862	−2.292	−1.578
Δ*E* (eV)	2.729	2.651	4.624	4.735	3.30	2.70	3.75
*η* (eV)	1.36	1.33	2.312	2.37	1.65	1.350	1.87
*σ* (eV)^−1^	0.735	0.752	0.433	0.421	0.6	0.740	0.535
Pi (eV)	−3.48	−3.43	−3.01	−2.99	−3.511	−3.64	−3.45
*χ* (eV)	3.48	3.43	3.01	2.99	3.511	3.64	3.45
Dipole moment (debyes)	8.7323	9.7757	2.2968	1.8590	3.4497	8.5829	9.4327
Molecular area (Å^2^)	314.84	305.75	481.311885	397.523598	178.312238	277.561300	181.37
Δ*N*_max_ (e)	0.294	0.319	0.274	0.272	0.233	0.237	0.221

**Table 10:** The quantum data for studied *Eruca sativa* seeds extract in the protonated form

Factors	Flavonoid compounds		Fatty acids		Phenolic compounds		Ascorbic acid
	Quercetin	isorhamnetin	Erucic acid	Oleic acid	Gallic acid	Kaempferol	
−*E*_HOMO_ (eV)	−5.559	−5.468	−5.370	−5.385	−5.811	−5.546	−6.369
−*E*_LUMO_ (eV)	−2.967	−3.127	−3.372	−2.890	−3.319	−2.944	−3.034
Δ*E* (eV)	2.592	2.341	1.998	2.495	2.492	2.602	3.335
*η* (eV)	1.296	1.170	0.999	1.250	1.246	1.301	1.668
*σ* (eV)^−1^	0.771	0.854	1.001	0.800	0.803	0.885	0.599
Pi (eV)	−4.263	−4.298	−4.371	−4.138	−4.565	−4.245	−4.702
*χ* (eV)	4.263	4.298	4.371	4.138	4.565	4.245	4.702
Dipole moment (debyes)	9.8603	10.4048	54.1264	45.7021	7.5240	9.7414	11.0437
Molecular area (Å^2^)	290.60	307.79	508.73	398.89	179.29	284.28	183.30
Δ*N*_max_ (e)	0.008	−0.008	−0.046	0.06	−0.11	0.013	−0.252

#### 3.8.1 Fukui indices and Mulliken charges

**Fig. 22:**
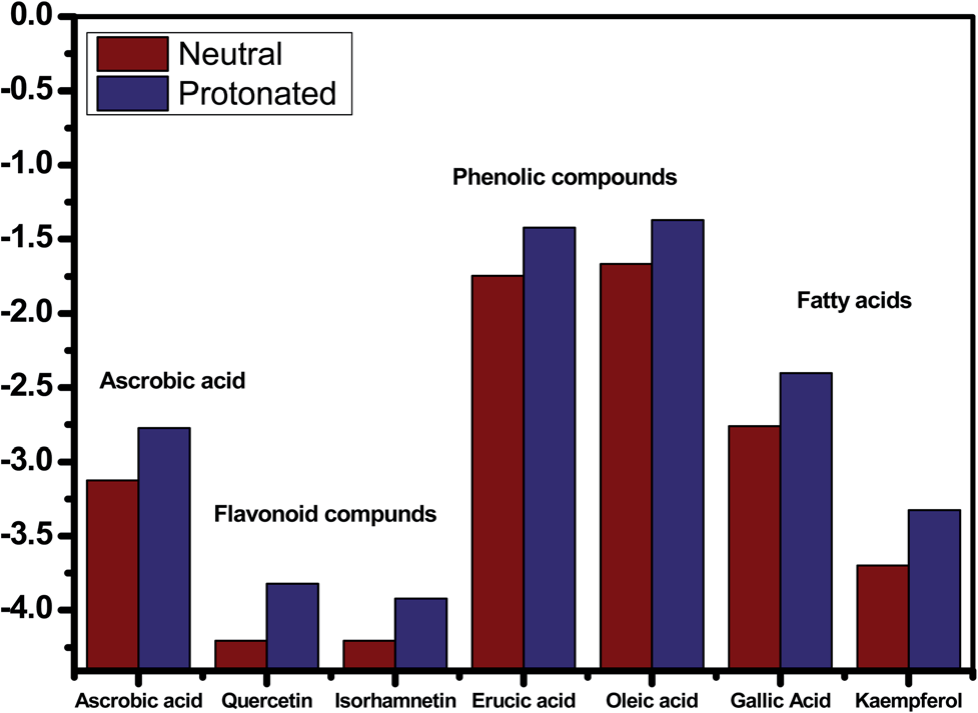
TNC of *Eruca sativa* seeds extract protonated and non-protonated molecules.

**Fig. 23:**
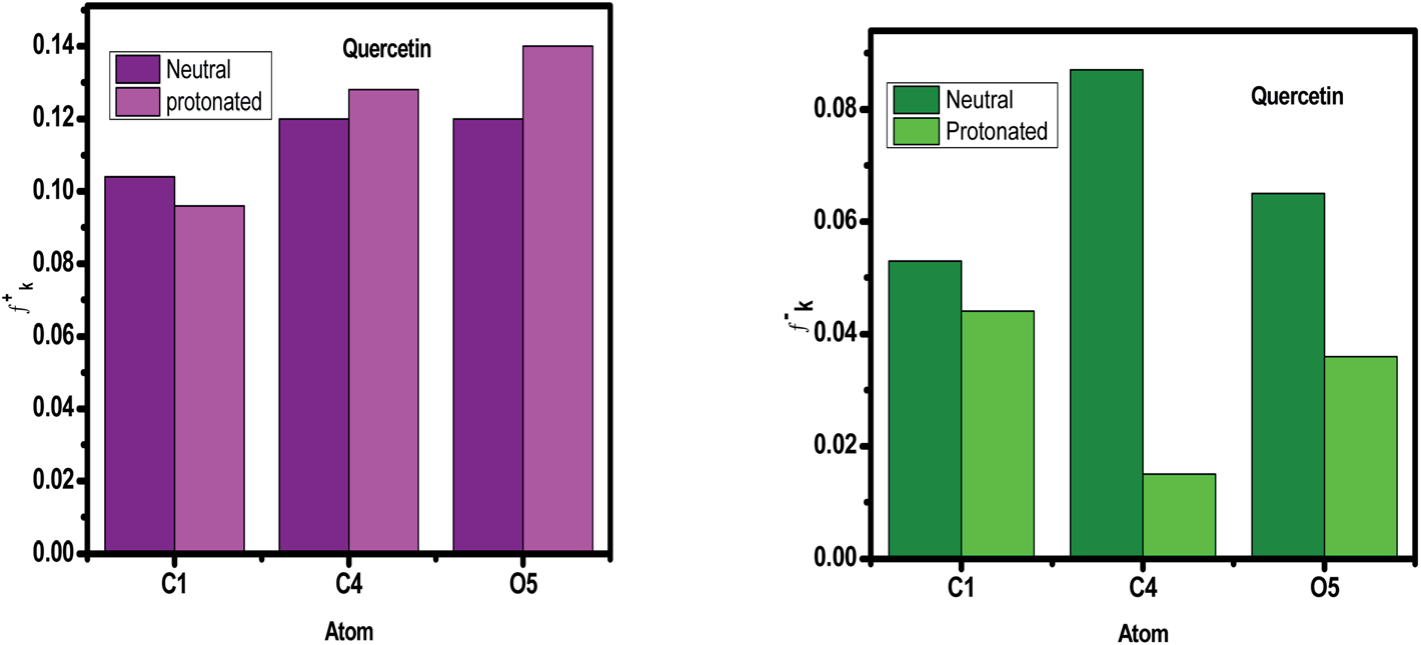
Graphical representation of the Fukui indices of Quercetin for the more reactive atoms in the un protonated and protonated form.

Because we need to know where the donor and acceptor of the molecule's active centers are, we'll utilize Mulliken atomic charges and Fukui indices to figure it out. Tables S3–S9† summarize the results of the various electrophilic and nucleophilic positions from neutral and protonated particles from compounds of *Eruca sativa* seeds extract. The Mulliken distribution values are both negative and positive in the tables, showing that the inhibitor compounds have active sites (donor–acceptor) that improve these species' sensitivity to iron atoms. The direction of all the values of the proton molecule positions changed considerably, showing an increase in the proton inhibitors' molecular interaction, and so this observation illustrates the neutral molecules' receptive nature. Based on the results in Tables S3–S9† and displayed in Fig. 22, comparable behavior was found in terms of total negative charge (TNC), with the TNC increasing after the inhibitor was protonated.^148^ This shows that protonated particles' structural interaction is rising. According to TNC, phenolic compounds have a greater electron donor property than the other chemicals. This increases their ability to adsorb on metal surfaces. Organic compounds with one or more heteroatoms can protonate at these locations in acidic environments (HCl), resulting in the creation of positively charged molecules. These interact with the widely dispersed anions (Cl^−^) on the metal surface. In this context, we'll use this idea to investigate the influence of protonation on inhibitory compounds' local centers. Fig. 22 and Tables S3–S9† illustrates the atoms of the studied compounds with bigger values of *f*_k_^+^ and *f*_k_^−^ in the protonated and non-protonated forms, as another markers (Fukui's index (FI)). For both electrophilic and nucleophilic assault systems, the overall analysis of this picture reveals that each molecule has different atoms. The preferred site for nucleophilic assault is the atom in the molecule with the highest Fukui function (*f*_k_^+^), which is linked to the LUMO and degrees the reactivity to donor reagent. Because it is related to the HOMO and gives the reactivity toward an acceptor reagent, the atom in the molecule with the highest value of the Fukui function (*f*_k_^−^) is chosen for electrophilic assault. The values of *f*^+^ for atoms increased after protonation because these atoms now have the ability to take electrons, but the values of *f*^−^ for atoms decreased in the protonated state due to a loss in donor property due to these centers are blocked by protons H^+^ (Fig. 23, S10–S15†). Also we notice that some atoms in the same compound have higher *f*^–^ values, and other atoms have *f*^+^ higher values, so the same compound can accept and donate electrons in two case (neutral and protonated form).

#### 3.8.2 Electrostatic potential of molecules (MESP)

**Fig. 24:**
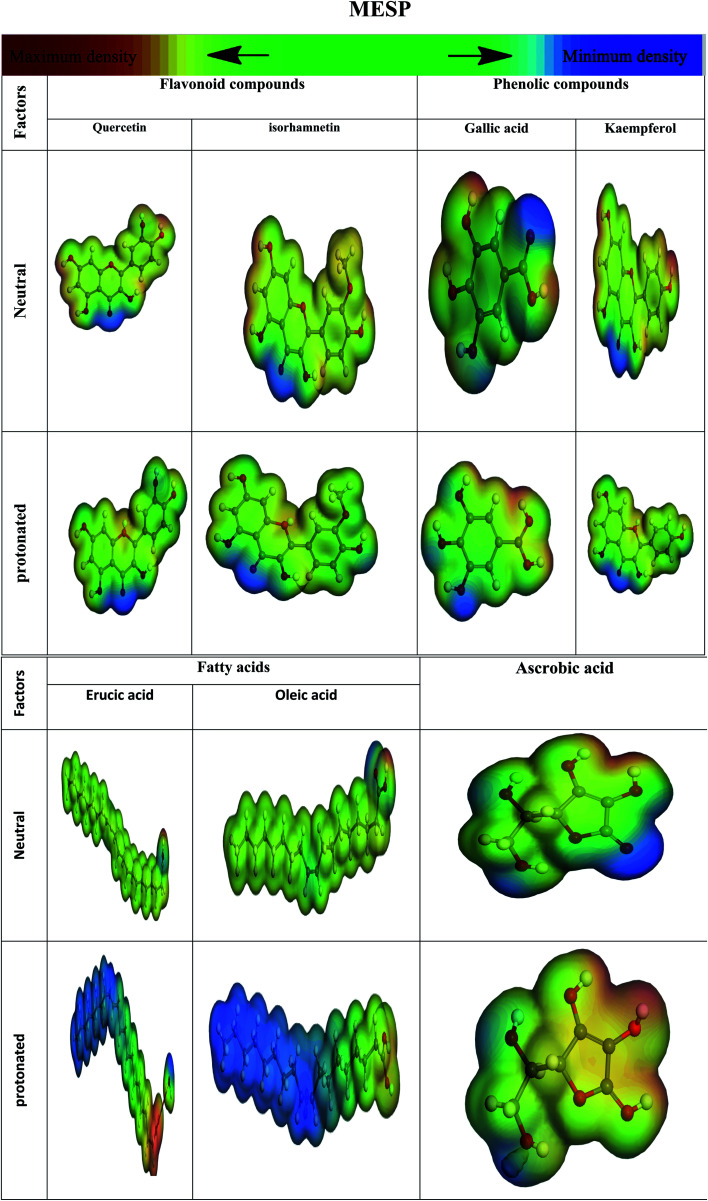
Images illustrate the protonated and un-protonated molecule electrostatic potentials, along with the inhibitor's electrostatic contour.

The molecular electrostatic potential (MESP) has long been used to show where a chemical reaction is occurring. At various points on the electron density's surfaces, electrostatic potential is colored differently. The electrically active and electrophilic zone is highlighted in red, and the electrostatic potential is primarily negative. The color blue indicates regions with the highest positive electrostatic potential (nucleophilic region), whereas green indicates regions with no likelihood.^149^ Hetero-atoms and double conjugate bonds are mostly found in electron-dense areas. Oxygen, nitrogen, and chloride groups indicate negative zones that favor electrophilic assaults. Blue-colored hydrogen atoms favor nucleophilic assaults (positive sign) Fig. 24.

### 3.9 Molecular dynamics simulation

**Fig. 25:**
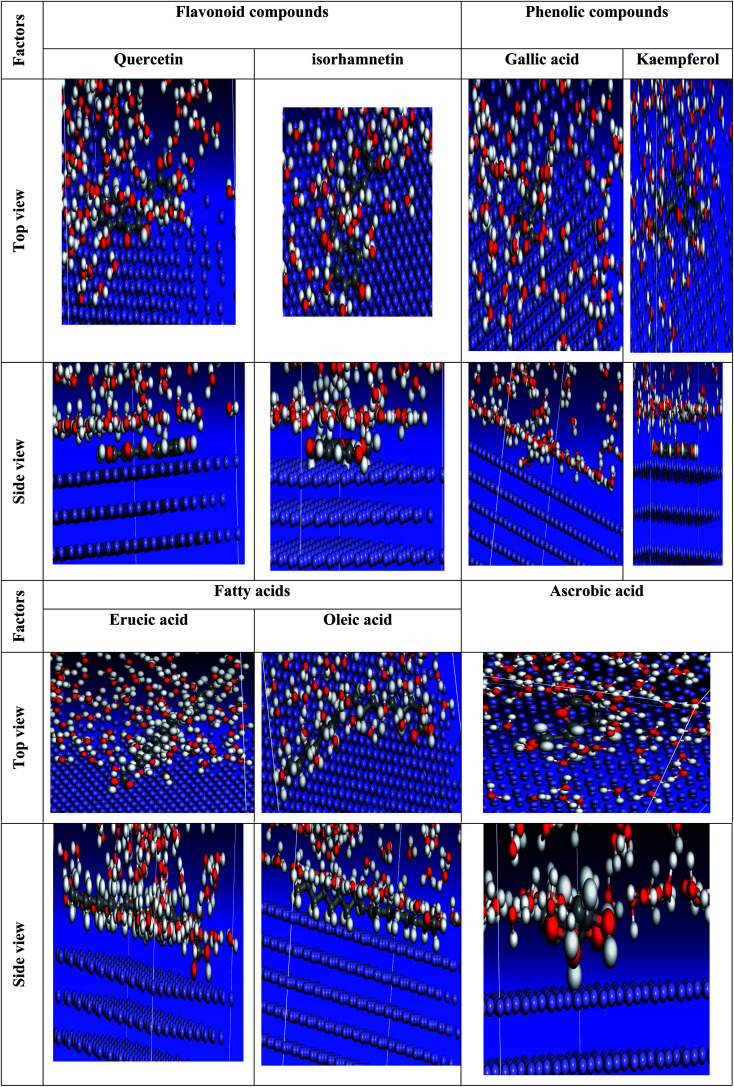
The most suited formation for adsorption of *Eruca sativa* seeds extract on carbo iron (1 1 0) substrate as determined by the adsorption locator module (neutral form).

**Fig. 26:**
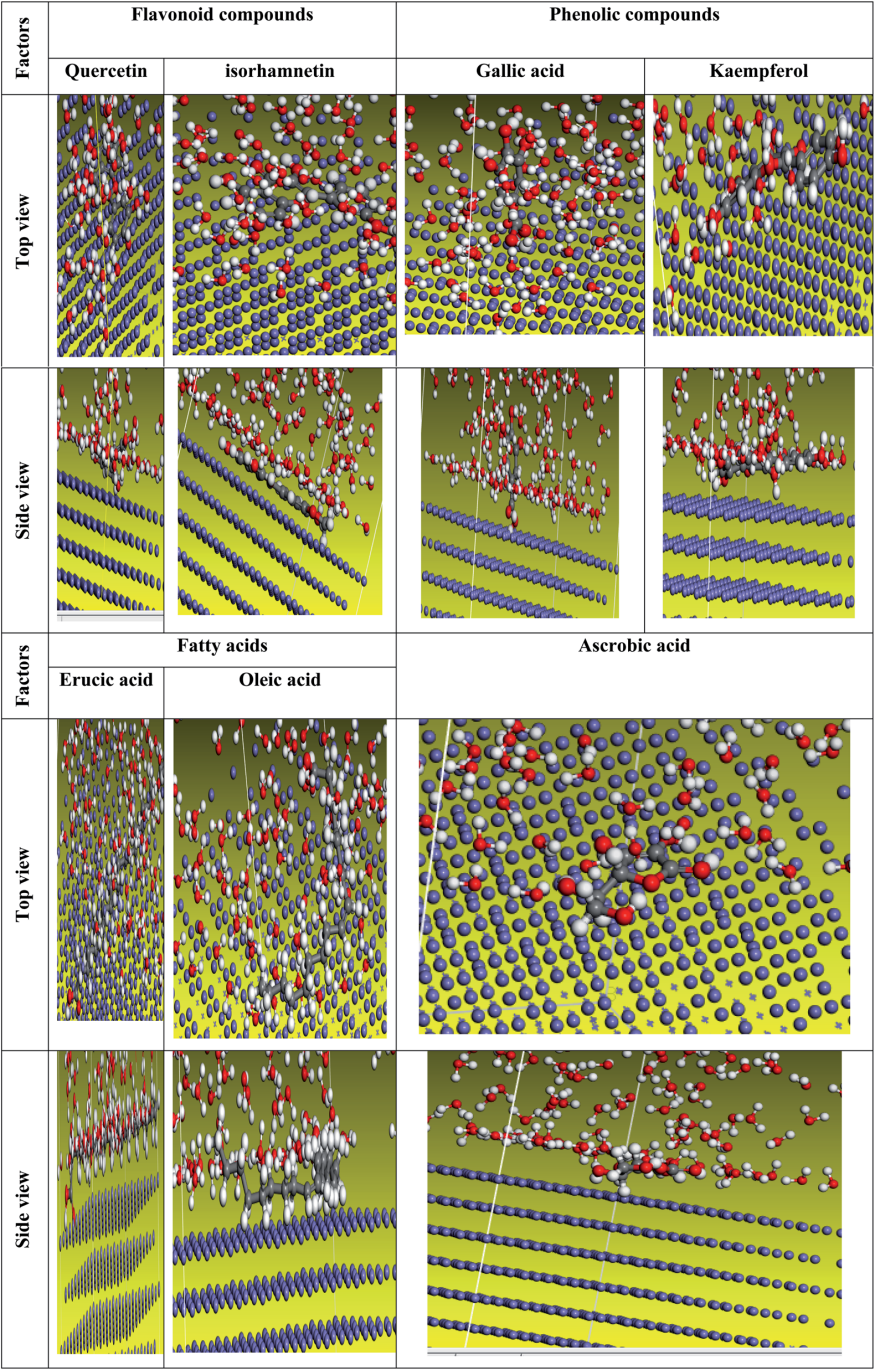
The most suited formation for adsorption of *Eruca sativa* seeds extract on carbo iron (1 1 0) substrate as determined by the adsorption locator module (protonated form).

**Fig. 27:**
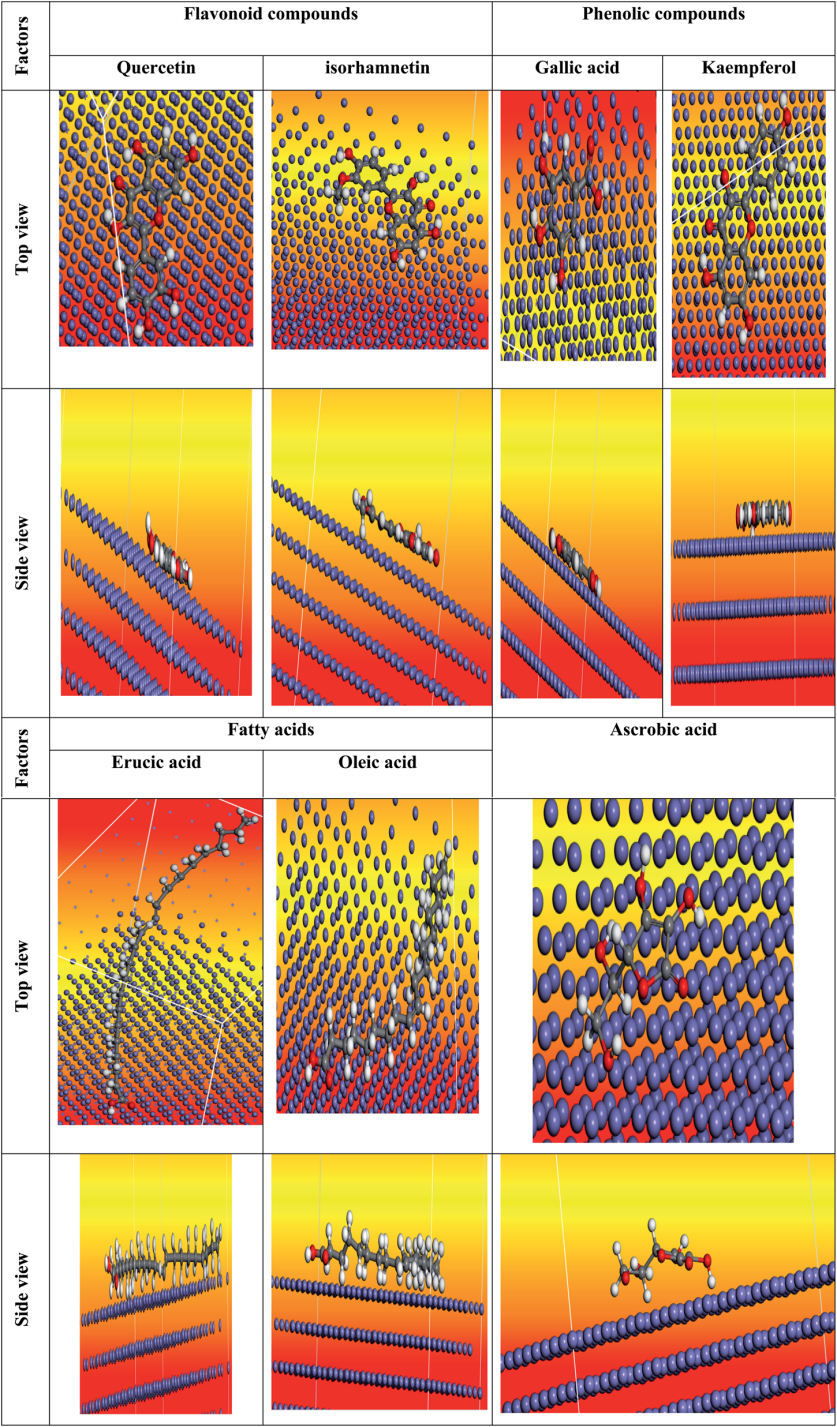
The most suited formation for adsorption of *Eruca sativa* seeds extract on carbo iron (1 1 0) substrate as determined by the adsorption locator module (in the vacuum).

We carefully determined the proper prearrangement for inhibitor molecule uptake on the Fe (1 1 0) substrate in this study, which was accomplished by the adsorption locator illustrated in Fig. 25. Table 11 shows the Monte Carlo simulation outputs for total energy, solid adsorption, and deformation energies. Total energy (kcal mol^−1^) calculation for the substrate – the adsorbent as a combination of solid adsorption energy energies of the molecules and deformation energy. In our study, the surface energy of carbon steel (110) was set to zero. The energy liberation that occurs when calming substances are adsorbed for relaxation is known as adsorption of energy (kcal mol^−1^) (the compounds of extract studied are adsorbed on the surface of the substrate). The adsorption energy of the adsorbate molecules is a combination of deformation and solid adsorption energy. Prior to the geometry optimization stage, disturbed adsorption molecules are adsorbed on the substrate and the constant adsorption energy (kcal mol^−1^) is released. When adsorbed adsorbent components are positioned on the surface of the substrate, deformation energy (kcal mol^−1^) is created.^150^ dEads/dNi is the amount of energy gained when one of the adsorbate molecules is separated from the adsorbent substrate formation (kcal mol^−1^). Table 11 shows the adsorption energies of the *Eruca sativa* seeds extract. As a result, as demonstrated by theoretical and experimental research, the molecules of *Eruca sativa* seeds extract are very likely adsorbed on the surface of carbon steel, forming adsorbed layers, providing protection against hydrochloric acid solution corrosion on the carbon steel surface. According to Tables 11 and 12 and prior investigations,^151–153^ in solution, the examined components performed well (high adsorption energy). Close examination indicates that the local molecule's structural structure is parallel to the surface.^154,155^ This adsorption pattern will be used by the components of the retarding particles to maximize the target metal's contact area or surface covering properties (*i.e.* carbon steel). The expanded metal/inhibitor contact area will decrease substrate access rather than causing undesired chloride anion assaults.^154,155^ In the event of a charged proton component, the electron transfer is expected to be in reverse, from the metallic surface to the components of the extract, causing the reaction to revert. As a result, as revealed in both experimental and theoretical research Table 12, our extract molecules securely adsorb on the carbon steel surface, forming strong adsorbent layers that protect the carbon steel surface from hydrochloric solution acid corrosion. We explored molecule adsorption in both an acidic (neutral and proton) and a vacuum environment (Fig. 25–27). The adsorption energies climb above the vacuum value in the presence of an aqueous solution, implying that particle adsorption on the carbon steel surface is more efficient (see Tables 11 and 13 for more information.).

**Table 11:** To adsorb iron inhibitors, data and specifications are obtained by simulating Monte Carlo (neutral)

Factors	Flavonoid compounds		Fatty acids		Phenolic compounds		Ascorbic acid
	Quercetin	isorhamnetin	Erucic acid	Oleic acid	Gallic acid	Kaempferol	
Total energy (kcal mol^−1^)	−3282.2	−3318.9	−3378.4	−3308.9	−3222.0	−3305.5	−3111.4
Adsorption energy (kcal mol^−1^)	−3240.9	−3294.9	−3296.7	−3242.1	−3197.0	−3263.0	−3130.1
Rigid adsorption energy (kcal mol^−1^)	−3401.7	−3454.8	−3463.4	−3408.6	−3356.0	−3422.3	−3292.6
Deformation energy (kcal mol^−1^)	160.7	159.9	166.71	166.45	159.0	159.3	162.49
dEad/dNi (kcal mol^−1^)	−207.23	−218.6	−210.09	−191.6	−93.76	−199.1	14.09

**Table 12:** The adsorption of iron inhibitors, Monte Carlo simulations are used to obtain data and requirements (protonated form)

Factors	Flavonoid compounds		Fatty acids		Phenolic compounds		Ascorbic acid
	Quercetin	isorhamnetin	Erucic acid	Oleic acid	Gallic acid	Kaempferol	
Total energy (kcal mol^−1^)	−3196.9	−3220.2	−3378.4	−3340.1	−3226.7	−3226.7	−3115.2
Adsorption energy (kcal mol^−1^)	−3153.6	−3195.6	−3296.7	−3268.7	−3192.3	−3182.1	−3146.9
Rigid adsorption energy (kcal mol^−1^)	−3303.3	−3354.9	−3463.4	−3334.9	−3352.6	−3343.3	−3321.7
Deformation energy (kcal mol^−1^)	149.7	159.3	166.7	166.26	160.3	161.3	174.8
dEad/dNi (kcal mol^−1^)	−43.86	−218.7	−210.1	−174.6	−29.3	−96.3	−117.15

**Table 13:** The adsorption of iron inhibitors, Monte Carlo simulations are used to obtain data and requirements (vacuum form)

Factors	Flavonoid compounds		Fatty acids		Phenolic compounds		Ascorbic acid
	Quercetin	Isorhamnetin	Erucic acid	Oleic acid	Gallic acid	Kaempferol	
Total energy (kcal mol^−1^)	−200.64	−199.5	251.04	−247.05	−109.07	−197.4	−57.99
Adsorption energy (kcal mol^−1^)	−159.4	−176.2	−27.28	−179.7	−84.03	−154.8	−80.61
Rigid adsorption energy (kcal mol^−1^)	−152.5	−166.4	−211.02	−186.3	−84.4	−147.92	−82.36
Deformation energy (kcal mol^−1^)	−6.87	−9.82	--60.25	6.6	0.38	−8.93	1.75
dEad/dNi (kcal mol^−1^)	−159.4	−176.2	−271.28	−179.7	−84.03	−154.8	−80.6

### 3.10 Corrosion inhibition mechanism

WL, PDP, EIS, EFM, and various surface analysis techniques, in addition theoretical studies were utilized to validate the adsorption of *Eruca sativa* seeds extract on the carbon steel surface to build a layer that shields the metal's surface from the corrosive medium (1 molar of hydrochloric acid). The inhibitor's chemical makeup and molecular size, as well as other related factors such as the reactivity of substituents of the benzene ring, electron density, functional groups, alloy charge, and the nature of the corrosive middle, determine the degree of inhibition and interaction between the extract molecules and the alloy surface. Two types of functional groups have been discovered as having the potential to influence inhibitor molecule reactivity. Electron withdrawing groups were discovered in the benzene ring, which pulls electrons away from the cyclic π-system, lowering the electron density (*i.e.* the ring is electron poor and less reactive), thus naming the disrupted groups as carbonyl groups and carboxylic acid.^156^ Aromatic rings bound to electron-donating groups (as hydroxyl group), on the other hand, provide electrons to the reaction center (*i.e.*, the ring is electron-rich and more reactive) and thus settle an electron deficiency in the other binding material (*e.g.*, iron). According to the literature, the inhibitor molecule interacts with the carbon steel surface mostly by physical and/or chemical adsorption.^157,158^ The subsequent mechanism for steel corrosion in HCl solution was projected previously as a series of phases.^159,160^

Reactions at anodic sites:

I Fe + Cl^−^ → (FeCl^−^)_ad_

II (FeCl^−^)_ads_ → (FeCl)_ads_ + e^−^

III (FeCl)_ads_ → FeCl^+^ + e^−^

IV FeCl^+^ → Fe^2+^ + Cl^−^

Reactions at the cathodic location

V Fe + H^+^ → (FeH^+^)_ads_

VI (FeH^+^)_ads_ + e^−^ → (FeH)_ads_

VII (FeH)_ads_ + H^+^ + e^−^ → Fe + H_2_

The mechanism outlined overhead illustrates that the anodic dissolution of Fe and the cathodic hydrogen evolution govern the rate of corrosion of Fe in HCl. The inhibitory mechanism in the current study, one or more of the following explanations can explain the hetero atom adsorption on the carbon steel surface in 1 M HCl solution. The molecules of the extract can be adsorbed on the surface of iron in two ways: at first, through unshared pair electron interaction in the oxygen atoms, or/and the benzene ring π-electron charge-transfer to the vacant d-orbital of iron, which has low energy, which occurs in the anodic area, and second, through a coordinate chemical bond created between the molecules of the *Eruca sativa* seeds extract and the surface of iron. The second method is physical adsorption, which occurs when the molecules of *Eruca sativa* seeds extract are protonated, and then electrostatic attraction with chloride anions occurs, causing (FeCl^−^)_ads_ to form bridges between *Eruca sativa* seeds extract molecules and alloy, and then the extract is adsorbed on the cathodic zone to compete hydrogen ions (stride V), causing cathode polarization to grow.^161^ A mixture of π-electron and electrostatic interactions^162^ is another possibility. A number of experimental discoveries and theoretical computations strongly support the second alternative. The inhibitor molecules are first rapidly protonated in HCl solution, yielding positively charged inhibitor species. As a result, the surface charge of carbon steel at zero, also known as the zero charge potential, has to be calculated (ZCP). It can be calculated using the value of (*E*_corr_ − *E*_q_ = 0). When the value (*E*_corr_ − *E*_q_ = 0) is greater than zero, the surface charge is positive.^163^ As determined earlier,^164^ In HCl solution, the ZCP of iron is *E*_q_ = −530 mV *vs.* SCE. Returning to Table 4, the maximum *E*_corr_ values for extract components *vs.* SCE is −481.5 mV achieved at 300 ppm. As a result, the calculated value of Fe-ZCP is 48.57 mV, indicating a positively charged carbon steel surface. Between the protonated charged inhibitor and the positively charged steel surface, electrostatic repulsion is expected. The electrostatic attraction between the anionic species and the protonated charged inhibitor on the electrolyte/metal interface should cover the surface of the carbon steel with negatively charged chloride ions (Cl^−^) in the HCl solution. Positively charged extract molecules bind to the carbon steel surface as a result of the chloride-bridge, generating the first adsorption layer. This method achieves physical adsorption of these molecules, resulting in the formation of a thin protective layer on the entire alloy surface, significantly lowering the corrosion rate of carbon steel. Second, the computed value of Δ*G*^0^_ads_ for *Eruca sativa* seeds molecules is less than −20 kJ mol^−1^ (see Table 3), indicating an electrostatic contact amid the charged molecules and the negatively charged alloy surface, *i.e.* physisorption.

## 4. Studies on comparative efficiency and economics

Because of their very poisonous nature and negative effects on the environment and human health, the use of inhibitors like chromates has been restricted, and green corrosion inhibitors such as leaves, roots, shells, and seeds of fruits have gotten a lot of attention as a potential substitute. Green inhibitors had a high inhibitory efficiency due to the presence of organic compounds with numerous heteroatoms and active functional groups. Based on the weight loss approach of utilizing 0.3 g L^−1^*Eruca sativa* seeds extract, 94.8 percent efficiency was obtained in the current study. Table S1† shows that this efficiency is higher than that of the majority of green inhibitors.^165–174^ These studies show that extracting all inhibitors with lower efficiency than *Eruca sativa* seeds extract is more expensive than extracting *Eruca sativa* seeds extract. It is observed that making 1 kg of *Eruca sativa* seeds extract costs around $5–10, whereas most green inhibitor extraction processes cost more over $50 per kg. High-performance inhibitors, on the other hand, such as polysaccharide^175^ and *Ceratonia siliqua* L seed oil,^176^ are more expensive and thus not affordable for large scales such as industrial applications due to the high preparation cost. *Eruca sativa* seeds extract is a new research because the other researchers used *Eruca sativa* leaves not seeds. Found different in chemical components between leaves and seeds extract. In case of seeds extract found more phenolic components.^57–60^ When comparing the inhibition efficiency of seeds *vs. Eruca sativa* leaves extract, we discovered that the inhibition efficiency of seeds extract is greater than the inhibition efficiency of *Eruca sativa* leaves extract,^177^ more information can be found in ESI.†

## 5. Conclusion

The *Eruca sativa* seeds extract showed good inhibitory efficiency for carbon steel corrosion in 1 M hydrochloric acid. According to PDP, *Eruca sativa* seeds extract functions as a mixed-type inhibitor. By raising the dose of *Eruca sativa* seeds extract and decreasing the temperature, the inhibitor's efficiency is improved. Furthermore, when the concentrations of the compounds we tested rise, double layer capacitances drop. The adsorption of the extracted molecules followed the Langmuir and Henry isotherms, and the adsorption was physical. The extract particles' activation energy considers more values than the blank activation energy, resulting in a greater extract dose. According to surface analysis, an extract layer formed on the carbon steel surface as the film stopped metal from dissolving in the hydrochloric acid solution (XPS, FTIR, and AFM). All of the experimental data obtained using the WL approach and electrochemical techniques (PDP, EIS, and EFM) were in agreement.

## Conflicts of interest

There are no conflicts to declare.

## Supplementary Material

RA-012-D2RA01296K-s001
