# Supplementary material for: Green inhibitor of carbon steel corrosion in 1 M hydrochloric acid: Eruca sativa seed extract (experimental and theoretical studies)
Source: RSC Adv. 2022 Mar 23;12(15):8953–86. doi: 10.1039/d2ra01296k (PMC8985086; doi:10.1039/d2ra01296k)
Supplement: RA-012-D2RA01296K-s001 [file RA-012-D2RA01296K-s001.pdf]

Electronic Supplementary Material (ESI) for RSC Advances.
This journal is © The Royal Society of Chemistry 2022

Supporting Information (SI)

Green inhibitor of Carbon steel corrosion in 1 M hydrochloric acid: Eruca sativa seeds extract (Experimental and Theoretical studies

Weight loss results:

Table S1

Weight loss measurements of carbon steel in 1MHCl solution containing various concentrations of eruca sativa seeds extract after immersion time for 90min at 25, 35 °C,

Conc., g/L	25 ^{0}C				35 ^{0}C			
	Δm $mgcm^{-2}$	θ	%IE	C.R. mg $cm^{-2}min^{-1}$	Δw $mgcm^{-2}$	θ	%IE	C.R. mg $cm^{-2}min^{-1}$
**Blank**	22.81±0.004			0.3802	25.30±0.003			0.4209
**0.05**	3.43±0.002	0.850	85.0	0.0571	7.83±0.001	0.690	69.0	0.13052
**0.10**	2.98±0.001	0.870	87.0	0.0496	7.66±0.002	0.697	69.7	0.12760
**0.15**	2.28±0.003	0.900	90.0	0.03788	6.74±0.001	0.733	73.3	0.11227
**0.20**	1.84±0.001	0.919	91.9	0.03061	5.05±0.003	0.800	80.0	0.08421
**0.25**	1.39±0.001`	0.939	93.9	0.02321	4.61±0.001	0.817	81.7	0.07684
**0.30**	1.184±0.002	0.948	94.8	0.01973	3.34±0.002	0.868	86.8	0.05570

Table S2

**Conc., Ppm**	**45 ^{0}C**				**55 ^{0}C**			
	**Δw mgcm^{-2}**	**θ**	**%IE**	**C.R. mg cm^{-2}min^{-1}**	**Δw mgcm^{-2}**	**θ**	**%IE**	**C.R. mg cm^{-2}min^{-1}**
**Blank**	26.39±0.002			0.4365	27.43±0.005			0.4630
**0.05**	13.49±0.001	0.447	44.7	0.2248	17.13±0.003	0.383	38.3	0.2856
**0.10**	13.34±0.003	0.453	45.3	0.2223	16.26±0.002	0.415	41.5	0.2710
**0.15**	12.27±0.002	0.497	49.7	0.2045	15.20±0.002	0.453	45.3	0.2533
**0.20**	10.76±0.004	0.559	55.9	0.1794	12.46±0.001	0.552	55.2	0.2076
**0.25**	9.45±0.002	0.613	61.3	0.1575	10.97±0.001	0.605	60.5	0.1828
**0.30**	8.48±0.003	0.652	65.2	0.1413	10.11±0.002	0.636	63.6	0.1690

Weight loss measurements of carbon steel in 1MHCl solution containing various concentrations of eruca sativa seeds extract after immersion time for 90min at 45, 55 °C,

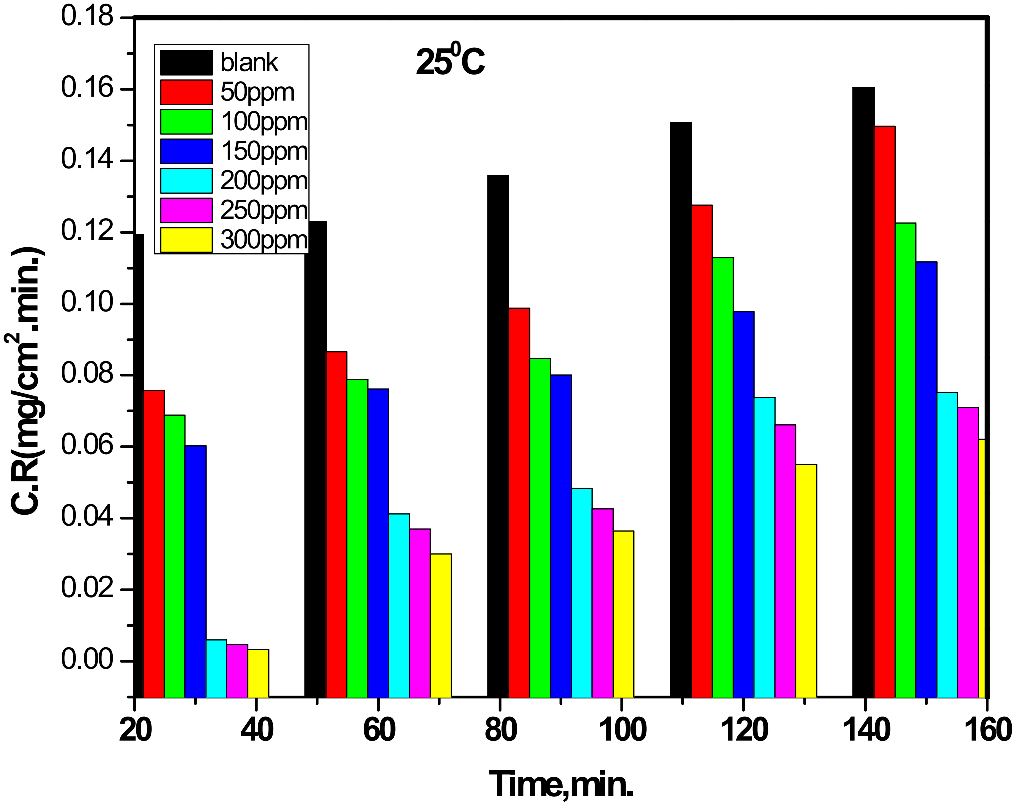

Figure S1 depicts the corrosion rate of a carbon steel electrode dipped in a 1 M HCl solution containing various concentrations of eruca sativa seeds extract at 25°C as a function of time.

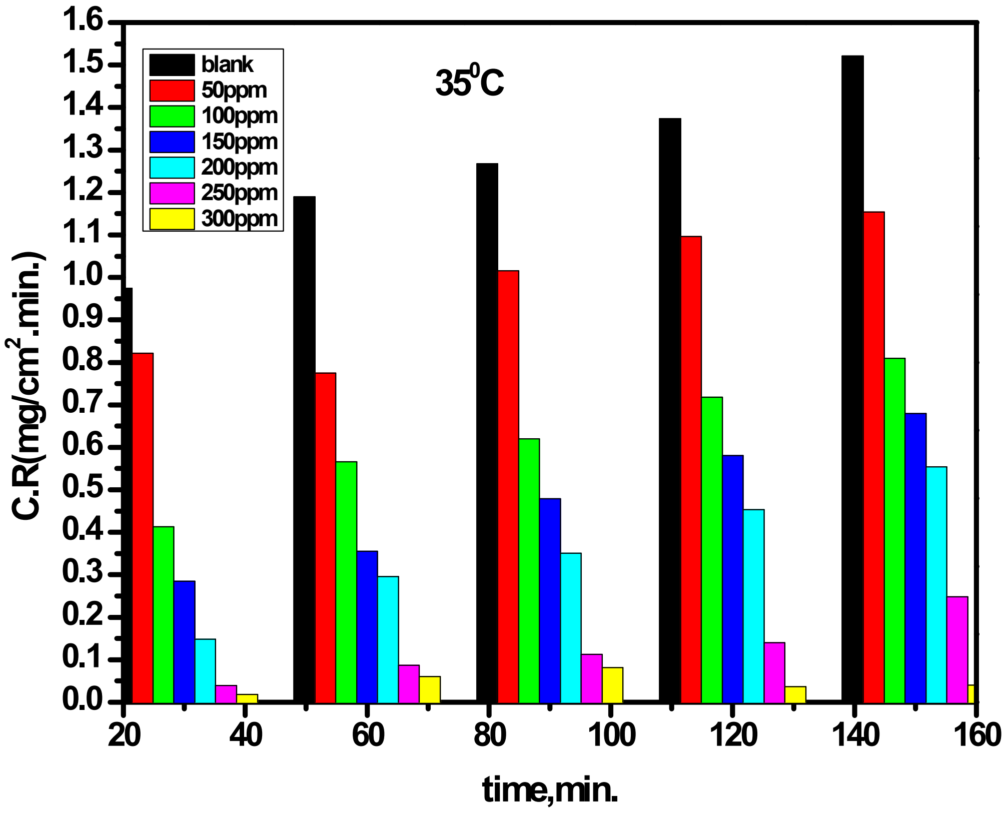

Figure S2 depicts the corrosion rate of a carbon steel electrode dipped in a 1 M HCl solution containing various concentrations of eruca sativa seeds extract at 35°C as a function of time.

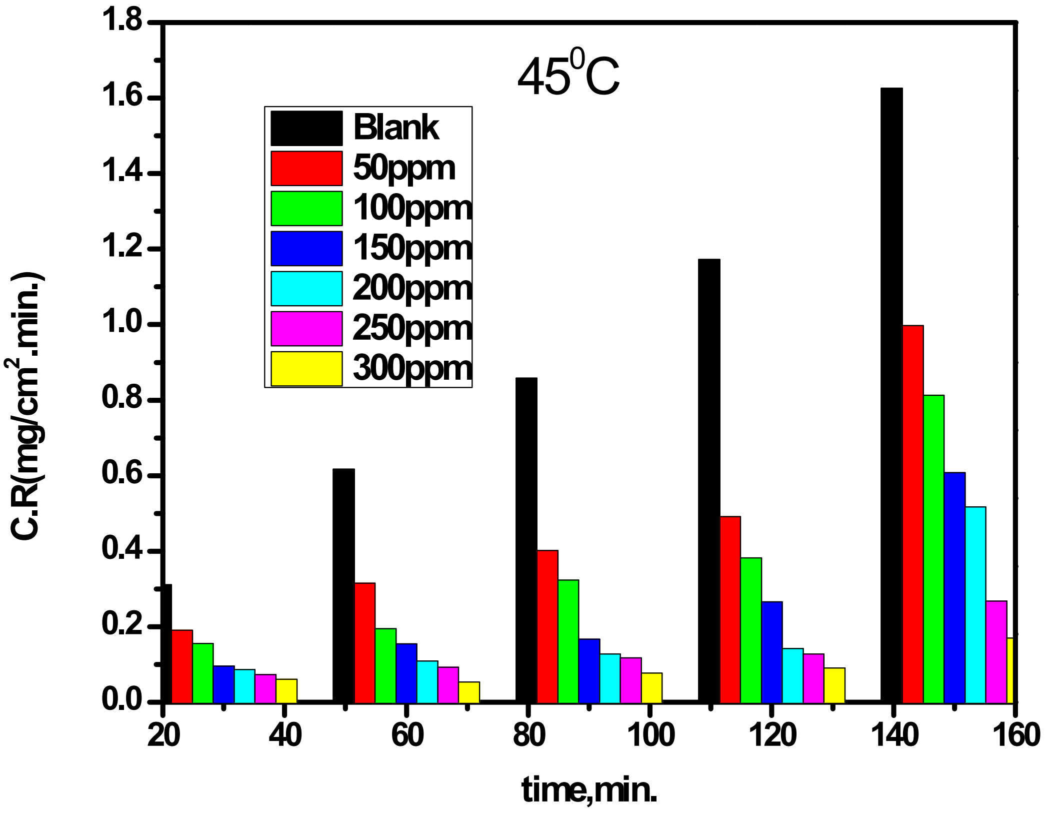

Figure S3 depicts the corrosion rate of a carbon steel electrode dipped in a 1 M HCl solution containing various concentrations of eruca sativa seeds extract at 45°C as a function of time.

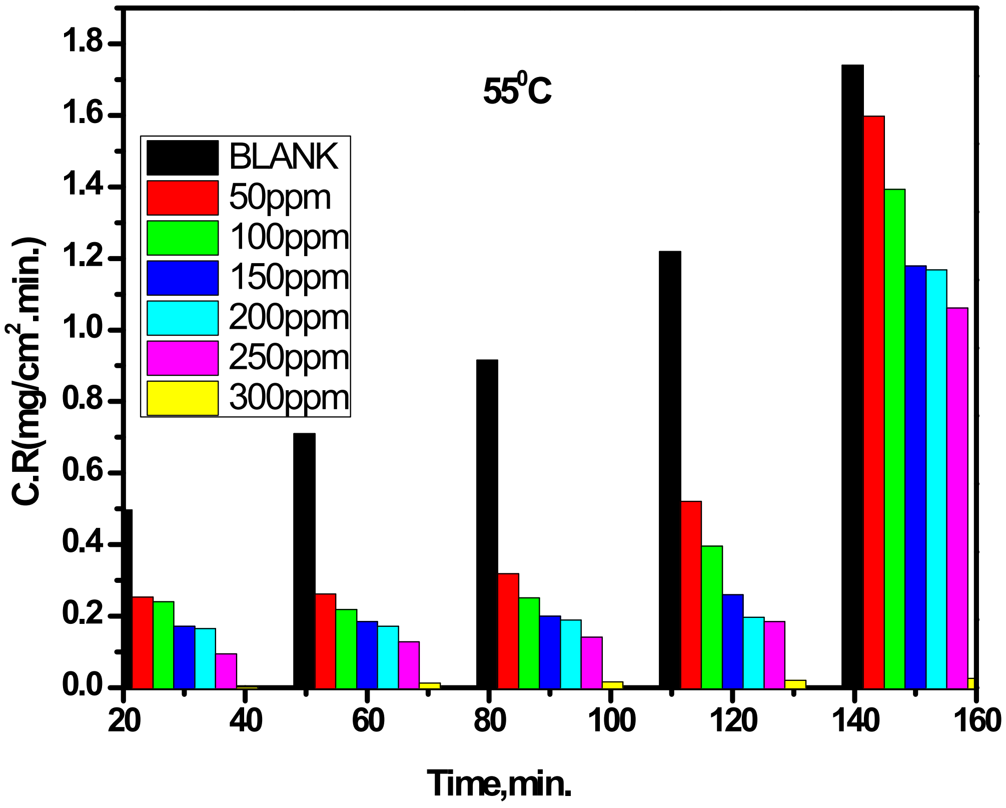

Figure S4 depicts the corrosion rate of a carbon steel electrode dipped in a 1 M HCl solution .containing various concentrations of eruca sativa seeds extract at 55°C as a function of time

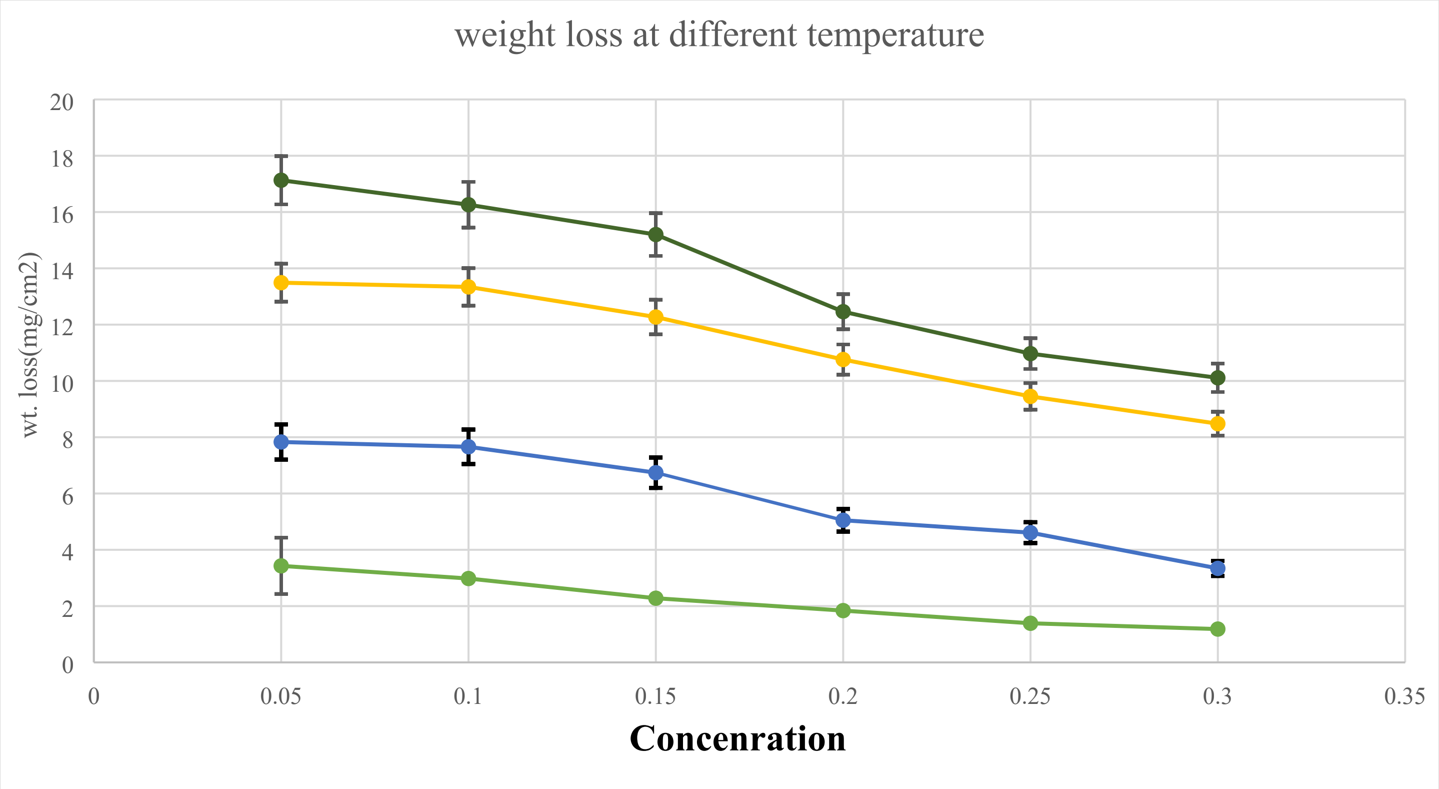

Fig. S5. Standard deviation of weight loss results of carbon steel electrode dipped in 1 M HCl solution with different concentrations (0.05-0.30g/L) of eruca sativa seeds, after a 90-minute immersion time at various temperatures (25-55°C)..

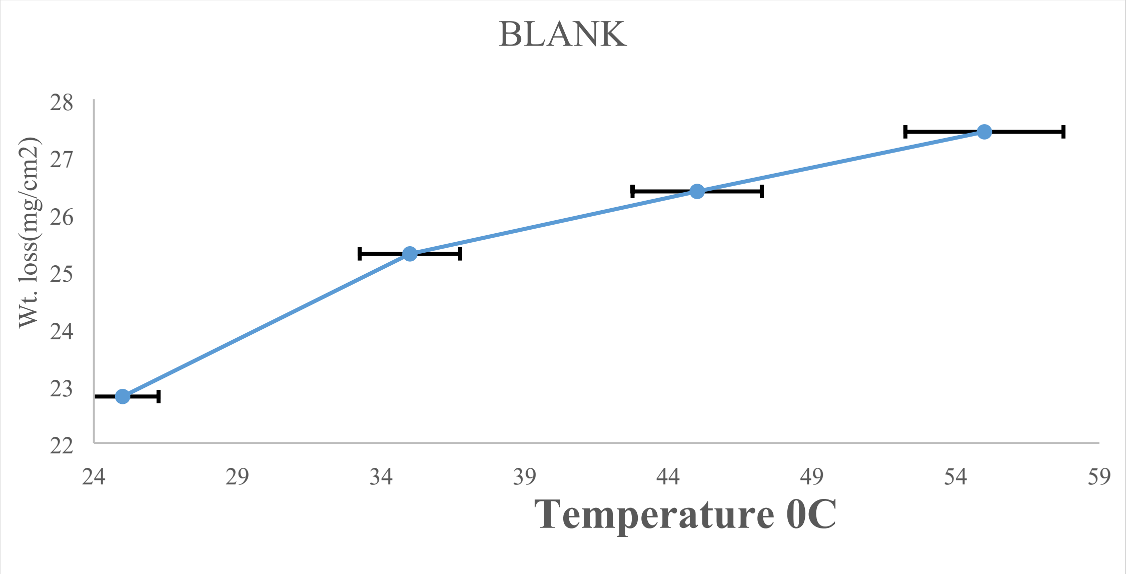

Fig. S6. Standard deviation of weight loss results of carbon steel electrode dipped in 1 M HCl solution without different concentrations of eruca sativa seeds, after a 90-minute immersion time at various temperatures (25-55°C).

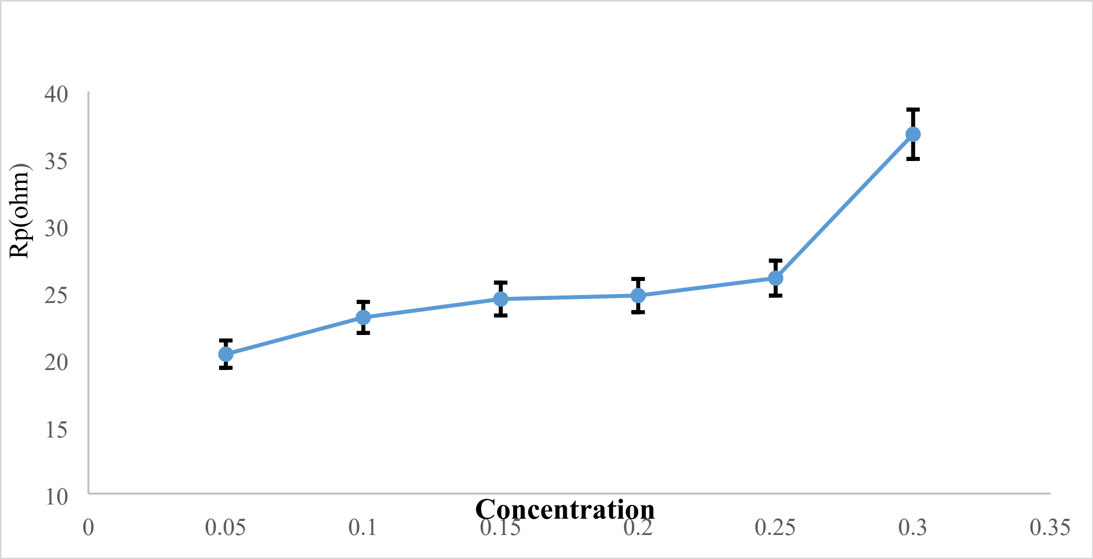

Fig. S7. Standard deviation of resistance polarization results of carbon steel electrode dipped in 1 M HCl solution with different concentrations (0.05-0.30g/L) of eruca sativa seeds, at (25°C)..

Adsorption isotherms

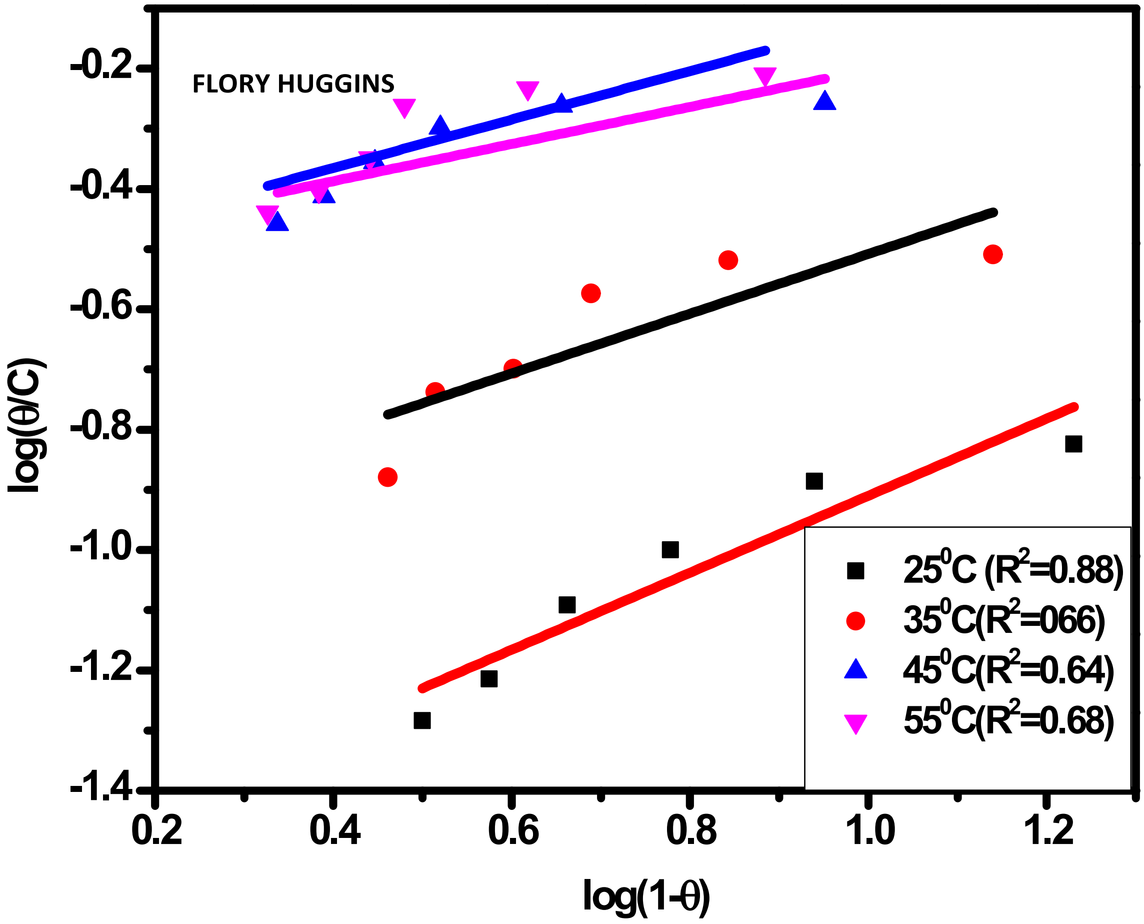

Fig. S8. Flory- Huggins adsorption isotherm plotted as logθ/C vs. log (1-θ) of eruca sativa seeds extract for corrosion of carbon steel in 1 M HCl solution at 25 °C.

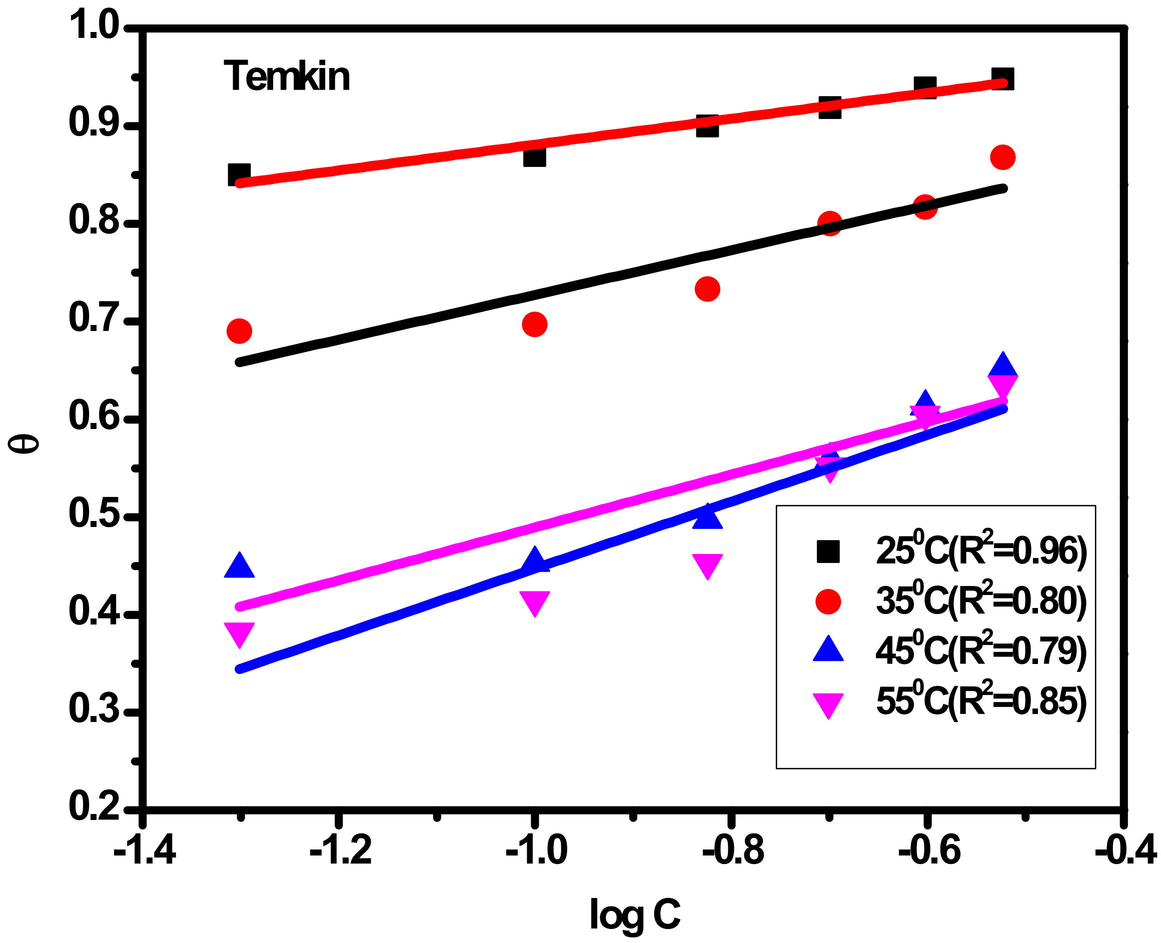

Fig. S9. Temkin adsorption isotherm plotted as θ vs. log C of eruca sativa seeds extract for corrosion of carbon steel in 1 M HCl solution at 25 °C.

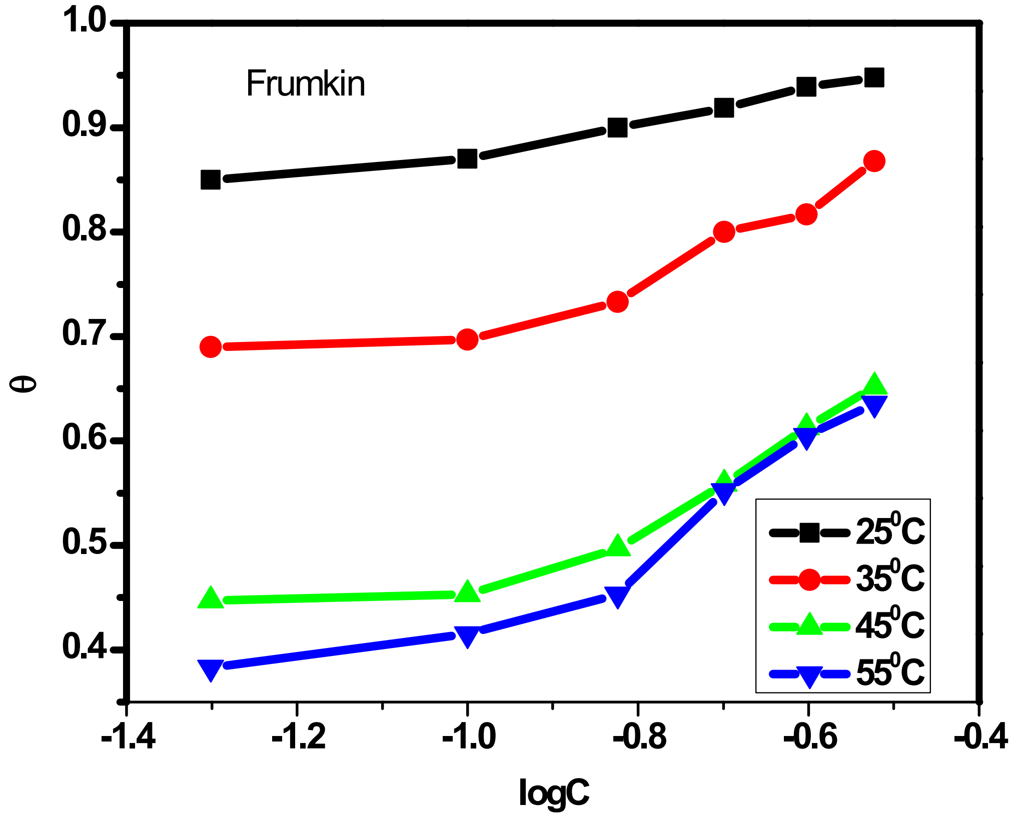

Fig. S10. Frumkin adsorption isotherm plotted as θ vs. log C of eruca sativa seeds extract for corrosion of carbon steel in 1 M HCl solution at 25 °C.

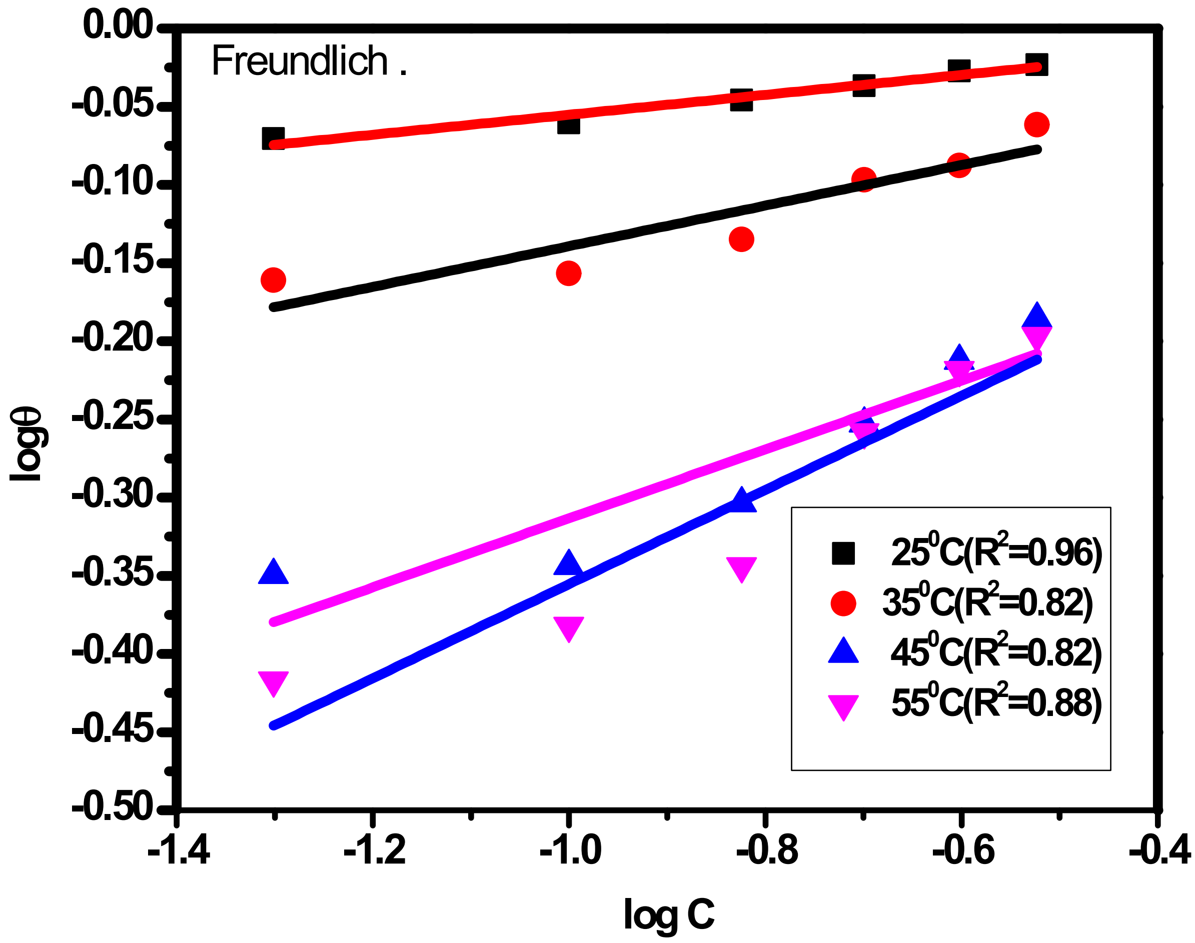

Fig. S11. Freundlich adsorption isotherm plotted as log θ vs. log C of eruca sativa seeds extract for corrosion of carbon steel in 1 M HCl solution at 25 °C.

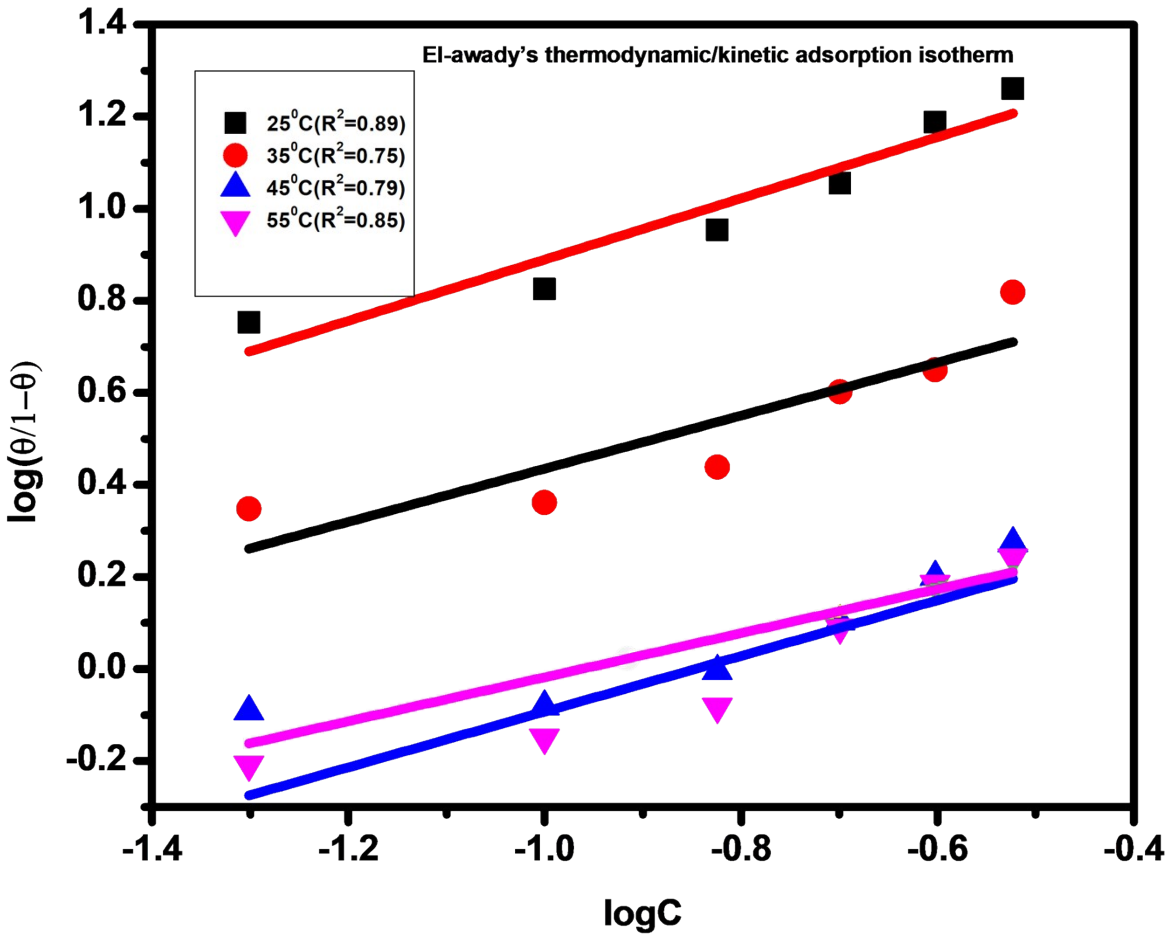

Fig. S12. Elawady s adsorption isotherm plotted as log(θ/1-θ)vs. log C of eruca sativa seeds extract for corrosion of carbon steel in 1 M HCl solution at 25 °C.

TableS3

Quercetin(neutral)

Fukui Indices for Nucleophilic Attack (Fukui(+))

atom	Mulliken	Hirshfeld
C (1)	0.104	0.078
C (2)	0.026	0.046
O (3)	0.029	0.032
C (4)	0.112	0.096
O (5)	0.120	0.121
C (6)	0.005	0.024
C (7)	0.050	0.040
O (8)	0.027	0.031
C (9)	0.028	0.032
C (10)	0.031	0.038
O (11)	0.028	0.030
C (12)	0.034	0.032
C (13)	0.021	0.026
O (14)	0.039	0.049
C (15)	0.007	0.020
C (16)	0.028	0.027
C (17)	0.014	0.017
O (18)	0.022	0.010
C (19)	0.024	0.031
O (20)	0.026	0.030
C (21)	0.018	0.022
C (22)	0.016	0.028
H (23)	0.020	0.015
H (24)	0.014	0.014
H (25)	0.028	0.017
H (26)	0.011	0.012
H (27)	0.029	0.019
H (28)	0.027	0.013
H (29)	0.003	0.003
H (30)	0.007	0.019
H (31)	0.021	0.013
H (32)	0.030	0.017

Mulliken atomic charges:

	charge	spin
C (1)	0.264	0.000
C (2)	0.223	0.000
O (3)	-0.503	0.000
C (4)	0.316	0.000
O (5)	-0.578	0.000
C (6)	-0.058	0.000
C (7)	0.318	0.000
O (8)	-0.468	0.000
C (9)	-0.171	0.000
C (10)	0.354	0.000
O (11)	-0.478	0.000
C (12)	-0.177	0.000
C (13)	0.330	0.000
O (14)	-0.494	0.000
C (15)	0.014	0.000

C (16)	-0.085	0.000
C (17)	0.283	0.000
O (18)	-0.545	0.000
C (19)	0.308	0.000
O (20)	-0.484	0.000
C (21)	-0.084	0.000
C (22)	-0.080	0.000
H (23)	0.272	0.000
H (24)	0.285	0.000
H (25)	0.062	0.000
H (26)	0.291	0.000
H (27)	0.052	0.000
H (28)	0.049	0.000
H (29)	0.303	0.000
H (30)	0.286	0.000
H (31)	0.050	0.000
H (32)	0.049	0.000

Fukui Indices for Electrophilic Attack (Fukui(-))

atom	Mulliken	Hirshfeld
C (1)	0.053	0.063
C (2)	0.087	0.081
O (3)	0.102	0.096
C (4)	0.026	0.030
O (5)	0.065	0.061
C (6)	0.020	0.020
C (7)	0.032	0.033
O (8)	0.035	0.037
C (9)	0.034	0.033
C (10)	0.019	0.030
O (11)	0.028	0.029
C (12)	0.058	0.058
C (13)	0.014	0.019
O (14)	0.046	0.046
C (15)	0.002	0.023
C (16)	0.033	0.028
C (17)	0.018	0.023
O (18)	0.001	0.016
C (19)	0.041	0.035
O (20)	0.037	0.033
C (21)	0.019	0.026
C (22)	0.028	0.025
H (23)	0.025	0.030
H (24)	0.015	0.016
H (25)	0.026	0.017
H (26)	0.011	0.012
H (27)	0.036	0.023
H (28)	0.023	0.017
H (29)	0.012	0.012
H (30)	0.013	0.002
H (31)	0.023	0.015
H (32)	0.020	0.012

Quercetin(protonated)

Fukui Indices for Nucleophilic Attack (Fukui(+))

atom	Mulliken	Hirshfeld
C (1)	0.096	0.075
C (2)	0.037	0.050
O (3)	0.036	0.036
C (4)	0.128	0.114
O (5)	0.140	0.140
C (6)	0.001	0.026
C (7)	0.048	0.038
O (8)	0.028	0.032
C (9)	0.024	0.029
C (10)	0.040	0.046
O (11)	0.036	0.038
C (12)	0.030	0.031

C (13)	0.043	0.034
O (14)	0.013	0.026
C (15)	-0.009	0.011
C (16)	0.021	0.021
C (17)	0.014	0.015
O (18)	0.017	0.012
C (19)	0.020	0.023
O (20)	0.019	0.020
C (21)	0.014	0.018
C (22)	0.018	0.021
H (23)	0.017	0.016
H (24)	0.015	0.015
H (25)	0.027	0.017
H (26)	0.013	0.014
H (27)	0.028	0.018
H (28)	0.019	0.018
H (29)	0.021	0.012
H (30)	0.004	0.005
H (31)	0.003	0.008
H (32)	0.017	0.011
H (33)	0.022	0.012

Mulliken atomic charges:

	charge	spin
C (1)	0.250	0.000
C (2)	0.276	0.000
O (3)	-0.464	0.000
C (4)	0.338	0.000
O (5)	-0.498	0.000
C (6)	-0.032	0.000
C (7)	0.337	0.000
O (8)	-0.444	0.000
C (9)	-0.146	0.000
C (10)	0.365	0.000
O (11)	-0.450	0.000
C (12)	-0.152	0.000
C (13)	0.351	0.000
O (14)	-0.429	0.000
C (15)	-0.014	0.000
C (16)	-0.077	0.000
C (17)	0.295	0.000
O (18)	-0.523	0.000
C (19)	0.314	0.000
O (20)	-0.469	0.000
C (21)	-0.070	0.000
C (22)	-0.055	0.000
H (23)	0.286	0.000
H (24)	0.297	0.000
H (25)	0.081	0.000
H (26)	0.301	0.000
H (27)	0.092	0.000
H (28)	0.367	0.000
H (29)	0.064	0.000
H (30)	0.302	0.000
H (31)	0.292	0.000
H (32)	0.060	0.000
H (33)	0.057	0.000

Fukui Indices for Electrophilic Attack (Fukui(-))

atom	Mulliken	Hirshfeld
C (1)	0.044	0.038
C (2)	0.050	0.051
O (3)	0.064	0.062
C (4)	0.015	0.016
O (5)	0.036	0.035
C (6)	0.015	0.016
C (7)	0.014	0.015
O (8)	0.017	0.018
C (9)	0.014	0.012

C (10)	0.010	0.015
O (11)	0.018	0.018
C (12)	0.024	0.024
C (13)	0.006	0.009
O (14)	0.000	0.013
C (15)	0.040	0.061
C (16)	0.040	0.037
C (17)	0.051	0.056
O (18)	0.041	0.046
C (19)	0.060	0.069
O (20)	0.099	0.094
C (21)	0.045	0.051
C (22)	0.043	0.049
H (23)	0.023	0.024
H (24)	0.008	0.008
H (25)	0.011	0.007
H (26)	0.006	0.007
H (27)	0.016	0.010
H (28)	0.019	0.013
H (29)	0.037	0.023
H (30)	0.022	0.022
H (31)	0.031	0.029
H (32)	0.041	0.027
H (33)	0.039	0.025

Table S4

Isorhamnetin(neutral)

Fukui Indices for Nucleophilic Attack (Fukui(+))

atom	Mulliken	Hirshfeld
C (1)	0.090	0.078
C (2)	0.035	0.045
C (3)	0.109	0.096
C (4)	0.007	0.024
C (5)	0.051	0.040
C (6)	0.029	0.033
C (7)	0.032	0.039
C (8)	0.033	0.032
C (9)	0.023	0.025
O (10)	0.040	0.044
O (11)	0.124	0.122
C (12)	0.001	0.020
C (13)	0.036	0.028
C (14)	0.013	0.018
C (15)	0.034	0.035
C (16)	0.017	0.022
C (17)	0.026	0.030
O (18)	0.013	0.007
C (19)	-0.007	0.004
O (20)	0.025	0.028
O (21)	0.029	0.030
O (22)	0.028	0.031
O (23)	0.031	0.031
H (24)	0.026	0.017
H (25)	0.028	0.018
H (26)	0.020	0.013
H (27)	0.020	0.012
H (28)	0.026	0.017
H (29)	0.007	0.003
H (30)	0.005	0.004
H (31)	0.005	0.003
H (32)	0.009	0.016
H (33)	0.010	0.012

H (34)	0.012	0.014
H (35)	0.013	0.012

Mulliken atomic charges:

	charge	spin
C (1)	0.266	0.000
C (2)	0.219	0.000
C (3)	0.315	0.000
C (4)	-0.059	0.000
C (5)	0.317	0.000
C (6)	-0.171	0.000
C (7)	0.355	0.000
C (8)	-0.177	0.000
C (9)	0.329	0.000
O (10)	-0.496	0.000
O (11)	-0.580	0.000
C (12)	0.016	0.000
C (13)	-0.132	0.000
C (14)	0.326	0.000
C (15)	0.296	0.000
C (16)	-0.087	0.000
C (17)	-0.098	0.000
O (18)	-0.562	0.000
C (19)	0.189	0.000
O (20)	-0.490	0.000
O (21)	-0.479	0.000
O (22)	-0.468	0.000
O (23)	-0.504	0.000
H (24)	0.061	0.000
H (25)	0.052	0.000
H (26)	0.058	0.000
H (27)	0.048	0.000
H (28)	0.048	0.000
H (29)	0.055	0.000
H (30)	0.055	0.000
H (31)	0.057	0.000
H (32)	0.297	0.000
H (33)	0.290	0.000
H (34)	0.286	0.000
H (35)	0.269	0.000

Fukui Indices for Electrophilic Attack (Fukui(-))

atom	Mulliken	Hirshfeld
C (1)	0.055	0.051
C (2)	0.069	0.073
C (3)	0.025	0.026
C (4)	0.014	0.017
C (5)	0.023	0.023
C (6)	0.023	0.023
C (7)	0.016	0.023
C (8)	0.041	0.040
C (9)	0.009	0.015
O (10)	0.030	0.038
O (11)	0.055	0.054
C (12)	0.022	0.035
C (13)	0.024	0.025
C (14)	0.031	0.032
C (15)	0.046	0.048
C (16)	0.026	0.034
C (17)	0.032	0.040
O (18)	0.024	0.035
C (19)	-0.006	0.010
O (20)	0.057	0.054
O (21)	0.021	0.022
O (22)	0.022	0.025
O (23)	0.088	0.086
H (24)	0.020	0.012
H (25)	0.028	0.017
H (26)	0.029	0.016

H (27)	0.032	0.021
H (28)	0.033	0.018
H (29)	0.013	0.009
H (30)	0.015	0.008
H (31)	0.012	0.008
H (32)	0.017	0.012
H (33)	0.009	0.009
H (34)	0.014	0.012
H (35)	0.031	0.031

Isorhamnetin(protonated)

Fukui Indices for Nucleophilic Attack (Fukui(+))

atom	Mulliken	Hirshfeld
C (1)	0.096	0.075
C (2)	0.034	0.048
C (3)	0.126	0.112
C (4)	0.001	0.025
C (5)	0.047	0.037
C (6)	0.024	0.028
C (7)	0.039	0.045
C (8)	0.031	0.031
C (9)	0.045	0.035
O (10)	0.011	0.026
O (11)	0.136	0.137
C (12)	-0.010	0.012
C (13)	0.025	0.020
C (14)	0.012	0.013
C (15)	0.026	0.026
C (16)	0.012	0.018
C (17)	0.024	0.024
O (18)	0.007	0.010
C (19)	-0.004	0.003
O (20)	0.022	0.022
O (21)	0.036	0.037
O (22)	0.027	0.031
O (23)	0.034	0.033
H (24)	0.026	0.016
H (25)	0.027	0.018
H (26)	0.025	0.021
H (27)	0.018	0.010
H (28)	0.017	0.011
H (29)	0.021	0.013
H (30)	0.005	0.003
H (31)	0.006	0.003
H (32)	0.005	0.003
H (33)	0.008	0.008
H (34)	0.013	0.014
H (35)	0.015	0.015

Mulliken atomic charges:

	charge	spin
C (1)	0.250	0.000
C (2)	0.270	0.000
C (3)	0.338	0.000
C (4)	-0.031	0.000
C (5)	0.337	0.000
C (6)	-0.144	0.000
C (7)	0.366	0.000
C (8)	-0.151	0.000
C (9)	0.345	0.000
O (10)	-0.432	0.000
O (11)	-0.500	0.000
C (12)	-0.010	0.000
C (13)	-0.123	0.000

C (14)	0.334	0.000
C (15)	0.311	0.000
C (16)	-0.076	0.000
C (17)	-0.069	0.000
O (18)	-0.555	0.000
C (19)	0.186	0.000
O (20)	-0.472	0.000
O (21)	-0.451	0.000
O (22)	-0.444	0.000
O (23)	-0.465	0.000
H (24)	0.080	0.000
H (25)	0.092	0.000
H (26)	0.367	0.000
H (27)	0.070	0.000
H (28)	0.058	0.000
H (29)	0.056	0.000
H (30)	0.060	0.000
H (31)	0.058	0.000
H (32)	0.061	0.000
H (33)	0.303	0.000
H (34)	0.300	0.000
H (35)	0.296	0.000
H (36)	0.283	0.000

Fukui Indices for Electrophilic Attack (Fukui(-))

atom	Mulliken	Hirshfeld
C (1)	0.029	0.023
C (2)	0.044	0.042
C (3)	0.013	0.014
C (4)	0.008	0.009
C (5)	0.010	0.009
C (6)	0.009	0.008
C (7)	0.008	0.010
C (8)	0.011	0.011
C (9)	0.001	0.005
O (10)	-0.002	0.009
O (11)	0.032	0.030
C (12)	0.041	0.061
C (13)	0.045	0.042
C (14)	0.060	0.065
C (15)	0.063	0.073
C (16)	0.045	0.051
C (17)	0.052	0.063
O (18)	0.079	0.079
C (19)	-0.016	0.021
O (20)	0.098	0.093
O (21)	0.010	0.011
O (22)	0.009	0.010
O (23)	0.048	0.048
H (24)	0.008	0.005
H (25)	0.011	0.006
H (26)	0.011	0.007
H (27)	0.040	0.024
H (28)	0.044	0.028
H (29)	0.046	0.029
H (30)	0.032	0.020
H (31)	0.031	0.019
H (32)	0.024	0.015
H (33)	0.028	0.031
H (34)	0.004	0.004
H (35)	0.004	0.005
H (36)	0.021	0.020

TableS5

ASCROBIC ACID (NEUTRAL)

Fukui Indices for Nucleophilic Attack (Fukui(+))

atom	Mulliken	Hirshfeld
O (1)	0.062	0.069
C (2)	-0.030	0.027
C (3)	0.166	0.130
C (4)	0.065	0.081
C (5)	0.176	0.156
O (6)	0.181	0.183
O (7)	0.053	0.055
O (8)	0.075	0.083
C (9)	-0.006	0.015
C (10)	-0.006	0.008
O (11)	0.011	0.011
O (12)	0.026	0.023
H (13)	0.084	0.049
H (14)	0.022	0.023
H (15)	0.031	0.033
H (16)	0.032	0.015
H (17)	0.019	0.013
H (18)	0.018	0.009
H (19)	0.009	0.006
H (20)	0.009	0.011

Mulliken atomic charges:

	charge	spin
O (1)	-0.542	0.000
C (2)	0.195	0.000
C (3)	0.260	0.000
C (4)	0.215	0.000
C (5)	0.536	0.000
O (6)	-0.524	0.000
O (7)	-0.500	0.000
O (8)	-0.466	0.000
C (9)	0.217	0.000
C (10)	0.234	0.000
O (11)	-0.561	0.000
O (12)	-0.530	0.000
H (13)	0.079	0.000
H (14)	0.294	0.000
H (15)	0.303	0.000
H (16)	0.041	0.000
H (17)	0.048	0.000
H (18)	0.034	0.000
H (19)	0.282	0.000
H (20)	0.284	0.000

Fukui Indices for Electrophilic Attack (Fukui(-))

atom	Mulliken	Hirshfeld
O (1)	0.043	0.042
C (2)	-0.019	0.021
C (3)	0.122	0.117
C (4)	0.133	0.138
C (5)	0.054	0.051
O (6)	0.103	0.099
O (7)	0.178	0.168
O (8)	0.122	0.119
C (9)	-0.005	0.012
C (10)	-0.002	0.009
O (11)	0.017	0.017
O (12)	0.028	0.025
H (13)	0.058	0.034
H (14)	0.049	0.057

H (15)	0.038	0.043
H (16)	0.029	0.014
H (17)	0.018	0.010
H (18)	0.014	0.008
H (19)	0.007	0.008
H (20)	0.012	0.010

ASCROBIC ACID (PROTONATED)

Fukui Indices for Nucleophilic Attack (Fukui(+))

atom	Mulliken	Hirshfeld
O (1)	0.076	0.082
C (2)	-0.015	0.030
C (3)	0.143	0.126
C (4)	0.054	0.066
C (5)	0.170	0.151
O (6)	0.106	0.110
O (7)	0.080	0.063
O (8)	0.094	0.098
C (9)	0.000	0.019
C (10)	-0.012	0.010
O (11)	0.016	0.017
O (12)	0.028	0.023
H (13)	0.081	0.050
H (14)	0.035	0.038
H (15)	0.013	0.021
H (16)	0.030	0.040
H (17)	0.040	0.019
H (18)	0.020	0.015
H (19)	0.020	0.012
H (20)	0.010	0.003
H (21)	0.012	0.011

Mulliken atomic charges:

	charge	spin
O (1)	-0.445	0.000
C (2)	0.181	0.000
C (3)	0.322	0.000
C (4)	0.251	0.000
C (5)	0.621	0.000

O (6)	-0.368	0.000
O (7)	-0.513	0.000
O (8)	-0.395	0.000
C (9)	0.217	0.000
C (10)	0.229	0.000
O (11)	-0.544	0.000
O (12)	-0.509	0.000
H (13)	0.125	0.000
H (14)	0.334	0.000
H (15)	0.321	0.000
H (16)	0.321	0.000
H (17)	0.065	0.000
H (18)	0.058	0.000
H (19)	0.048	0.000
H (20)	0.285	0.000
H (21)	0.295	0.000

Fukui Indices for Electrophilic Attack (Fukui(-))

atom	Mulliken	Hirshfeld
O (1)	0.011	0.010
C (2)	-0.007	0.014
C (3)	0.046	0.025
C (4)	0.026	0.029
C (5)	0.026	0.020
O (6)	0.026	0.026
O (7)	-0.014	0.004
O (8)	0.019	0.022
C (9)	-0.018	0.042
C (10)	-0.016	0.061
O (11)	0.341	0.303
O (12)	0.142	0.128
H (13)	0.035	0.019
H (14)	0.007	0.010
H (15)	0.020	0.013
H (16)	0.015	0.008
H (17)	0.060	0.031
H (18)	0.085	0.048
H (19)	0.093	0.057
H (20)	0.066	0.085
H (21)	0.038	0.043

Table S6

Erucic acid (neutral)

Fukui Indices for Nucleophilic Attack (Fukui(+))

atom	Mulliken	Hirshfeld
C (1)	0.287	0.254
C (2)	-0.038	0.046
C (3)	-0.022	0.027
C (4)	-0.014	0.005
C (5)	-0.003	0.005
C (6)	-0.004	0.002
C (7)	0.001	0.001
C (8)	-0.003	0.000
C (9)	0.002	0.001
C (10)	0.001	0.000
C (11)	-0.002	-0.001
C (12)	0.001	0.000
C (13)	-0.005	0.001
C (14)	0.004	0.000
C (15)	0.002	-0.001
C (16)	0.000	0.000
C (17)	-0.001	-0.001
C (18)	-0.001	0.000

```
 C ( 19)  -0.003       0.000
 C ( 20)  -0.001       0.000
 C ( 21)  -0.002      -0.001
 C ( 22)   0.000       0.000
 O ( 23)   0.255       0.262
 O ( 24)   0.129       0.138
 H ( 25)   0.106       0.066
 H ( 26)   0.072       0.038
 H ( 27)   0.034       0.021
 H ( 28)   0.059       0.033
 H ( 29)   0.023       0.011
 H ( 30)   0.013       0.009
 H ( 31)   0.010       0.006
 H ( 32)   0.011       0.005
 H ( 33)   0.005       0.003
 H ( 34)   0.005       0.003
 H ( 35)   0.003       0.002
 H ( 36)   0.002       0.001
 H ( 37)   0.003       0.003
 H ( 38)   0.000      -0.002
 H ( 39)   0.000       0.002
 H ( 40)  -0.001      -0.002
 H ( 41)   0.002       0.002
 H ( 42)   0.000      -0.001
 H ( 43)  -0.002       0.000
 H ( 44)  -0.002      -0.003
 H ( 45)  -0.001       0.000
 H ( 46)  -0.001      -0.003
 H ( 47)   0.001       0.002
 H ( 48)   0.002       0.001
 H ( 49)   0.000       0.001
 H ( 50)   0.000       0.000
 H ( 51)   0.000       0.000
 H ( 52)   0.002       0.001
 H ( 53)  -0.002       0.000
 H ( 54)   0.000       0.000
 H ( 55)   0.002       0.000
 H ( 56)   0.001       0.000
 H ( 57)  -0.001       0.001
 H ( 58)   0.003       0.000
 H ( 59)   0.000      -0.002
 H ( 60)   0.001       0.001
 H ( 61)  -0.002      -0.001
 H ( 62)   0.002       0.000
 H ( 63)   0.001       0.001
 H ( 64)   0.000       0.000
 H ( 65)   0.000       0.001
 H ( 66)   0.065       0.061

Mulliken atomic charges:
          charge    spin

 C (  1)     0.528   0.000
  C (  2)   -0.092   0.000
  C (  3)   -0.037   0.000
  C (  4)   -0.032   0.000
  C (  5)   -0.030   0.000
  C (  6)   -0.032   0.000
  C (  7)   -0.033   0.000
  C (  8)   -0.032   0.000
  C (  9)   -0.034   0.000
  C ( 10)   -0.035   0.000
  C ( 11)   -0.028   0.000
  C ( 12)   -0.038   0.000
  C ( 13)   -0.035   0.000
  C ( 14)   -0.034   0.000
  C ( 15)   -0.041   0.000
  C ( 16)   -0.024   0.000
  C ( 17)   -0.033   0.000
  C ( 18)   -0.031   0.000
  C ( 19)   -0.029   0.000
  C ( 20)   -0.026   0.000
```

C (21)	-0.031	0.000
C (22)	-0.065	0.000
O (23)	-0.524	0.000
O (24)	-0.450	0.000
H (25)	0.056	0.000
H (26)	0.047	0.000
H (27)	0.035	0.000
H (28)	0.029	0.000
H (29)	0.021	0.000
H (30)	0.025	0.000
H (31)	0.019	0.000
H (32)	0.018	0.000
H (33)	0.016	0.000
H (34)	0.020	0.000
H (35)	0.018	0.000
H (36)	0.014	0.000
H (37)	0.015	0.000
H (38)	0.019	0.000
H (39)	0.018	0.000
H (40)	0.015	0.000
H (41)	0.017	0.000
H (42)	0.021	0.000
H (43)	0.019	0.000
H (44)	0.018	0.000
H (45)	0.020	0.000
H (46)	0.037	0.000
H (47)	0.003	0.000
H (48)	0.003	0.000
H (49)	0.034	0.000
H (50)	0.019	0.000
H (51)	0.018	0.000
H (52)	0.019	0.000
H (53)	0.019	0.000
H (54)	0.017	0.000
H (55)	0.015	0.000
H (56)	0.017	0.000
H (57)	0.016	0.000
H (58)	0.015	0.000
H (59)	0.014	0.000
H (60)	0.015	0.000
H (61)	0.017	0.000
H (62)	0.016	0.000
H (63)	0.020	0.000
H (64)	0.020	0.000
H (65)	0.018	0.000
H (66)	0.283	0.000

Fukui Indices for Electrophilic Attack (Fukui(-))

atom	Mulliken	Hirshfeld
C (1)	-0.001	0.000
C (2)	-0.002	-0.001
C (3)	-0.001	-0.001
C (4)	-0.002	0.000
C (5)	0.000	0.000
C (6)	0.002	0.001
C (7)	-0.002	0.001
C (8)	0.001	0.002
C (9)	-0.005	0.004
C (10)	-0.007	0.006
C (11)	-0.006	0.019
C (12)	-0.033	0.038
C (13)	0.180	0.206
C (14)	0.167	0.206
C (15)	-0.034	0.038
C (16)	-0.009	0.019
C (17)	-0.004	0.008
C (18)	-0.002	0.004
C (19)	0.002	0.003
C (20)	0.000	0.001

C (21)	0.002	0.001
C (22)	0.000	0.000
O (23)	0.003	0.001
O (24)	0.001	0.004
H (25)	0.001	0.002
H (26)	0.000	0.000
H (27)	0.004	-0.002
H (28)	-0.002	0.000
H (29)	0.000	-0.001
H (30)	0.001	0.000
H (31)	0.000	-0.001
H (32)	0.000	0.001
H (33)	0.000	0.000
H (34)	0.000	0.000
H (35)	0.001	0.000
H (36)	0.002	0.001
H (37)	0.002	-0.001
H (38)	0.004	0.004
H (39)	0.006	0.001
H (40)	0.008	0.006
H (41)	0.012	0.005
H (42)	0.014	0.009
H (43)	0.029	0.014
H (44)	0.037	0.021
H (45)	0.060	0.032
H (46)	0.090	0.062
H (47)	0.111	0.067
H (48)	0.111	0.069
H (49)	0.087	0.057
H (50)	0.057	0.030
H (51)	0.036	0.019
H (52)	0.028	0.014
H (53)	0.016	0.008
H (54)	0.014	0.007
H (55)	0.005	0.004
H (56)	0.007	0.004
H (57)	0.005	0.001
H (58)	0.001	0.002
H (59)	0.002	0.003
H (60)	0.001	0.000
H (61)	0.003	0.001
H (62)	-0.001	0.000
H (63)	0.000	0.000
H (64)	0.000	0.000
H (65)	0.001	0.000
H (66)	0.001	0.001

ERUCIC ACID (PROTONATED)

Fukui Indices for Nucleophilic Attack (Fukui(+))

atom	Mulliken	Hirshfeld
C (1)	0.395	0.313
C (2)	-0.038	0.070
C (3)	-0.016	0.052
C (4)	-0.015	0.009
C (5)	-0.006	0.007
C (6)	-0.003	0.004
C (7)	-0.001	0.002
C (8)	0.000	0.001
C (9)	0.000	0.001
C (10)	0.000	0.001
C (11)	0.000	0.000
C (12)	0.000	0.000
C (13)	0.000	0.000
C (14)	0.001	0.000
C (15)	0.000	0.000

C (16)	0.000	0.000
C (17)	0.000	0.000
C (18)	0.000	0.000
C (19)	0.000	0.000
C (20)	0.000	0.000
C (21)	0.000	0.000
C (22)	0.000	0.000
O (23)	0.253	0.280
O (24)	0.000	0.000
H (25)	0.092	0.062
H (26)	0.101	0.069
H (27)	0.074	0.044
H (28)	0.059	0.032
H (29)	0.027	0.014
H (30)	0.028	0.013
H (31)	0.013	0.007
H (32)	0.013	0.007
H (33)	0.007	0.003
H (34)	0.006	0.003
H (35)	0.003	0.001
H (36)	0.003	0.001
H (37)	0.001	0.001
H (38)	0.001	0.001
H (39)	0.001	0.000
H (40)	0.001	0.000
H (41)	0.000	0.000
H (42)	0.000	0.000
H (43)	0.000	0.000
H (44)	0.000	0.000
H (45)	0.000	0.000
H (46)	0.000	0.000
H (47)	0.000	0.000
H (48)	0.000	0.000
H (49)	-0.001	0.000
H (50)	0.000	0.000
H (51)	0.000	0.000
H (52)	0.000	0.000
H (53)	0.000	0.000
H (54)	0.000	0.000
H (55)	0.000	0.000
H (56)	0.000	0.000
H (57)	0.000	0.000
H (58)	0.000	0.000
H (59)	0.000	0.000
H (60)	-0.001	0.000
H (61)	0.000	0.000
H (62)	0.000	0.000
H (63)	0.000	0.000
H (64)	0.000	0.000
H (65)	0.000	0.000
H (66)	0.000	0.000
H (67)	0.000	0.000

Mulliken atomic charges:

	charge	spin
C (1)	0.579	0.000
C (2)	-0.149	0.000
C (3)	-0.041	0.000
C (4)	-0.043	0.000
C (5)	-0.033	0.000
C (6)	-0.034	0.000
C (7)	-0.032	0.000
C (8)	-0.032	0.000
C (9)	-0.031	0.000
C (10)	-0.033	0.000
C (11)	-0.015	0.000
C (12)	-0.045	0.000

C (13)	-0.030	0.000
C (14)	-0.033	0.000
C (15)	-0.043	0.000
C (16)	-0.014	0.000
C (17)	-0.032	0.000
C (18)	-0.030	0.000
C (19)	-0.029	0.000
C (20)	-0.025	0.000
C (21)	-0.030	0.000
C (22)	-0.065	0.000
O (23)	-0.082	0.000
O (24)	-0.555	0.000
H (25)	0.170	0.000
H (26)	0.173	0.000
H (27)	0.081	0.000
H (28)	0.078	0.000
H (29)	0.044	0.000
H (30)	0.045	0.000
H (31)	0.031	0.000
H (32)	0.029	0.000
H (33)	0.021	0.000
H (34)	0.022	0.000
H (35)	0.019	0.000
H (36)	0.018	0.000
H (37)	0.016	0.000
H (38)	0.017	0.000
H (39)	0.018	0.000
H (40)	0.016	0.000
H (41)	0.017	0.000
H (42)	0.018	0.000
H (43)	0.018	0.000
H (44)	0.017	0.000
H (45)	0.017	0.000
H (46)	0.030	0.000
H (47)	0.004	0.000
H (48)	0.003	0.000
H (49)	0.030	0.000
H (50)	0.017	0.000
H (51)	0.016	0.000
H (52)	0.017	0.000
H (53)	0.018	0.000
H (54)	0.017	0.000
H (55)	0.016	0.000
H (56)	0.016	0.000
H (57)	0.016	0.000
H (58)	0.016	0.000
H (59)	0.014	0.000
H (60)	0.015	0.000
H (61)	0.016	0.000
H (62)	0.016	0.000
H (63)	0.020	0.000
H (64)	0.020	0.000
H (65)	0.018	0.000
H (66)	0.277	0.000
H (67)	0.278	0.000

Fukui Indices for Electrophilic Attack (Fukui(-))

atom	Mulliken	Hirshfeld
C (1)	0.001	0.001
C (2)	-0.002	0.000
C (3)	0.001	0.000
C (4)	0.001	0.000
C (5)	-0.001	0.001
C (6)	0.000	0.001
C (7)	-0.001	0.002
C (8)	-0.002	0.003
C (9)	-0.004	0.005
C (10)	-0.004	0.009
C (11)	-0.010	0.023

C (12)	-0.030	0.035
C (13)	0.166	0.201
C (14)	0.165	0.201
C (15)	-0.030	0.035
C (16)	-0.010	0.024
C (17)	-0.005	0.010
C (18)	-0.003	0.006
C (19)	-0.002	0.003
C (20)	-0.001	0.002
C (21)	0.000	0.001
C (22)	0.000	0.001
O (23)	-0.001	0.000
O (24)	0.000	0.000
H (25)	0.004	0.002
H (26)	0.000	0.000
H (27)	-0.001	0.000
H (28)	-0.001	-0.001
H (29)	-0.003	-0.001
H (30)	0.000	0.000
H (31)	0.002	0.001
H (32)	0.001	0.000
H (33)	0.001	0.001
H (34)	0.002	0.001
H (35)	0.003	0.002
H (36)	0.003	0.001
H (37)	0.005	0.002
H (38)	0.005	0.002
H (39)	0.008	0.005
H (40)	0.009	0.005
H (41)	0.016	0.008
H (42)	0.015	0.008
H (43)	0.033	0.017
H (44)	0.038	0.019
H (45)	0.054	0.028
H (46)	0.080	0.051
H (47)	0.112	0.068
H (48)	0.112	0.069
H (49)	0.081	0.051
H (50)	0.053	0.028
H (51)	0.039	0.019
H (52)	0.034	0.017
H (53)	0.016	0.008
H (54)	0.016	0.008
H (55)	0.009	0.005
H (56)	0.009	0.004
H (57)	0.005	0.002
H (58)	0.004	0.002
H (59)	0.002	0.001
H (60)	0.003	0.001
H (61)	0.001	0.001
H (62)	0.001	0.001
H (63)	0.001	0.000
H (64)	0.001	0.000
H (65)	0.001	0.001
H (66)	0.000	0.000
H (67)	0.000	0.000

TableS7

OLEIC ACID(NEUTRAL)

Fukui Indices for Nucleophilic Attack (Fukui(+))

atom	Mulliken	Hirshfeld
C (1)	0.292	0.263
C (2)	-0.046	0.046
C (3)	-0.027	0.010
C (4)	-0.014	0.004
C (5)	-0.001	0.003

C (6)	0.002	0.002
C (7)	-0.003	0.001
C (8)	-0.005	0.001
C (9)	-0.001	0.000
C (10)	0.009	0.001
C (11)	-0.006	0.000
C (12)	0.000	-0.002
C (13)	0.002	0.000
C (14)	0.002	0.000
C (15)	-0.001	0.000
C (16)	0.002	0.000
C (17)	0.002	0.000
C (18)	0.002	0.000
O (19)	0.249	0.253
O (20)	0.144	0.155
H (21)	0.085	0.046
H (22)	0.127	0.085
H (23)	0.036	0.021
H (24)	0.035	0.018
H (25)	0.014	0.008
H (26)	0.022	0.011
H (27)	0.004	0.004
H (28)	0.008	0.005
H (29)	0.004	0.000
H (30)	0.005	0.002
H (31)	0.000	0.001
H (32)	0.002	0.002
H (33)	0.001	0.000
H (34)	0.002	-0.001
H (35)	0.000	-0.001
H (36)	0.000	-0.001
H (37)	0.003	0.004
H (38)	-0.002	-0.002
H (39)	-0.001	0.002
H (40)	-0.006	-0.005
H (41)	0.003	0.001
H (42)	-0.002	0.000
H (43)	-0.001	0.000
H (44)	0.000	0.000
H (45)	0.000	-0.001
H (46)	0.000	0.001
H (47)	-0.002	0.000
H (48)	0.002	0.002
H (49)	-0.001	0.000
H (50)	-0.001	0.000
H (51)	-0.001	0.000
H (52)	0.000	0.001
H (53)	-0.001	-0.001
H (54)	0.062	0.061

Mulliken atomic charges:

	charge	spin
C (1)	0.539	0.000
C (2)	-0.107	0.000
C (3)	-0.023	0.000
C (4)	-0.033	0.000
C (5)	-0.031	0.000
C (6)	-0.034	0.000
C (7)	-0.019	0.000
C (8)	-0.081	0.000
C (9)	-0.023	0.000
C (10)	-0.022	0.000
C (11)	-0.080	0.000
C (12)	-0.021	0.000
C (13)	-0.034	0.000
C (14)	-0.030	0.000
C (15)	-0.031	0.000
C (16)	-0.026	0.000
C (17)	-0.032	0.000
C (18)	-0.065	0.000

O (19)	-0.524	0.000
O (20)	-0.453	0.000
H (21)	0.054	0.000
H (22)	0.062	0.000
H (23)	0.029	0.000
H (24)	0.027	0.000
H (25)	0.023	0.000
H (26)	0.020	0.000
H (27)	0.021	0.000
H (28)	0.018	0.000
H (29)	0.017	0.000
H (30)	0.020	0.000
H (31)	0.021	0.000
H (32)	0.020	0.000
H (33)	0.026	0.000
H (34)	0.034	0.000
H (35)	0.018	0.000
H (36)	0.018	0.000
H (37)	0.035	0.000
H (38)	0.026	0.000
H (39)	0.020	0.000
H (40)	0.020	0.000
H (41)	0.019	0.000
H (42)	0.016	0.000
H (43)	0.016	0.000
H (44)	0.018	0.000
H (45)	0.017	0.000
H (46)	0.015	0.000
H (47)	0.015	0.000
H (48)	0.016	0.000
H (49)	0.017	0.000
H (50)	0.016	0.000
H (51)	0.020	0.000
H (52)	0.020	0.000
H (53)	0.018	0.000
H (54)	0.281	0.000

Fukui Indices for Electrophilic Attack (Fukui(-))

atom	Mulliken	Hirshfeld
C (1)	0.009	0.000
C (2)	-0.006	0.004
C (3)	0.006	0.004
C (4)	0.003	0.002
C (5)	-0.005	0.005
C (6)	-0.007	0.008
C (7)	-0.011	0.020
C (8)	-0.020	0.035
C (9)	0.172	0.209
C (10)	0.165	0.209
C (11)	-0.019	0.036
C (12)	-0.012	0.021
C (13)	-0.006	0.008
C (14)	-0.005	0.004
C (15)	-0.001	0.003
C (16)	-0.002	0.001
C (17)	-0.002	0.001
C (18)	-0.002	0.001
O (19)	0.003	0.004
O (20)	-0.013	-0.009
H (21)	0.003	0.006
H (22)	0.002	-0.002
H (23)	0.003	0.002
H (24)	0.000	-0.001
H (25)	0.002	0.002
H (26)	-0.002	-0.001
H (27)	0.010	0.004
H (28)	0.007	0.002
H (29)	0.015	0.009
H (30)	0.012	0.007
H (31)	0.032	0.015
H (32)	0.037	0.017
H (33)	0.052	0.026

H (34)	0.081	0.055
H (35)	0.113	0.073
H (36)	0.114	0.074
H (37)	0.081	0.052
H (38)	0.055	0.029
H (39)	0.037	0.017
H (40)	0.035	0.020
H (41)	0.011	0.006
H (42)	0.016	0.007
H (43)	0.009	0.004
H (44)	0.007	0.003
H (45)	0.004	0.003
H (46)	0.004	0.000
H (47)	0.004	0.001
H (48)	0.000	-0.001
H (49)	0.002	0.000
H (50)	0.002	0.000
H (51)	0.002	0.000
H (52)	0.001	0.000
H (53)	0.002	0.001
H (54)	0.003	0.002

OLEIC ACID (PROTONATED)

Fukui Indices for Nucleophilic Attack (Fukui(+))

atom	Mulliken	Hirshfeld
C (1)	0.289	0.254
C (2)	-0.039	0.047
C (3)	-0.015	0.033
C (4)	-0.012	0.006
C (5)	-0.004	0.006
C (6)	-0.002	0.003
C (7)	-0.001	0.001
C (8)	0.001	0.001
C (9)	0.000	0.000
C (10)	0.001	0.001
C (11)	0.000	0.000
C (12)	0.000	0.000
C (13)	0.000	0.000
C (14)	0.000	0.000
C (15)	0.000	0.000
C (16)	0.000	0.000
C (17)	0.000	0.000
C (18)	0.000	0.000
O (19)	0.161	0.165
O (20)	0.163	0.163
H (21)	0.073	0.041
H (22)	0.096	0.061
H (23)	0.056	0.033
H (24)	0.043	0.022
H (25)	0.015	0.007
H (26)	0.023	0.011
H (27)	0.010	0.005
H (28)	0.010	0.005
H (29)	0.004	0.002
H (30)	0.004	0.002
H (31)	0.002	0.001
H (32)	0.001	0.000
H (33)	0.001	0.001
H (34)	0.000	0.000
H (35)	0.000	0.000
H (36)	0.001	0.001
H (37)	0.000	-0.001
H (38)	0.001	0.000
H (39)	0.000	0.000
H (40)	0.000	0.000
H (41)	0.001	0.001
H (42)	0.000	0.000
H (43)	0.000	0.000

```
 H ( 44)   0.000       0.000
 H ( 45)   0.000       0.000
 H ( 46)   0.000       0.000
 H ( 47)   0.000       0.000
 H ( 48)   0.000       0.000
 H ( 49)   0.000       0.000
 H ( 50)   0.000       0.000
 H ( 51)   0.000       0.000
 H ( 52)   0.000       0.000
 H ( 53)   0.000       0.000
 H ( 54)   0.057       0.063
 H ( 55)   0.056       0.062
```

```
Mulliken atomic charges:
            charge    spin
  C (  1)   0.632   0.000
  C (  2)  -0.111  0.000
  C (  3)  -0.043  0.000
  C (  4)  -0.034  0.000
  C (  5)  -0.032  0.000
  C (  6)  -0.035  0.000
  C (  7)  -0.020  0.000
  C (  8)  -0.079  0.000
  C (  9)  -0.025  0.000
  C ( 10)  -0.022  0.000
  C ( 11)  -0.076  0.000
  C ( 12)  -0.022  0.000
  C ( 13)  -0.034  0.000
  C ( 14)  -0.031  0.000
  C ( 15)  -0.030  0.000
  C ( 16)  -0.025  0.000
  C ( 17)  -0.031  0.000
  C ( 18)  -0.065  0.000
  O ( 19)  -0.309  0.000
  O ( 20)  -0.314  0.000
  H ( 21)   0.093   0.000
  H ( 22)   0.105   0.000
  H ( 23)   0.055   0.000
  H ( 24)   0.054   0.000
  H ( 25)  0.033   0.000
  H ( 26)  0.032   0.000
  H ( 27)  0.025   0.000
  H ( 28)  0.025   0.000
  H ( 29)  0.022   0.000
  H ( 30)  0.021   0.000
  H ( 31)  0.021   0.000
  H ( 32)  0.019   0.000
  H ( 33)  0.036   0.000
  H ( 34)  0.027   0.000
  H ( 35)  0.016   0.000
  H ( 36)  0.016   0.000
  H ( 37)  0.035   0.000
  H ( 38)  0.029   0.000
  H ( 39)  0.020   0.000
  H ( 40)  0.020   0.000
  H ( 41)  0.018   0.000
  H ( 42)  0.017   0.000
  H ( 43)  0.016   0.000
  H ( 44)  0.018   0.000
  H ( 45)  0.016   0.000
  H ( 46)  0.015   0.000
  H ( 47)  0.014   0.000
  H ( 48)  0.016   0.000
  H ( 49)  0.016   0.000
  H ( 50)  0.016   0.000
  H ( 51)  0.020   0.000
  H ( 52)  0.020   0.000
  H ( 53)  0.018   0.000
  H ( 54)  0.340   0.000
  H ( 55)  0.341   0.000
```

Fukui Indices for Electrophilic Attack (Fukui(-))

atom	Mulliken	Hirshfeld
C (1)	-0.001	0.000
C (2)	0.000	0.000
C (3)	-0.001	0.001
C (4)	-0.001	0.002
C (5)	-0.004	0.004
C (6)	-0.005	0.008
C (7)	-0.009	0.021
C (8)	-0.030	0.035
C (9)	0.175	0.210
C (10)	0.176	0.210
C (11)	-0.029	0.036
C (12)	-0.011	0.018
C (13)	-0.005	0.007
C (14)	-0.003	0.004
C (15)	-0.001	0.003
C (16)	-0.001	0.001
C (17)	0.000	0.001
C (18)	0.000	0.000
O (19)	0.002	0.001
O (20)	0.002	0.001
H (21)	0.000	0.000
H (22)	0.001	0.000
H (23)	0.003	0.002
H (24)	0.001	0.000
H (25)	0.004	0.003
H (26)	0.003	0.002
H (27)	0.008	0.004
H (28)	0.007	0.004
H (29)	0.015	0.008
H (30)	0.015	0.008
H (31)	0.037	0.019
H (32)	0.031	0.017
H (33)	0.082	0.053
H (34)	0.052	0.026
H (35)	0.115	0.073
H (36)	0.113	0.072
H (37)	0.087	0.058
H (38)	0.053	0.027
H (39)	0.036	0.018
H (40)	0.028	0.014
H (41)	0.013	0.007
H (42)	0.014	0.007
H (43)	0.007	0.003
H (44)	0.007	0.004
H (45)	0.003	0.002
H (46)	0.004	0.002
H (47)	0.002	0.001
H (48)	0.002	0.001
H (49)	0.001	0.001
H (50)	0.001	0.000
H (51)	0.001	0.000
H (52)	0.000	0.000
H (53)	0.001	0.001
H (54)	-0.001	0.000
H (55)	-0.001	0.000

Table S8

GALLIC ACID(NEUTRAL)

Fukui Indices for Nucleophilic Attack (Fukui(+))

atom	Mulliken	Hirshfeld
C (1)	0.147	0.133
C (2)	0.023	0.064
C (3)	0.084	0.076
C (4)	0.026	0.041

O (5)	0.030	0.032
C (6)	0.093	0.077
O (7)	0.048	0.054
C (8)	0.026	0.040
O (9)	0.026	0.029
C (10)	0.084	0.073
O (11)	0.147	0.145
O (12)	0.068	0.078
H (13)	0.055	0.037
H (14)	0.014	0.014
H (15)	0.020	0.021
H (16)	0.014	0.015
H (17)	0.056	0.036
H (18)	0.039	0.036

Mulliken atomic charges:

	charge	spin
C (1)	0.526	0.000
C (2)	0.005	0.000
C (3)	-0.134	0.000
C (4)	0.306	0.000
O (5)	-0.498	0.000
C (6)	0.244	0.000
O (7)	-0.496	0.000
C (8)	0.303	0.000
O (9)	-0.504	0.000
C (10)	-0.142	0.000
O (11)	-0.530	0.000
O (12)	-0.458	0.000
H (13)	0.054	0.000
H (14)	0.295	0.000
H (15)	0.303	0.000
H (16)	0.293	0.000
H (17)	0.052	0.000
H (18)	0.283	0.000

Fukui Indices for Electrophilic Attack (Fukui(-))

atom	Mulliken	Hirshfeld
C (1)	0.036	0.031
C (2)	0.058	0.083
C (3)	0.048	0.047
C (4)	0.070	0.083
O (5)	0.114	0.108
C (6)	0.100	0.104
O (7)	0.128	0.122
C (8)	0.050	0.054
O (9)	0.050	0.052
C (10)	0.059	0.060
O (11)	0.056	0.056
O (12)	0.022	0.026
H (13)	0.050	0.029
H (14)	0.032	0.036
H (15)	0.037	0.041
H (16)	0.022	0.024
H (17)	0.049	0.031
H (18)	0.018	0.015

GALLIC ACID(PROTONATED)

Fukui Indices for Nucleophilic Attack (Fukui(+))

atom	Mulliken	Hirshfeld
C (1)	0.169	0.145
C (2)	-0.007	0.045
C (3)	0.091	0.076
C (4)	0.023	0.039
O (5)	0.029	0.031

C (6)	0.093	0.081
O (7)	0.056	0.063
C (8)	0.026	0.039
O (9)	0.026	0.028
C (10)	0.086	0.072
O (11)	0.088	0.093
O (12)	0.086	0.091
H (13)	0.055	0.036
H (14)	0.017	0.015
H (15)	0.025	0.025
H (16)	0.019	0.017
H (17)	0.049	0.032
H (18)	0.032	0.035
H (19)	0.037	0.037

Mulliken atomic charges:

	charge	spin
C (1)	0.615	0.000
C (2)	0.035	0.000
C (3)	-0.109	0.000
C (4)	0.323	0.000
O (5)	-0.478	0.000
C (6)	0.275	0.000
O (7)	-0.454	0.000
C (8)	0.315	0.000
O (9)	-0.484	0.000
C (10)	-0.135	0.000
O (11)	-0.381	0.000
O (12)	-0.362	0.000
H (13)	0.081	0.000
H (14)	0.303	0.000
H (15)	0.315	0.000
H (16)	0.303	0.000
H (17)	0.079	0.000
H (18)	0.323	0.000
H (19)	0.333	0.000

Fukui Indices for Electrophilic Attack (Fukui(-))

atom	Mulliken	Hirshfeld
C (1)	0.055	0.050
C (2)	0.033	0.063
C (3)	0.066	0.062
C (4)	0.070	0.090
O (5)	0.156	0.144
C (6)	0.080	0.081
O (7)	0.109	0.101
C (8)	0.039	0.040
O (9)	0.039	0.040
C (10)	0.062	0.058
O (11)	0.051	0.047
O (12)	0.047	0.043
H (13)	0.048	0.031
H (14)	0.036	0.044
H (15)	0.028	0.032
H (16)	0.013	0.017
H (17)	0.042	0.027
H (18)	0.011	0.014
H (19)	0.014	0.018

Table S9

Kaempferol (Neutral)

Indices for Nucleophilic Attack (Fukui(+))

atom	Mulliken	Hirshfeld
C (1)	0.091	0.078
C (2)	0.035	0.046
C (3)	0.110	0.096
C (4)	0.006	0.024
C (5)	0.051	0.040
C (6)	0.028	0.033
C (7)	0.030	0.038
C (8)	0.034	0.032
C (9)	0.022	0.025
O (10)	0.039	0.044
O (11)	0.120	0.120
C (12)	-0.004	0.020
C (13)	0.033	0.032
C (14)	0.014	0.022
C (15)	0.035	0.035
C (16)	0.014	0.022
C (17)	0.034	0.030
O (18)	0.024	0.026
O (19)	0.028	0.030
O (20)	0.026	0.030
O (21)	0.031	0.030
H (22)	0.028	0.018
H (23)	0.029	0.018
H (24)	0.024	0.016
H (25)	0.021	0.013
H (26)	0.021	0.013
H (27)	0.024	0.015
H (28)	0.009	0.010
H (29)	0.011	0.012
H (30)	0.015	0.015
H (31)	0.016	0.016

ken atomic chargesMULKIN

	charge	spin
C (1)	0.273	0.000
C (2)	0.215	0.000
C (3)	0.316	0.000
C (4)	-0.059	0.000
C (5)	0.317	0.000
C (6)	-0.172	0.000
C (7)	0.354	0.000
C (8)	-0.177	0.000
C (9)	0.330	0.000
O (10)	-0.495	0.000
O (11)	-0.579	0.000
C (12)	0.013	0.000
C (13)	-0.040	0.000
C (14)	-0.086	0.000
C (15)	0.343	0.000
C (16)	-0.081	0.000
C (17)	-0.076	0.000
O (18)	-0.484	0.000
O (19)	-0.479	0.000
O (20)	-0.468	0.000
O (21)	-0.504	0.000
H (22)	0.061	0.000
H (23)	0.052	0.000

H (24)	0.045	0.000
H (25)	0.050	0.000
H (26)	0.046	0.000
H (27)	0.049	0.000
H (28)	0.288	0.000
H (29)	0.290	0.000
H (30)	0.285	0.000
H (31)	0.271	0.000

Indices for Electrophilic Attack (Fukui(-))

atom	Mulliken	Hirshfeld
C (1)	0.065	0.061
C (2)	0.080	0.082
C (3)	0.027	0.029
C (4)	0.018	0.020
C (5)	0.030	0.031
C (6)	0.031	0.030
C (7)	0.020	0.029
C (8)	0.055	0.055
C (9)	0.012	0.018
O (10)	0.040	0.047
O (11)	0.064	0.061
C (12)	0.016	0.025
C (13)	0.026	0.027
C (14)	0.023	0.028
C (15)	0.027	0.035
C (16)	0.025	0.029
C (17)	0.011	0.023
O (18)	0.041	0.040
O (19)	0.028	0.028
O (20)	0.034	0.036
O (21)	0.100	0.097
H (22)	0.024	0.015
H (23)	0.035	0.022
H (24)	0.027	0.015
H (25)	0.023	0.015
H (26)	0.024	0.016
H (27)	0.027	0.015
H (28)	0.013	0.014
H (29)	0.011	0.012
H (30)	0.014	0.015
H (31)	0.029	0.029

Kaempferol(protonated)

Fukui Indices for Nucleophilic Attack (Fukui(+))

atom	Mulliken	Hirshfeld
C (1)	0.103	0.076
C (2)	0.031	0.049
C (3)	0.127	0.113
C (4)	0.002	0.025
C (5)	0.048	0.038
C (6)	0.024	0.029
C (7)	0.040	0.046
C (8)	0.029	0.031
C (9)	0.042	0.033
O (10)	0.009	0.025
O (11)	0.137	0.138
C (12)	-0.007	0.012
C (13)	0.022	0.024
C (14)	0.013	0.018
C (15)	0.023	0.025
C (16)	0.013	0.018
C (17)	0.020	0.023
O (18)	0.021	0.022
O (19)	0.035	0.037
O (20)	0.028	0.032

O (21)	0.034	0.034
H (22)	0.027	0.017
H (23)	0.028	0.018
H (24)	0.020	0.017
H (25)	0.021	0.012
H (26)	0.017	0.011
H (27)	0.017	0.011
H (28)	0.021	0.012
H (29)	0.008	0.008
H (30)	0.015	0.015
H (31)	0.015	0.015
H (32)	0.017	0.017

Mulliken atomic charges:

	charge	spin
C (1)	0.257	0.000
C (2)	0.268	0.000
C (3)	0.339	0.000
C (4)	-0.033	0.000
C (5)	0.335	0.000
C (6)	-0.146	0.000
C (7)	0.365	0.000
C (8)	-0.151	0.000
C (9)	0.349	0.000
O (10)	-0.430	0.000
O (11)	-0.500	0.000
C (12)	-0.016	0.000
C (13)	-0.031	0.000
C (14)	-0.076	0.000
C (15)	0.357	0.000
C (16)	-0.069	0.000
C (17)	-0.045	0.000
O (18)	-0.466	0.000
O (19)	-0.451	0.000
O (20)	-0.444	0.000
O (21)	-0.466	0.000
H (22)	0.081	0.000
H (23)	0.091	0.000
H (24)		
H (25)	0.059	0.000
H (26)	0.367	0.000

0.061

0.000
H (
27)
0.057
0.000
H (
28)
0.058
0.000
H (
29)
0.294
0.000
H (
30)
0.301
0.000
H (
31)
0.296
0.000
H (
32)
0.286
0.000

Fukui Indices for Electrophilic Attack (Fukui(-))

atom	Mulliken	Hirshfeld
C (1)	0.044	0.045
C (2)	0.063	0.060
C (3)	0.016	0.018
C (4)	0.018	0.019
C (5)	0.017	0.018
C (6)	0.015	0.014
C (7)	0.011	0.017
C (8)	0.031	0.029
C (9)	0.008	0.012
O (10)	0.004	0.016
O (11)	0.041	0.039
C (12)	0.043	0.062
C (13)	0.037	0.041
C (14)	0.050	0.053
C (15)	0.043	0.061
C (16)	0.052	0.056
C (17)	0.029	0.037
O (18)	0.093	0.089
O (19)	0.021	0.020
O (20)	0.020	0.021
O (21)	0.076	0.073
H (22)	0.013	0.008
H (23)	0.018	0.012
H (24)	0.021	0.016
H (25)	0.036	0.022
H (26)	0.040	0.026
H (27)	0.041	0.027
H (28)	0.036	0.022
H (29)	0.026	0.029
H (30)	0.005	0.007
H (31)	0.009	0.009
H (32)	0.024	0.024

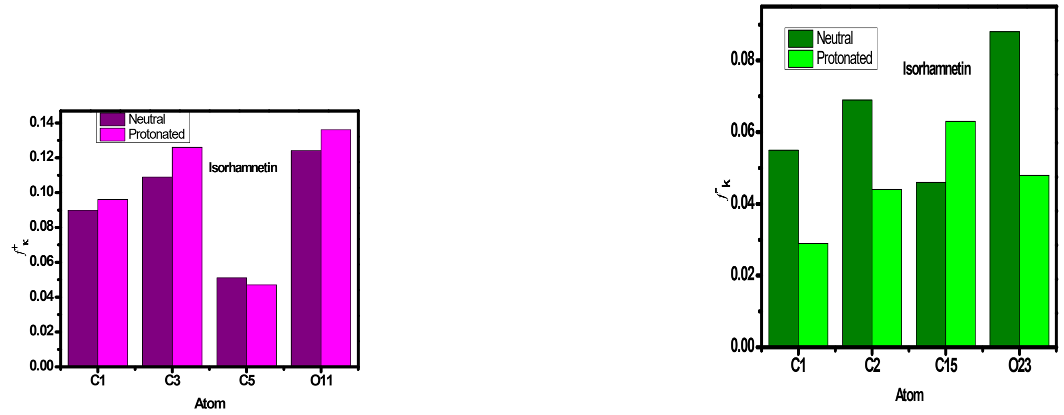

Fig. S10. Graphical representation of the Fukui indices of Isorhamnetin for the more reactive atoms in the un-protonated and protonated form.

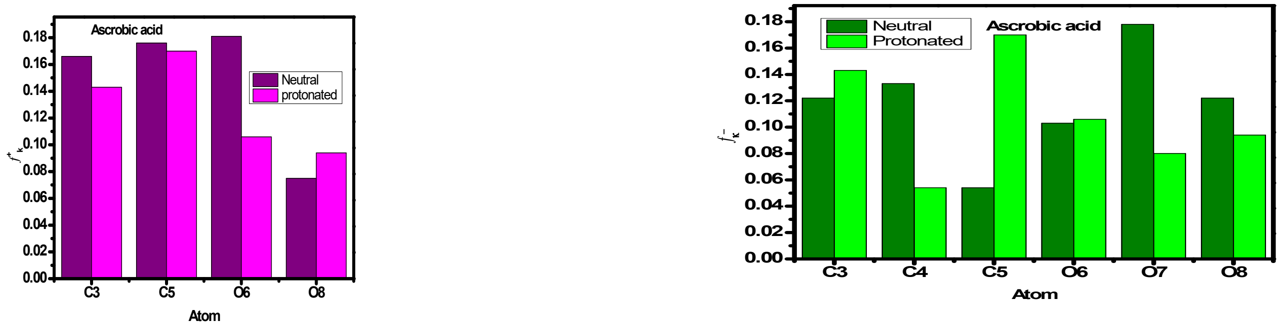

Fig. S11. Graphical representation of the Fukui indices of Ascrobic acid for the more reactive atoms in the un-protonated and protonated form.

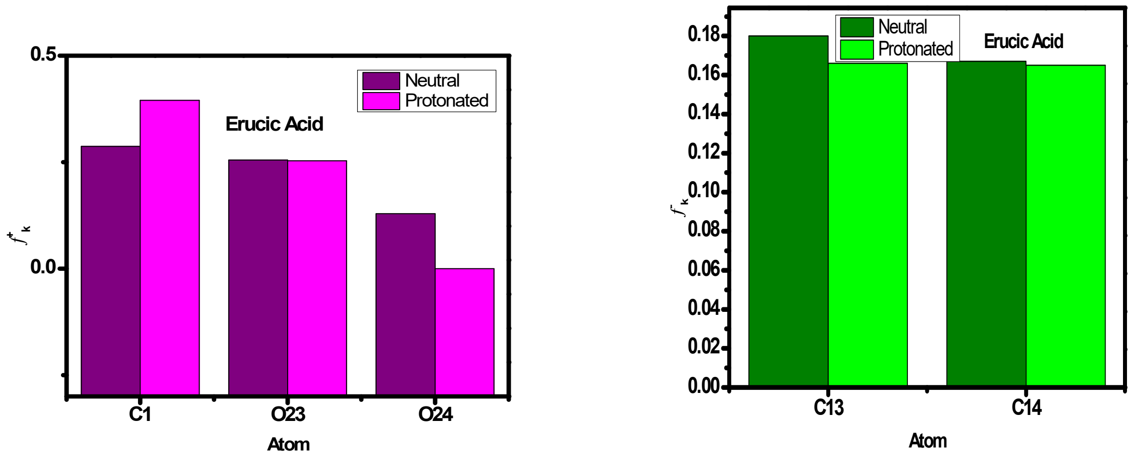

Fig. S13. Graphical representation of the Fukui indices of Erucic acid for the more reactive atoms in the un-protonated and protonated form.

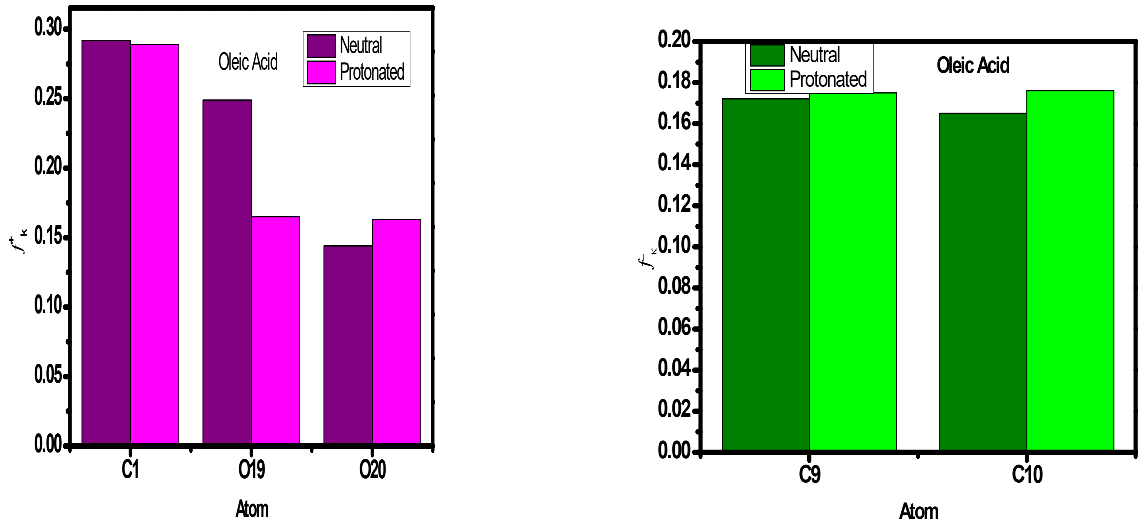

Fig. S14. Graphical representation of the Fukui indices of Oleic acid for the more reactive atoms in the un-protonated and protonated form.

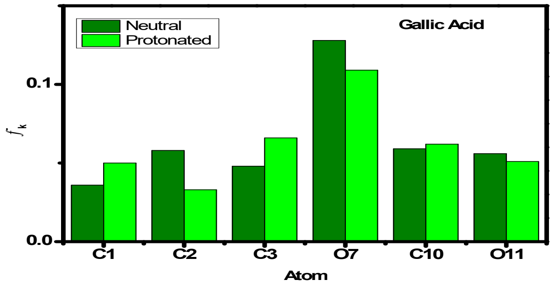

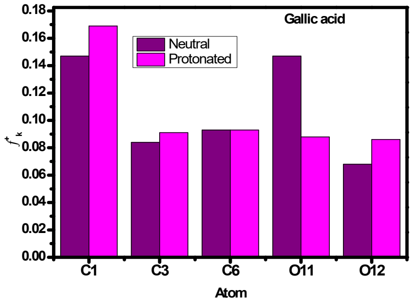

Fig. S15. Graphical representation of the Fukui indices of Gallic acid for the more reactive atoms in the un-protonated and protonated form.

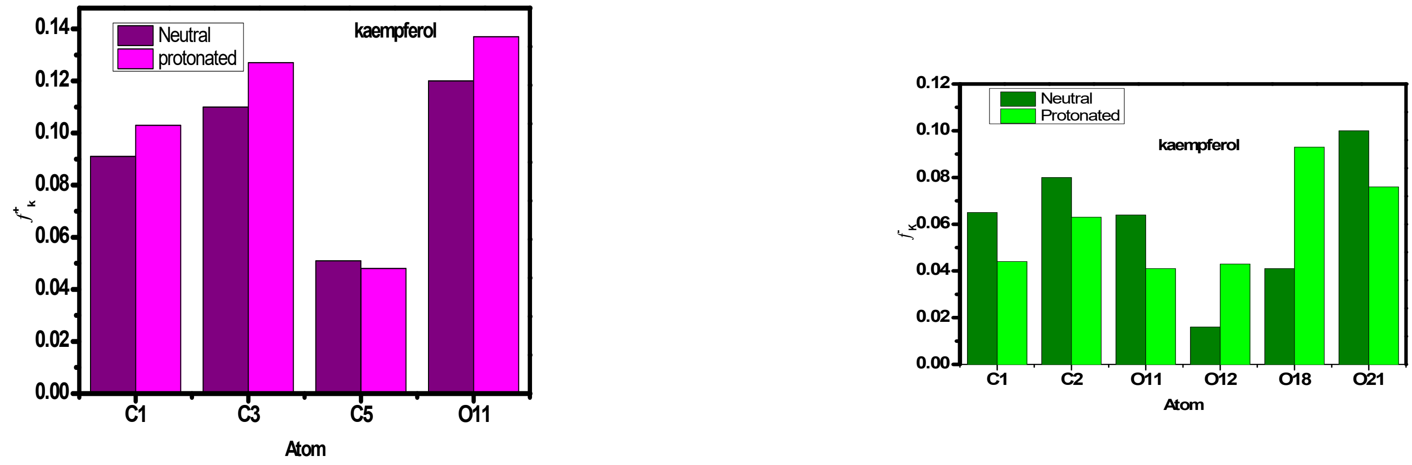

Fig. S16. Graphical representation of the Fukui indices of kaempferol acid for the more reactive atoms in the un-protonated and protonated form.
